# Reducing lifestyle risk behaviours in disadvantaged groups in high-income countries: A scoping review of systematic reviews

**DOI:** 10.1016/j.ypmed.2021.106916

**Published:** 2022-01

**Authors:** Emily South, Mark Rodgers, Kath Wright, Margaret Whitehead, Amanda Sowden

**Affiliations:** a Centre for Reviews and Dissemination, University of York, York, United Kingdom; b Department of Public Health, Policy, and Systems, University of Liverpool, Liverpool, United Kingdom

**Keywords:** Risk behaviours, Health inequalities, Disadvantaged groups

## Abstract

High prevalence of risk behaviours may exacerbate existing poor health in disadvantaged groups. We aimed to identify and bring together systematic reviews with a focus on reducing risk behaviours in disadvantaged groups and highlight where evidence is lacking. We searched MEDLINE and Embase up to October 2020, with supplementary searching in Epistemonikos and Health Systems Evidence. We included systematic reviews that reported behavioural outcomes and targeted smoking, excessive alcohol use, unhealthy diet, or physical inactivity in groups with the following characteristics: low income or low socio-economic status (SES), unemployed people, homeless people, care leavers, prisoners, refugees or asylum seeker, Gypsies, Travellers, or Roma, people with learning disabilities and people living in disadvantaged areas. Reviews that included primary studies from any high-income country were eligible. Reviews were mapped based on the disadvantaged group(s) and behaviour(s) targeted. Ninety-two reviews were included, with the majority (*n* = 63) focusing on people with low income or low SES. We identified gaps in the evidence for care leavers; Gypsies, Travellers, and Roma and limited evidence for refugees and unemployed people. Few reviews targeted alcohol use. There was limited evidence on barriers and facilitators to behaviour change. This suggests there is insufficient evidence to inform policy and practice and new reviews or primary studies may be required.

## Highlights

• We included 92 systematic reviews on risk behaviour change in disadvantaged groups.
• Most reviews focused on people with low income or socio-economic status.
• We found no reviews on care leavers or Gypsy, Traveller or Roma communities.
• Few reviews targeted refugees and asylum seekers or unemployed people.
• We found very limited evidence on reducing alcohol use in disadvantaged groups.

## 1 Introduction

Non-communicable diseases (NCDs), such as cardiovascular diseases, cancer, chronic respiratory diseases, and diabetes, account for seven of the ten most common causes of death worldwide (World Health Organization, 2020). Risk behaviours, including physical inactivity, unhealthy diet, smoking, and alcohol misuse, are major contributors to NCDs (World Health Organization, 2018). It has been estimated that 45% of years of life lost due to premature deaths in England are attributable to these four behaviours plus drug misuse (Steel et al., 2018). Tobacco use is one of the leading risk factors for death, accounting for 15.4% (8.71 million) of deaths globally in 2019 (Murray et al., 2020). A further 7.94 million deaths are attributed to diet (The Lancet, 2020a), 2.44 million to alcohol (The Lancet, 2020b), and 0.83 million to physical inactivity (The Lancet, 2020c). Inequalities in NCDs contribute to large differences in life expectancy. The gap in life expectancy between the most and least deprived areas of England is 9.4 years for males and 7.4 years for females, and there is a 19-year difference in healthy life expectancy. Much of this gap is attributable to differences in rates of heart disease, respiratory diseases, and lung cancer (Public Health England, 2019a).

Lifestyle risk behaviours are highly prevalent (Bankiewicz and Robinson, 2020; NHS Digital, 2019) and socio-economic gradients in these have been found. Smoking (Bankiewicz and Robinson, 2020), eating insufficient fruit and vegetables (Osborne and Cooper, 2018), and physical inactivity (NHS Digital, 2020) are more common in the most disadvantaged areas and households. In contrast, the proportion of people drinking over 14 units of alcohol per week is highest in the most affluent households in England (Bankiewicz and Robinson, 2020) but research has shown that for a given level of excessive drinking, the resulting health damage may be greater for disadvantaged than for advantaged socio-economic groups (Christensen et al., 2017; Katikireddi et al., 2017).

Some population groups- including homeless people (Aldridge et al., 2018; Equality and Human Rights Commission, 2016a), Gypsies, Travellers and Roma (Peters et al., 2009; Equality and Human Rights Commission, 2016b), unemployed people (McKee-Ryan et al., 2005; Norström et al., 2014; Norström et al., 2019), prisoners (Aldridge et al., 2018; Fazel and Baillargeon, 2011), refugees and asylum seekers (Equality and Human Rights Commission, 2016c), people with learning disabilities (Equality and Human Rights Commission, 2016d; National Institute for Health Research, 2020), and care leavers (HM Government, 2016; National Audit Office, 2015)- face particular disadvantage and poor health. High levels of risk behaviours in some of these groups may exacerbate their existing poor health. For example, around 80% of prisoners in the UK (Public Health England, 2015) and 70% of homeless people in the USA smoke (Baggett and Rigotti, 2010). A systematic review found low levels of physical activity in UK prisoners, and high sodium and fat intake in prisons worldwide (Herbert et al., 2012). Gypsy and Traveller communities in England have been found to have high levels of tobacco use, with 57% of males and 59% of females smoking (Peters et al., 2009). A high prevalence of risk behaviours has also been found in Roma populations (Cook et al., 2013). A 2010 review reported that risky alcohol use and smoking were more common in unemployed people (Henkel, 2011) and a study of almost 8000 job-seekers in Germany found very high prevalence of all four risk behaviours (Freyer-Adam et al., 2011). People with learning disabilities have particularly low levels of physical activity, with a systematic review reporting that only 9% of adults with learning disabilities achieved at least 150 min moderate-to-vigorous activity a week (Dairo et al., 2016).

Reducing health inequalities is a policy goal across the health system in England. Interventions are needed to address both the wider social determinants and the behavioural causes of health inequalities in order to effectively tackle them (Marteau et al., 2021). Addressing risk behaviours and health inequalities are highlighted as important challenges in the Public Health England strategy (Public Health England, 2019b) and action on prevention and inequalities is a key part of the National Health Service Long Term Plan, including prevention programmes for smoking, obesity and alcohol (NHS, 2019). A 2019 green paper signalled the UK government's intention to focus on prevention of health problems, in part by making healthy lifestyle choices easier for people (Cabinet Office and Department of Health and Social Care, 2019). There has, however, been criticism that the policy proposals in the green paper are insufficiently ambitious to address widening health inequalities (The King's Fund, 2019).

There is a growing but scattered evidence base about the effectiveness of programmes to improve risk behaviours in disadvantaged groups or those living in disadvantaged communities. There is a need to bring the available evidence together to answer questions about what works to reduce inequalities. As an initial step, we undertook a scoping review to identify existing systematic reviews and highlight where there is insufficient evidence to inform policy and new research may be required. Scoping review methods were developed to “map” the evidence on a topic with the aim of informing policy, practice and research (Arksey and O'Malley, 2005; Levac et al., 2010; Colquhoun et al., 2014). We searched for systematic reviews only, to enable a broad overview of evidence and evidence gaps relating to different groups, behaviours and intervention types.

## 2 Methods

This scoping review was informed by the framework proposed by Arksey and O'Malley (Arksey and O'Malley, 2005) and refined by Levac et al. (Levac et al., 2010), and is reported according to the Preferred Reporting Items for Systematic Reviews and Meta-Analyses extension for Scoping Reviews (Tricco et al., 2018). The protocol for this review was not registered with PROSPERO, as PROSPERO does not include scoping reviews.

### 2.1 The research question

Our scoping review question was:

What evidence is available on interventions to reduce risk behaviours in disadvantaged groups or communities?

The sub-questions were:

• Which interventions have been evaluated and implemented with which groups?
• What are the potential barriers and facilitators to adopting healthy behaviours in specific groups?
• What gaps in the evidence base exist where new or updated evidence syntheses are needed or where new primary research is required?

### 2.2 Search strategy and selection criteria

The MEDLINE and Embase databases were searched in January 2020, with an update search in October 2020 to identify reviews that had been published since the original search. Two separate search strategies were used as different structures were needed to identify reviews targeting disadvantaged groups and reviews of population-level interventions covering the whole population (not just disadvantaged groups), but which might report differential effects by relevant sub-group. One combined terms for disadvantaged groups with terms for risk behaviours and a systematic review search filter. The second combined terms for risk behaviours with terms for population-level interventions and a systematic review search filter (see Appendices 1.1–1.5).

The strategies were developed and refined through a number of pilot searches, to inform decisions on the parameters of the review, which databases to search, and which systematic review search filter to use. Searching was an iterative process, with further supplementary searching in Epistemonikos and Health Systems Evidence (Appendices 1.6 and 1.7).

Reviews of empirical evidence published between 2009 and October 2020 were eligible. We considered this timeframe appropriate given the extensive literature on this topic and the fact that systematic reviews include earlier primary studies. Reviews published in languages other than English were not eligible for practical reasons.

The eligibility criteria for the review are outlined in Table 1. We included reviews that evaluated the effects of interventions or that reported qualitative data on participants' perceptions of barriers or facilitators to behaviour change. The risk behaviours of interest were: tobacco use, unhealthy diet, physical inactivity, and excessive alcohol use. Disadvantaged groups were defined as having low income or low socio-economic status (SES), unemployed people, homeless people, care leavers, prisoners, refugees or asylum seekers, Gypsies, Travellers and Roma, people with learning disabilities, and people living in disadvantaged areas or communities. This definition was developed through discussion amongst authors, informed by the results of pilot searches and consultation with policy leads at the UK Department of Health and Social Care.

**Table 1:** Review eligibility criteria.

	Include	Exclude
Participants	Groups having low income or low SES; unemployed people; homeless people; care leavers; prisoners; refugees or asylum seekers; Gypsies, Travellers and Roma; people with learning disabilities; disadvantaged areas or communities	Exclusive focus on: Children and young people (≤18 years); clinical populations (e.g. people with diabetes); populations in low and middle-income countries.
Interventions	• Targeted at least one of: Tobacco use, unhealthy diet, physical inactivity, excessive alcohol use. • Explicitly targeted disadvantaged groups or population-level (delivered to entire country/region/area/city) with differential effects reported. • Reviews focusing on wider health or lifestyle in disadvantaged groups (i.e. not limited to behaviour change) if report change in one or more of the selected behaviours.	• Disease management • Substance use programmes (unless review is specific to alcohol misuse)
Comparators	Any or none	
Outcomes	• Change in at least one of the above behaviours • Or participants' experiences or perceptions of barriers and facilitators to changing one or more of the selected behaviours	
Study designs	• Systematic reviews (including realist reviews that followed systematic methods)^a^ • Reviews of systematic reviews • Reviews of primary and secondary evidence • Protocols of ongoing reviews	• Reviews of modelling studies only • Reviews of barriers/facilitators based on quantitative evidence only

SES Socio-economic status.

a Reviews that met basic criteria (i.e. systematic search, inclusion criteria, some form of synthesis) even if not described by authors as systematic. Included scoping reviews that met these criteria.

Interventions, such as behavioural counselling, structured exercise or education sessions, that were delivered to individuals, groups or organisations had to explicitly target one or more of the groups specified in Table 1. Reviews of interventions that were implemented at a population-level (i.e. delivered to an entire country, region, area or city) were eligible if they presented results separately for one of the specified groups or explored differential effects according to one or more of these groups.

### 2.3 Study selection

Search results were managed in Endnote X9 software at the title and abstract stage and EPPI-Reviewer software at the full text stage. Titles and abstracts were screened independently by two reviewers (ES and MR) according to the criteria outlined above. Full texts of any potentially relevant reviews were obtained and screened by one reviewer and checked by a second (ES, MR or AS). Differences between reviewers were resolved through discussion or a third reviewer.

### 2.4 Data charting process

The data extraction form was piloted by two reviewers on six reviews and revised accordingly. Data were extracted by one reviewer and checked by a second (ES or MR).

Descriptive data were extracted on the following characteristics:

• Review type
• Review aim
• Number of studies included
• Study designs eligible and included
• Setting
• Countries eligible and included
• Population targeted
• Behaviours targeted
• Intervention
• Comparator
• Outcomes and measures
• Qualitative data on barriers and facilitators to behaviour change

As this is a scoping review, we did not assess methodological quality or risk of bias.

### 2.5 Synthesis of results

We summarise and present extracted information below, adapting the method outlined by Arksey and O'Malley (Arksey and O'Malley, 2005). Using tables and charts, we have mapped the literature according to disadvantaged group and behaviour(s) targeted. Interactive online evidence maps were also produced (https://www.york.ac.uk/crd/research/public-health/evidence-summary/).

## 3 Results

**Fig. 1:**
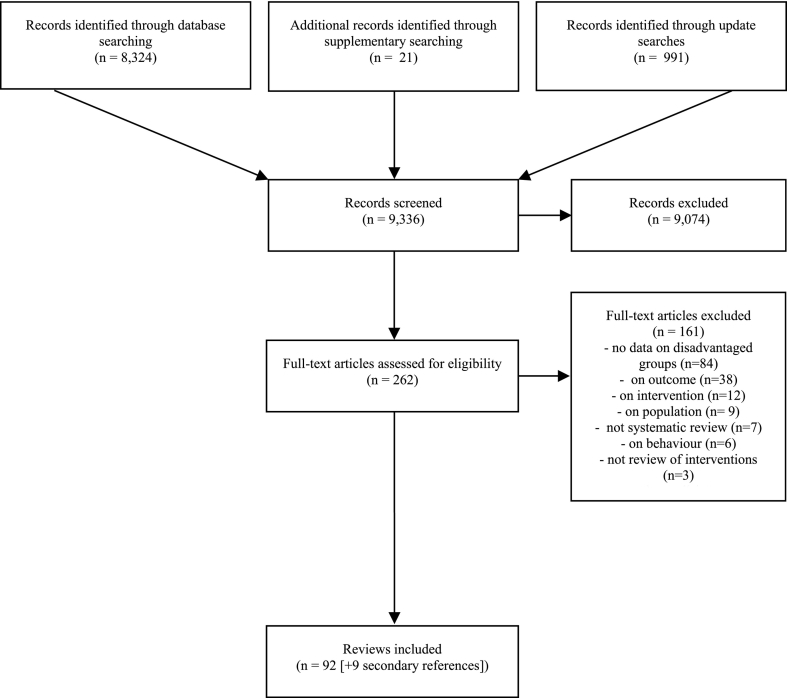
Study selection process

After de-duplication, the initial searches of Embase and MEDLINE identified 8324 records (see Fig. 1). A further 21 records were returned through supplementary searching and 991 records through update searches. Full texts were obtained for 262 titles and abstracts identified as potentially eligible. After reviewing full texts, 92 reviews were included, with nine secondary references (additional papers published on the same review).

Review characteristics are reported in Appendix tables A1 to A7. Reviews evaluated interventions targeted at disadvantaged groups (45/92; 49%), assessed the differential effects of population-level interventions (28/92; 30%), explored barriers or facilitators to behaviour change (11/92; 12%), or a combination of these (8/92; 9%).

### 3.1 Disadvantaged groups and behaviours addressed

**Fig. 2:**
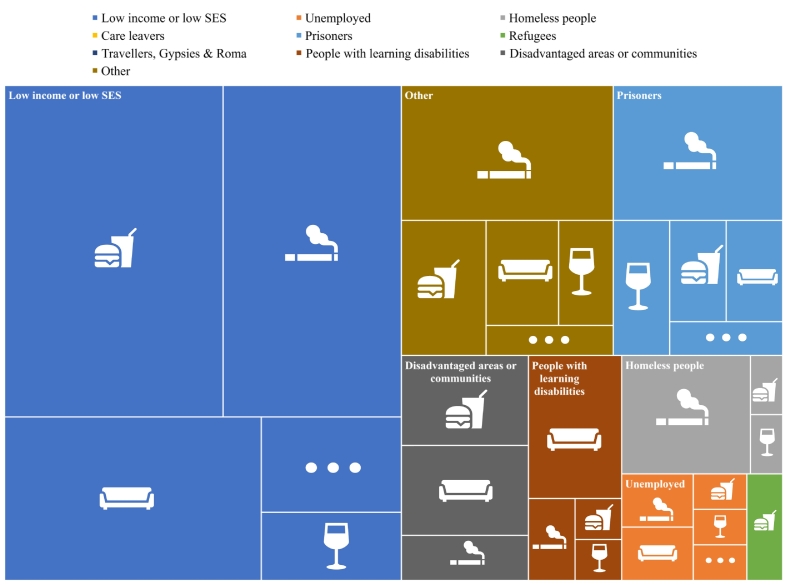
Distribution of included reviews by disadvantaged group and behaviour The three dots represent ‘other’ behaviours (outside review scope). SES Socio-economic status.

Fig. 2 (and Fig. A1) shows the distribution of included reviews targeting each behaviour by group. Some reviews included multiple groups or behaviours and appear in this chart more than once. A cross-tabulation showing the number of reviews identified for each behaviour/group combination (Table A8) and a treemap showing the distribution of reviews by behaviour (Fig. A2) can be found in the Appendix.

Most reviews (*n* = 68) focused on people with low income or SES. Thirty-eight reviews focused on low income and unhealthy diet, 31 reviews on low income and tobacco, 22 on low income and physical inactivity, and five on low income and alcohol use. Fourteen reviews included prisoners, with most of these targeting smoking alone or alongside other risk behaviours (*n* = 12). Twelve reviews included people living in disadvantaged areas or communities, mainly exploring diet (*n* = 6) or physical inactivity (n = 6). Of the ten reviews addressing homeless people, most focused on tobacco interventions or barriers and facilitators to smoking cessation (*n* = 8). Of the reviews focusing on people with learning disabilities (*n* = 9), most focused on barriers or interventions to increase physical activity (*n* = 7). We identified three reviews that focused on unemployed people and two on barriers or facilitators for refugees or asylum seekers. We found no reviews on care leavers or Gypsy, Traveller or Roma communities that met our criteria.

All of the reviews reporting differential effects of population-level interventions explored effectiveness by income or SES and four also investigated impact by area or community (von Philipsborn et al., 2019; Vargas-Garcia et al., 2015; Baker et al., 2015a) or by being homeless (Guillaumier et al., 2012).

Several reviews failed to identify relevant studies for one or more of the specified disadvantaged groups: three reviews on homeless people (Guillaumier et al., 2012; Boland et al., 2018; Ford et al., 2013), three on prisoners (Boland et al., 2018; Ford et al., 2013; Gentry et al., 2019), one review of peer health promotion in prisons (Wright et al., 2011), and one review on managed alcohol programmes for low income and homeless people (Muckle et al., 2012) (see Table 2). Search dates in these reviews were from 2010 to 2017; new primary studies may have been published since the reviews were completed. One review of sugar taxes identified only one study (Pfinder et al., 2020).

**Table 2:** Reviews that found no studies for a specified group or behaviour.

	Date of literature search	Intervention	Groups/ behaviours for which no studies were identified
Boland et al. (2018)	May 2016	Technology-based smoking cessation interventions	Homeless and prisoner populations
Ford et al. (2013)	February 2013	Smoking cessation interventions utilising peer or partner support	Homeless and prisoner populations
Gentry et al. (2019)	March 2017	*E*-cigarettes for smoking cessation/reduction, including free provision etc.	Prisoners
Guillaumier et al. (2012)	March 2012	Anti-tobacco mass media campaigns (universal or targeted)	Homeless people
Muckle et al. (2012)	March 2012	Managed alcohol programmes	Low income and homeless people
Wright et al. (2011)	September 2010	Peer education in prisons	Smoking, diet, and physical inactivity

We identified five protocols for ongoing systematic reviews: health coaching for prisoners (Almondes et al., 2017), smoking cessation for disadvantaged women (low income, unemployed or disadvantaged areas) (Burke et al., 2019), and population-level interventions aiming to explore differential effects (Vargas-Garcia et al., 2015; Baker et al., 2015b; Tully et al., 2013).

### 3.2 Other characteristics of reviews

**Fig. 3:**
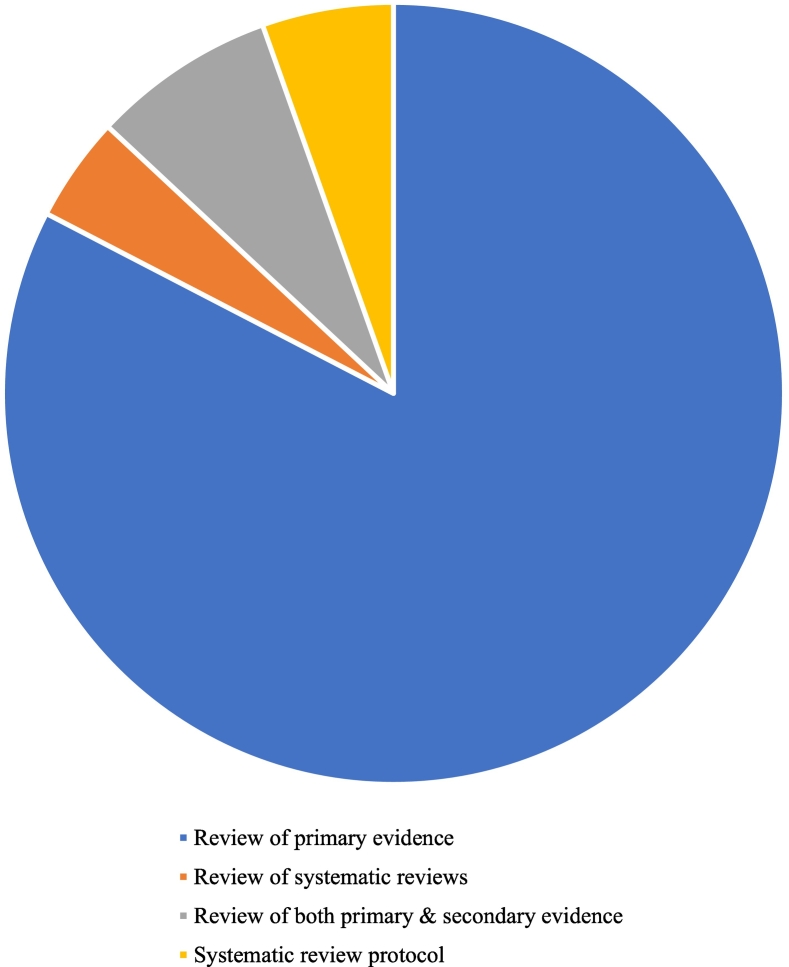
Reviews by type.

Included reviews are categorised by type in Fig. 3. The majority (83%) were systematic reviews of primary studies, and a further 8% included both primary studies and reviews. Only 4% were overviews of reviews and 5% were protocols for reviews.

**Fig. 4:**
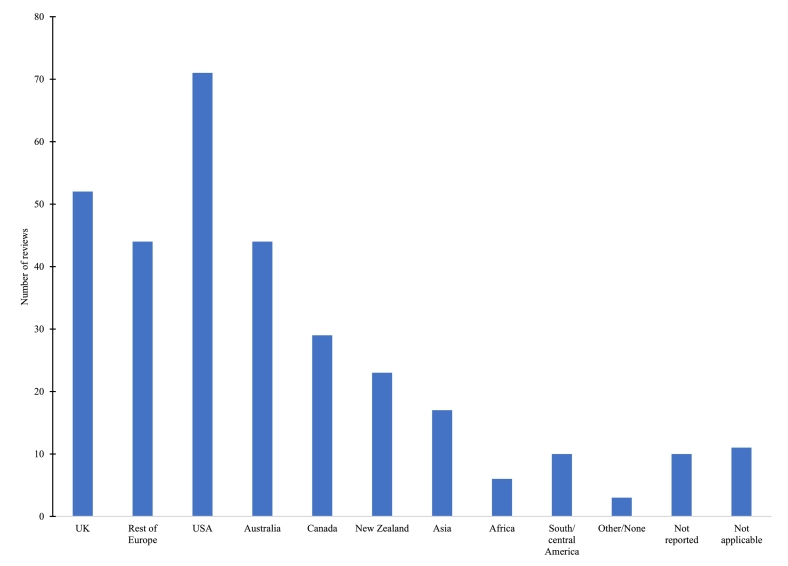
Countries of origin of primary studies included in reviews. ‘Not applicable’ includes review protocols and reviews of reviews

The number of studies included in each review ranged from 0 to 131. Fig. 4 shows the number of reviews that included at least one study from a specified country or region. Studies from the USA were included in most reviews (*n* = 71), followed by the UK (*n* = 52). Eleven reviews only included studies from the USA (Zhang et al., 2020; Stiehl et al., 2018; An et al., 2019; De Marchis et al., 2019; Verghese et al., 2019; Eicher-Miller, 2020; Engel and Ruder, 2020; Hsiao et al., 2019; Hollis-Hansen et al., 2019; Long et al., 2019; Sarink et al., 2016). This may limit the relevance of findings to other contexts, particularly as many focused on specific settings or programmes, such as food pantries, retail venues, or food supplement schemes. Two reviews focused exclusively on UK studies (Everson-Hock et al., 2013; Smith et al., 2020) and one on Australian studies (Lawlis et al., 2018). Although many reviews included interventions from any setting, some targeted specific settings such as food retail, workplaces, prisons, or food banks.

**Fig. 5:**
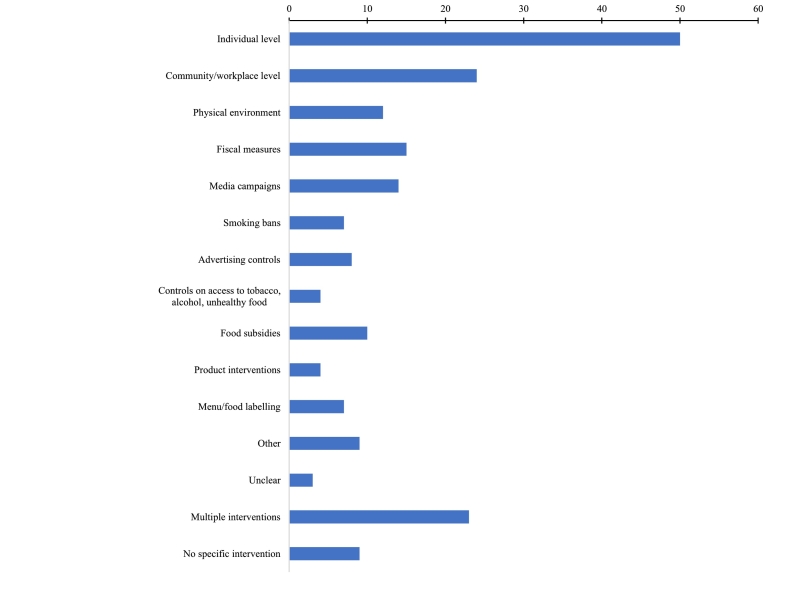
Number of reviews including each intervention type Multiple interventions: reviews including more than one intervention type

Data on intervention type are presented in Fig. 5. Fifty reviews included evidence on individual-level interventions (delivered to individuals or groups) and 24 included ‘community-level’ interventions (delivered to a whole community, or in specific settings such as workplace, prison or school). Thirty-eight reviews included policy or environmental interventions delivered on a larger scale: changes to the physical environment (e.g. new food retail, infrastructure to facilitate walking); fiscal measures (e.g. taxation); media campaigns; smoking bans; advertising controls (including promotion restrictions, plain packaging, warning labels); controls on access (e.g. age-of-sale legislation); food subsidies; product interventions or policies (e.g. reformulation to reduce salt content of food); menu or food labelling; other (e.g. food outlet award schemes, free folic acid supplements). Most policy or environmental interventions were universal but there were some reviews of targeted interventions, including food subsidies (Zhang et al., 2020; Verghese et al., 2019; Engel and Ruder, 2020; Black et al., 2012; Olstad et al., 2017; Ohly et al., 2017), targeted anti-tobacco media campaigns (Guillaumier et al., 2012), and food retail opportunities in low-income areas (Hsiao et al., 2019; Hollis-Hansen et al., 2019; Langellier et al., 2013).

**Fig. 6:**
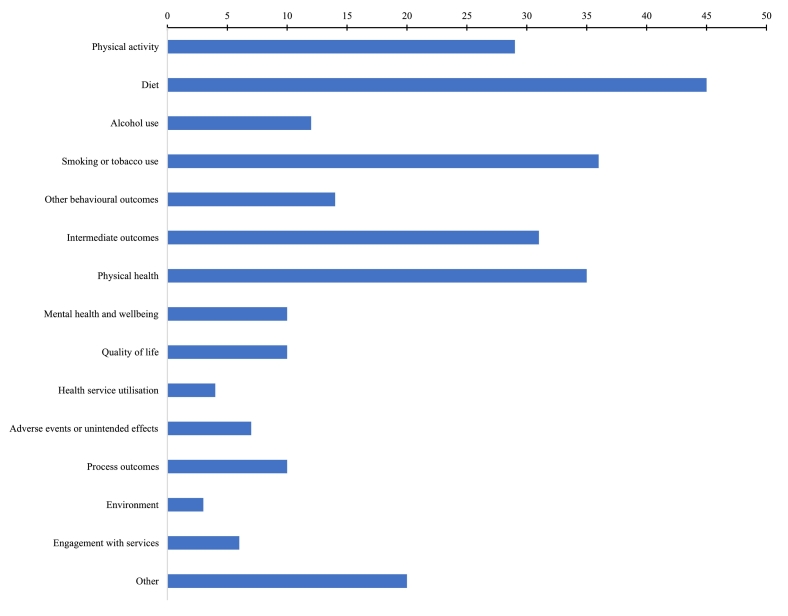
Number of reviews including each outcome type

In addition to risk behaviours, reviews reported intermediate outcomes (e.g. attitudes, knowledge), physical or mental health, quality of life, health service utilisation, engagement with services, environmental context (e.g. changes to environment, density of advertising), adverse or unintended effects, and process outcomes (e.g. recruitment, acceptability). Fig. 6 shows the number of reviews reporting at least one outcome from each category.

### 3.3 Barriers and facilitators to behaviour change

Sixteen of the 92 reviews explored perceived barriers and facilitators to changing risk behaviours. Nine reviews focused on low income groups (four on diet (Zhang et al., 2020; Everson-Hock et al., 2013; Ohly et al., 2017; Zorbas et al., 2018); two on physical inactivity (Everson-Hock et al., 2013; Rawal et al., 2020); four on smoking (Hefler and Chapman, 2015; Twyman et al., 2014; Lucherini et al., 2020; van Wijk et al., 2019)), two on disadvantaged areas (one on smoking (Hefler and Chapman, 2015); one on physical inactivity (Kramer et al., 2017)), three on prisoners (smoking (Gentry et al., 2019; Twyman et al., 2014; Puljevic and Segan, 2019)); two on homeless people (smoking (Gentry et al., 2019; Twyman et al., 2014)), two on refugees (diet (Lawlis et al., 2018; Elshahat and Moffat, 2020)), and two on people with learning disabilities (physical inactivity (Bodde and Seo, 2009; Bossink et al., 2017)). A broad range of barriers were reported, but evidence on facilitators was more limited (see Table 3 and Table A7).

**Table 3:** Barriers and facilitators to behaviour change identified by reviews.

		Bodde and Seo, (2009)	Bossink et al., (2017)	Elshahat and Moffat, (2020)	Everson-Hock et al., (2013)	Gentry et al., (2019)	Hefler and Chapman, (2015)	Kramer et al., (2017)	Lawlis et al., (2018)	Lucherini et al., (2020)	Ohly et al., (2017)	Puljevic and Segan, (2019)	Rawal et al., (2020)	Twyman et al., (2014)	van Wijk et al., (2019)	Zhang et al., (2020)	Zorbas et al., (2018)
Group	Low income / socio-economic status				✓		✓			✓	✓		✓	✓	✓	✓	✓
	Unemployed people																
	Homeless people					✓								✓			
	Prisoners					✓						✓		✓			
	Refugees or asylum seekers			✓					✓								
	People with learning disabilities	✓	✓														
	Disadvantaged areas or communities						✓	✓									
Behaviour	Tobacco use					✓	✓			✓		✓		✓	✓		
	Excessive alcohol use																
	Physical inactivity	✓	✓		✓			✓					✓				
	Unhealthy diet			✓	✓				✓		✓					✓	✓
Barriers	Lack of social/family support	✓	✓	✓	✓		✓					✓	✓	✓	✓		✓
	Social environment/ cultural norms		✓	✓	✓	✓	✓	✓	✓	✓	✓			✓	✓		✓
	Physical environment	✓	✓	✓	✓		✓	✓	✓			✓	✓		✓		✓
	Weather	✓	✓		✓												✓
	Transport issues	✓	✓	✓					✓				✓		✓		✓
	Limited availability of healthy foods			✓					✓								
	Health & disability		✓										✓				
	Mental health issues				✓	✓	✓			✓		✓		✓			✓
	Addiction					✓								✓			✓
	Financial constraints	✓	✓	✓	✓	✓			✓	✓	✓		✓	✓	✓	✓	✓
	Risk/safety	✓	✓		✓	✓		✓					✓	✓			✓
	Lack of opportunities/resources	✓	✓						✓				✓	✓	✓		
	Issues with services	✓	✓										✓		✓	✓	
	Adverse effects of behaviour change					✓								✓	✓		
	Motivation		✓											✓	✓		✓
	Attitudes			✓	✓		✓			✓			✓	✓	✓		✓
	Lack of knowledge/understanding			✓	✓					✓					✓	✓	✓
	Lack of skills		✓						✓				✓				✓
	Lack of confidence/self- efficacy			✓	✓								✓	✓	✓		✓
	Living/ working circumstances				✓					✓				✓	✓		
	Habit/ routine		✓		✓									✓	✓		✓
	Time constraints			✓	✓					✓			✓			✓	✓
	Competing needs										✓			✓	✓		
	Other		✓	✓	✓		✓		✓	✓				✓	✓		✓
Facilitators	Family/social support		✓	✓	✓	✓		✓					✓				✓
	Social environment		✓	✓		✓										✓	✓
	Facilitators related to services		✓													✓	
	Available opportunities		✓	✓									✓			✓	
	Physical environment		✓	✓		✓											✓
	Weather		✓														
	Motivation		✓		✓												✓
	Attitudes			✓	✓	✓				✓		✓					✓
	Skills/ability		✓	✓													✓
	Confidence/ self-efficacy				✓	✓										✓	✓
	Knowledge/ information			✓	✓					✓						✓	✓
	Health (as motivating factor)											✓					✓
	Financial support			✓													
	Other		✓	✓		✓	✓			✓		✓					✓

## 4 Discussion

To the authors' knowledge, this is the first review to map systematic reviews of interventions to reduce major risk behaviours (smoking, unhealthy diet, physical inactivity, alcohol use) in disadvantaged groups and communities. Ninety-two reviews were identified, covering different combinations of groups and behaviours. Despite the large number of reviews, we identified gaps in the evidence base. We found no systematic reviews on care leavers or Gypsies, Traveller and Roma communities. Very few reviews focus specifically on refugees or asylum seekers or unemployed people. The evidence relating to individual behaviours varies between groups and gaps were identified; for example, the evidence on homeless people relates mainly to tobacco use and the evidence on people with learning disabilities mainly to physical inactivity. These evidence gaps may limit efforts to tackle risk behaviours in specific groups. Although there is overlap between low income and other forms of disadvantage, groups such as Gypsies, Travellers, and Roma and refugees and asylum seekers are likely to have very specific needs which require tailored approaches and interventions.

The evidence gaps identified suggest a need for new reviews. However, barriers to conducting research with disadvantaged groups are well documented, including issues with sampling, recruitment, data collection, intervention uptake, fidelity, and retention of participants (Bonevski et al., 2014). Therefore, it is possible that the gaps identified in review level evidence mirror gaps in primary research and this is especially likely for Gypsy, Traveller and Roma populations (Condon et al., 2019). In some groups, risk behaviours may be particularly difficult to address. Prisoners, for example, have limited control over the food provided by the prison canteen, and few opportunities for physical activity when confined to their cells for 23 out of 24 h a day (Meek, 2018).

Risk behaviours may not be seen as a priority in some disadvantaged groups, possibly due to the wide range of poor health outcomes that many of these groups face (Aldridge et al., 2018; Peters et al., 2009; Fazel and Baillargeon, 2011). For example, evidence on the prevalence of risk behaviours in refugees and asylum seekers and care leavers is limited. A 2016 systematic review found few studies assessing the prevalence of harmful or hazardous alcohol use in refugees and asylum seekers, but based on the available evidence estimates ranged from 17 to 36% in camps and 4–7% in community settings (Horyniak et al., 2016). Similarly, evidence on care leavers appears limited, but a few studies have reported increased tobacco use and alcohol abuse in those leaving foster homes (Gypen et al., 2017; Braciszewski and Stout, 2012).

Low levels of exercise have been reported in people with learning disabilities (Dairo et al., 2016). We identified two systematic reviews highlighting barriers to engaging in physical activity (Bodde and Seo, 2009; Bossink et al., 2017). More limited evidence suggests that tobacco use and excessive alcohol use have been found in people with learning disabilities (mean prevalence of 18% and 22% respectively (Huxley et al., 2019)) and that this group has very specific health promotion needs in relation to alcohol and tobacco use (Kerr et al., 2017).

Systematic reviews focusing on homeless people have mostly targeted tobacco use (although many reviews found no eligible studies). There may be logistical challenges in designing and delivering health interventions for this group (Ojo-Fati et al., 2017). It is also possible that the four risk behaviours that were the focus in our scoping review are not viewed as priorities for intervention given the high rates of infectious diseases, substance misuse, injuries, and psychiatric disorders reported for homeless people (Fazel et al., 2014).

The evidence on the effectiveness of population-level interventions almost exclusively relates to people with low income or SES. This suggests that little is known about how effective these interventions might be in reducing risk behaviours for many other disadvantaged groups. There is however a body of theoretical work outlining the kinds of interventions that are likely to have the greatest impact (Dahlgren and Whitehead, 2007; Whitehead and Dahlgren, 2007). These include structural interventions that change the environments in which people make lifestyle choices, such as fiscal policies, legislation to restrict access to unhealthy products, advertising bans and subsidies for healthy food (Dahlgren and Whitehead, 2007).

In terms of specific risk behaviours, we found little evidence on reducing alcohol use. In part this may reflect our inclusion criteria, as reviews of substance use interventions that did not focus on alcohol were excluded. Reviews of alcohol control policies that were solely based on modelling studies and reviews that reported only health outcomes (e.g. alcohol-related harm) as opposed to reductions in harmful drinking were also ineligible.

Overall, we found few reviews of qualitative studies exploring the views and perspectives of disadvantaged people with regard to changing risk behaviours. This means we know little about the barriers and facilitators in specific groups (e.g. in refugees and asylum seekers) and for individual risk behaviours. These gaps could act as an obstacle to designing and implementing effective programmes.

We identified a number of reviews that reported finding no eligible primary studies. These ‘empty’ reviews, however, tended to explore very specific approaches which might not be the most appropriate way of reaching particular population groups (e.g. mass media campaigns for homeless people) and therefore do not necessarily reflect important evidence gaps.

The COVID-19 pandemic has further highlighted the importance of addressing health inequalities. The situation has been described as a ‘syndemic’ for disadvantaged groups, with the pandemic interacting with pre-existing inequalities in NCDs and the social determinants of health (Bambra et al., 2020). The COVID-19 Marmot Review highlighted that containment measures in the UK may have led to worsening inequalities in risk behaviours (Marmot et al., 2020) and the World Health Organization has stressed the importance of addressing NCDs as part of response and recovery (World Health Organization, United Nations Development Programme, 2020). Although the UK government has published a new obesity strategy that calls on people to embrace a healthier lifestyle (Department of Health and Social Care, 2020), health inequalities are expected to worsen without the introduction of policies to protect disadvantaged populations from the adverse consequences of the pandemic (Whitehead et al., 2021).

The strengths of this scoping review include a comprehensive search, robust methods, and an inclusive approach to defining systematic reviews to ensure that relevant and potentially useful literature was not excluded. The review maps a wide body of literature, covering different disadvantaged groups and four key risk behaviours, which to our knowledge has not been brought together before. We included reviews addressing barriers and facilitators to behaviour change as this evidence is crucial in planning interventions and formulating policies.

Limitations include a lack of a widely-accepted definition of disadvantaged groups (Ford et al., 2019) which means that some groups will not have been captured in our review. While we focused on four highly prevalent and important risk behaviours, we are aware that individuals are likely to engage in multiple risky behaviours, some of which are outside the scope of our review (Meader et al., 2016). Reviews of population-level interventions were included to capture evidence on interventions aimed at creating a more enabling environment for behaviour change. However, we acknowledge that not all population-level interventions aim to do this (e.g. media campaigns) and reviews of smaller-scale interventions that involved environmental change (e.g. changing the layout of a specific shop) were not included. As this was a scoping review we did not assess the quality of the reviews or classify and synthesise interventions by type (theory of change).

## 5 Conclusion

This scoping review has identified a large number of systematic reviews addressing four key risk behaviours in disadvantaged groups. We also found gaps in the evidence base where new systematic reviews could make a useful contribution. This includes systematic reviews of Gypsy, Travellerand Roma communities, care leavers, and refugees and asylum seekers. There is a need to identify the major barriers faced by these groups as well as the challenges faced by those trying to develop appropriate interventions. An in-depth review of qualitative studies and grey literature might identify accounts of failed attempts to develop or implement interventions that could inform the development and piloting of new approaches.

We identified few reviews on alcohol use in any disadvantaged group, suggesting a need for reviews of both interventions and barriers and facilitators to change. An overview of systematic reviews addressing smoking, diet, and physical inactivity in low income or SES populations could make a useful contribution by assessing the quality of the evidence base, and highlighting robust findings from higher quality reviews. Importantly, interventions could be categorised according to their theory of change and the level at which they are expected to operate which would facilitate the identification of effective intervention types. A qualitative overview of reviews on the views of disadvantaged groups about behaviour change would be useful, allowing common barriers across groups to be identified as well as factors that are unique to specific groups.

## Author contributions

AS and MW conceived the study. All authors contributed to the design of the review. KW conducted the searches. ES, MR and AS screened the studies. MR and ES extracted and mapped the data. MR created the figures. All authors contributed to the interpretation of findings. ES drafted the manuscript and all authors contributed to revising it. All authors approved the manuscript and accept responsibility to submit for publication.

## Funding

This publication is based on independent research carried out by the Public Health Policy Research Unit (PH-PRU), commissioned and funded by the National Institute for Health Research Policy Research Programme (UK). The views expressed in this publication are those of the authors and not necessarily those of the NHS, the National Institute for Health Research, the Department of Health and Social Care or its arm's length bodies, and other UK Government Departments. The funder of the study had no role in study design, data collection, analysis or interpretation, writing of the report or decision to publish.

## Declaration of competing interest

The authors have no conflicts of interest to disclose.

## Appendix A Supplementary data

Supplementary material Image 1

## References

[bb0005] Aldridge R.W., Story A., Hwang S.W., Nordentoft M., Luchenski S.A., Hartwell G. (2018). Morbidity and mortality in homeless individuals, prisoners, sex workers, and individuals with substance use disorders in high-income countries: a systematic review and meta-analysis. Lancet.

[bb0010] Almondes N., Downie D., Cinar A.B., Richards D., Freeman R. (2017). Is health coaching effective in changing the health status and behaviour of prisoners?-a systematic review protocol. Syst. Rev..

[bb0015] An R., Wang J., Liu J., Shen J., Loehmer E., McCaffrey J. (2019). A systematic review of food pantry-based interventions in the USA. Public Health Nutr..

[bb0020] Arksey H., O’Malley L. (2005). Scoping studies: towards a methodological framework. Int. J. Soc. Res. Methodol..

[bb0025] Baggett T.P., Rigotti N.A. (2010). Cigarette smoking and advice to quit in a National Sample of homeless adults. Am. J. Prev. Med..

[bb0030] Baker P.R., Francis D.P., Soares J., Weightman A.L., Foster C. (2015). Community wide interventions for increasing physical activity. Cochrane Database Syst. Rev..

[bb0035] Baker P.R.A., Dobbins M., Soares J., Francis D.P., Weightman A.L., Costello J.T. (2015). Public health interventions for increasing physical activity in children, adolescents and adults: An overview of systematic reviews. Cochrane Database Syst. Rev..

[bb0040] Bambra C., Riordan R., Ford J., Matthews F. (2020). The COVID-19 pandemic and health inequalities. J. Epidemiol. Community Health.

[bb0045] Bankiewicz U., Robinson C. (2020). https://digital.nhs.uk/data-and-information/publications/statistical/health-survey-for-england/2019.

[bb0050] Black A.P., Brimblecombe J., Eyles H., Morris P., Vally H., K OD. (2012). Food subsidy programs and the health and nutritional status of disadvantaged families in high income countries: a systematic review. BMC Public Health.

[bb0055] Bodde A.E., Seo D.C. (2009). A review of social and environmental barriers to physical activity for adults with intellectual disabilities. Disabil Health J.

[bb0060] Boland V.C., Stockings E.A., Mattick R.P., McRobbie H., Brown J., Courtney R.J. (2018). The methodological quality and effectiveness of technology-based smoking cessation interventions for disadvantaged groups: a systematic review and meta-analysis. Nicotine Tob. Res..

[bb0065] Bonevski B., Randell M., Paul C., Chapman K., Twyman L., Bryant J. (2014). Reaching the hard-to-reach: a systematic review of strategies for improving health and medical research with socially disadvantaged groups. BMC Med. Res. Methodol..

[bb0070] Bossink L.W.M., van der Putten A.A., Vlaskamp C. (2017). Understanding low levels of physical activity in people with intellectual disabilities: a systematic review to identify barriers and facilitators. Res. Dev. Disabil..

[bb0075] Braciszewski J.M., Stout R.L. (2012). Substance use among current and former foster youth: a systematic review. Child Youth Serv. Rev..

[bb0080] Burke E., Dobbie F., Dougall N., Adebolu Oluwaseun M., Mockler D., Vance J. (2019). Smoking cessation programmes for women living in disadvantaged communities, “we can quit 2”: a systematic review protocol. HRB Open Res.

[bb0085] Cabinet Office and Department of Health & Social Care (2019). Advancing our Health: Prevention in the 2020s. https://www.gov.uk/government/consultations/advancing-our-health-prevention-in-the-2020s.

[bb0090] Christensen H.N., Diderichsen F., Hvidtfeldt U.A., Lange T., Andersen P.K., Osler M. (2017). Joint effect of alcohol consumption and educational level on alcohol-related medical events: a Danish register-based cohort study. Epidemiology.

[bb0095] Colquhoun H.L., Levac D., O’Brien K.K., Straus S., Tricco A.C., Perrier L. (2014). Scoping reviews: time for clarity in definition, methods, and reporting. J. Clin. Epidemiol..

[bb0100] Condon L., Bedford H., Ireland L., Kerr S., Mytton J., Richardson Z. (2019). Engaging Gypsy, Roma, and Traveller communities in research: maximizing opportunities and overcoming challenges. Qual. Health Res..

[bb0105] Cook B., Wayne G.F., Valentine A., Lessios A., Yeh E. (2013). Revisiting the evidence on health and health care disparities among the Roma: a systematic review 2003–2012. Int J Public Health.

[bb0110] Dahlgren G., Whitehead M. (2007). https://www.euro.who.int/__data/assets/pdf_file/0018/103824/E89384.pdf.

[bb0115] Dairo Y.M., Collett J., Dawes H., Oskrochi G.R. (2016). Physical activity levels in adults with intellectual disabilities: a systematic review. Prev. Med. Rep..

[bb0120] De Marchis E.H., Torres J.M., Benesch T., Fichtenberg C., Allen I.E., Whitaker E.M. (2019). Interventions addressing food insecurity in health care settings: a systematic review. Ann. Fam. Med..

[bb0125] Department of Health and Social Care (2020). Tackling Obesity: Empowering Adults and Children to Live Healthier Lives. https://www.gov.uk/government/publications/tackling-obesity-government-strategy.

[bb0130] Eicher-Miller H.A. (2020). A review of the food security, diet and health outcomes of food pantry clients and the potential for their improvement through food pantry interventions in the United States. Physiol. Behav..

[bb0135] Elshahat S., Moffat T. (2020). Dietary practices among Arabic-speaking immigrants and refugees in Western societies: a scoping review. Appetite.

[bb0140] Engel K., Ruder E.H. (2020). Fruit and vegetable incentive programs for supplemental nutrition assistance program (SNAP) participants: a scoping review of program structure. Nutrients.

[bb0145] Equality and Human Rights Commission (2016). England's Most Disadvantaged Groups: Homeless People. https://www.equalityhumanrights.com/sites/default/files/is-england-fairer-2016-most-disadvantaged-groups-homeless-people.pdf.

[bb0150] Equality and Human Rights Commission (2016). England's Most Disadvantaged Groups: Gypsies, Travellers and Roma. https://www.equalityhumanrights.com/sites/default/files/is-england-fairer-2016-most-disadvantaged-groups-gypsies-travellers-roma.pdf.

[bb0155] Equality and Human Rights Commission (2016). England's Most Disadvantaged Groups: Migrants, Refugees and Asylum Seekers. https://www.equalityhumanrights.com/sites/default/files/is-england-fairer-2016-most-disadvantaged-groups-migrants-refugees-asylum-seekers.pdf.

[bb0160] Equality and Human Rights Commission (2016). England's Most Disadvantaged Groups: People with Learning Disabilities. https://www.equalityhumanrights.com/sites/default/files/is-england-fairer-2016-most-disadvantaged-groups-learning-disabilities.pdf.

[bb0165] Everson-Hock E.S., Johnson M., Jones R., Woods H.B., Goyder E., Payne N. (2013). Community-based dietary and physical activity interventions in low socioeconomic groups in the UK: a mixed methods systematic review. Prev. Med..

[bb0170] Fazel S., Baillargeon J. (2011). The health of prisoners. Lancet.

[bb0175] Fazel S., Geddes J.R., Kushel M. (2014). The health of homeless people in high-income countries: descriptive epidemiology, health consequences, and clinical and policy recommendations. Lancet.

[bb0180] Ford P., Clifford A., Gussy K., Gartner C. (2013). A systematic review of peer-support programs for smoking cessation in disadvantaged groups. Int. J. Environ. Res. Public Health.

[bb0185] Ford J., Sim F., Mackie P. (2019). Health inequalities: the need for clarity in the confusion. Public Health.

[bb0190] Freyer-Adam J., Gaertner B., Tobschall S., John U. (2011). Health risk factors and self-rated health among job-seekers. BMC Public Health.

[bb0195] Gentry S., Forouhi N.G., Notley C. (2019). Are electronic cigarettes an effective aid to smoking cessation or reduction among vulnerable groups? A systematic review of quantitative and qualitative evidence. Nicotine Tob. Res..

[bb0200] Guillaumier A., Bonevski B., Paul C. (2012). Anti-tobacco mass media and socially disadvantaged groups: a systematic and methodological review. Drug Alcohol Rev.

[bb0205] Gypen L., Vanderfaeillie J., De Maeyer S., Belenger L., Van Holen F. (2017). Outcomes of children who grew up in foster care: systematic-review. Child Youth Serv. Rev..

[bb0210] Hefler M., Chapman S. (2015). Disadvantaged youth and smoking in mature tobacco control contexts: a systematic review and synthesis of qualitative research. Tob. Control..

[bb0215] Henkel D. (2011). Unemployment and substance use: a review of the literature (1990-2010). Curr Drug Abuse Rev.

[bb0220] Herbert K., Plugge E., Foster C., Doll H. (2012). Prevalence of risk factors for non-communicable diseases in prison populations worldwide: a systematic review. Lancet.

[bb0225] HM Government (2016). Keep On Caring: Supporting Young People from Care to Independence. https://www.gov.uk/government/publications/keep-on-caring-supporting-young-people-from-care-to-independence.

[bb0230] Hollis-Hansen K., Vermont L., Zafron M.L., Seidman J., Leone L. (2019). The introduction of new food retail opportunities in lower-income communities and the impact on fruit and vegetable intake: a systematic review. Transl. Behav. Med..

[bb0235] Horyniak D., Melo J.S., Farrell R.M., Ojeda V.D., Strathdee S.A. (2016). Epidemiology of substance use among forced migrants: a global systematic review. PLoS One.

[bb0240] Hsiao B.S., Sibeko L., Troy L.M. (2019). A systematic review of mobile produce markets: facilitators and barriers to use, and associations with reported fruit and vegetable intake. J. Acad. Nutr. Diet..

[bb0245] Huxley A., Dalton M., Tsui Y.Y.Y., Hayhurst K.P. (2019). Prevalence of alcohol, smoking, and illicit drug use amongst people with intellectual disabilities: review. Drugs: Educat. Prevent. Pol..

[bb0255] Katikireddi S.V., Whitley E., Lewsey J., Gray L., Leyland A.H. (2017). Socioeconomic status as an effect modifier of alcohol consumption and harm: analysis of linked cohort data. Lancet Public Health.

[bb0260] Kerr S., Lawrence M., Middleton A.R., Fitzsimmons L., Darbyshire C. (2017). Tobacco and alcohol use in people with mild/moderate intellectual disabilities: giving voice to their health promotion needs. J. Appl. Res. Intellect. Disabil..

[bb0265] Kramer D., Lakerveld J., Stronks K., Kunst A.E. (2017). Uncovering how urban regeneration programs may stimulate leisure-time walking among adults in deprived areas: a realist review. Int. J. Health Serv..

[bb0270] Langellier B.A., Garza J.R., Prelip M.L., Glik D., Brookmeyer R., Ortega A.N. (2013). Corner store inventories, purchases, and strategies for intervention: a review of the literature. Calif J Health Promot.

[bb0275] Lawlis T., Islam W., Upton P. (2018). Achieving the four dimensions of food security for resettled refugees in Australia: a systematic review. Nutr. Diet..

[bb0280] Levac D., Colquhoun H., O’Brien K.K. (2010). Scoping studies: advancing the methodology. Implement. Sci..

[bb0285] Long C.R., Rowland B., Steelman S.C., McElfish P.A. (2019). Outcomes of disease prevention and management interventions in food pantries and food banks: a scoping review. BMJ Open.

[bb0290] Lucherini M., Hill S., Smith K. (2020). Inequalities, harm reduction and non-combustible nicotine products: a meta-ethnography of qualitative evidence. BMC Public Health.

[bb0250] Marmot M., Allen J., Goldblatt P., Herd E., Morrison J. (2020). https://www.instituteofhealthequity.org/resources-reports/build-back-fairer-the-covid-19-marmot-review.

[bb0295] Marteau T.M., Rutter H., Marmot M. (2021). Changing behaviour: an essential component of tackling health inequalities. BMJ.

[bb0300] McKee-Ryan F., Song Z., Wanberg C.R., Kinicki A.J. (2005). Psychological and physical well-being during unemployment: a meta-analytic study. J Appl Psychol.

[bb0305] Meader N., King K., Moe-Byrne T., Wright K., Graham H., Petticrew M. (2016). A systematic review on the clustering and co-occurrence of multiple risk behaviours. BMC Public Health.

[bb0310] Meek R. (2018). https://assets.publishing.service.gov.uk/government/uploads/system/uploads/attachment_data/file/733184/a-sporting-chance-an-independent-review-sport-in-justice.pdf.

[bb0315] Muckle W., Muckle J., Welch V., Tugwell P. (2012). Managed alcohol as a harm reduction intervention for alcohol addiction in populations at high risk for substance abuse. Cochrane Database Syst. Rev..

[bb0320] Murray C.J.L., Aravkin A.Y., Zheng P., Abbafati C., Abbas K.M., Abbasi-Kangevari M. (2020). Global burden of 87 risk factors in 204 countries and territories, 1990–2019: a systematic analysis for the global burden of disease study 2019. Lancet.

[bb0325] National Audit Office (2015). Care Leavers' Transition to Adulthood. https://www.nao.org.uk/report/care-leavers-transitions-to-adulthood/.

[bb0330] National Institute for Health Research (2020). Better health and Care for All: Health and Care Services for People with Learning Disabilities. https://evidence.nihr.ac.uk/themedreview/better-health-and-care-for-all/.

[bb0335] NHS (2019). The NHS Long Term Plan. https://www.longtermplan.nhs.uk/publication/nhs-long-term-plan/.

[bb0340] NHS Digital (2019). Health Survey for England 2018: Adult's Health-Related Behaviours. https://digital.nhs.uk/data-and-information/publications/statistical/health-survey-for-england/2018.

[bb0345] NHS Digital (2020). Statistics on Obesity, Physical Activity and Diet, England, 2020. https://digital.nhs.uk/data-and-information/publications/statistical/statistics-on-obesity-physical-activity-and-diet/england-2020.

[bb0350] Norström F., Virtanen P., Hammarström A., Gustafsson P.E., Janlert U. (2014). How does unemployment affect self-assessed health? A systematic review focusing on subgroup effects. BMC Public Health.

[bb0355] Norström F., Waenerlund A.-K., Lindholm L., Nygren R., Sahlén K.-G., Brydsten A. (2019). Does unemployment contribute to poorer health-related quality of life among Swedish adults?. BMC Public Health.

[bb0360] Ohly H., Crossland N., Dykes F., Lowe N., Hall-Moran V. (2017). A realist review to explore how low-income pregnant women use food vouchers from the UK’s healthy start programme. BMJ Open.

[bb0365] Ojo-Fati O., Joseph A.M., Ig-Izevbekhai J., Thomas J.L., Everson-Rose S.A., Pratt R. (2017). Practical issues regarding implementing a randomized clinical trial in a homeless population: strategies and lessons learned. Trials.

[bb0370] Olstad D.L., Ancilotto R., Teychenne M., Minaker L.M., Taber D.R., Raine K.D. (2017). Can targeted policies reduce obesity and improve obesity-related behaviours in socioeconomically disadvantaged populations? A systematic review. Obes. Rev..

[bb0375] Osborne B., Cooper V. (2018). https://digital.nhs.uk/data-and-information/publications/statistical/health-survey-for-england/2017.

[bb0380] Peters J., Parry G.D., Van Cleemput P., Moore J., Cooper C.L., Walters S.J. (2009). Health and use of health services: a comparison between Gypsies and Travellers and other ethnic groups. Ethn. Health.

[bb0385] Pfinder M., Heise T.L., Hilton Boon M., Pega F., Fenton C., Griebler U. (2020). Taxation of unprocessed sugar or sugar-added foods for reducing their consumption and preventing obesity or other adverse health outcomes. Cochrane Database Syst. Rev..

[bb0390] Public Health England (2015). Reducing Smoking in Prisons: Management of Tobacco Use and Nicotine Withdrawal. https://www.gov.uk/government/publications/smoking-in-prisons-management-of-tobacco-use-and-nicotine-withdrawal.

[bb0395] Public Health England (2019). Health Profile for England 2019: 9 Key Points from our 2019 Update. https://publichealthengland.exposure.co/health-profile-for-england-2019.

[bb0400] Public Health England (2019). PHE Strategy 2020–25. https://www.gov.uk/government/publications/phe-strategy-2020-to-2025.

[bb0405] Puljevic C., Segan C.J. (2019). Systematic review of factors influencing smoking following release from smoke-free prisons. Nicotine Tob. Res..

[bb0410] Rawal L.B., Smith B.J., Quach H., Renzaho A.M.N. (2020). Physical activity among adults with low socioeconomic status living in industrialized countries: a meta-ethnographic approach to understanding socioecological complexities. J. Environ. Public Health.

[bb0415] Sarink D., Peeters A., Freak-Poli R., Beauchamp A., Woods J., Ball K. (2016). The impact of menu energy labelling across socioeconomic groups: a systematic review. Appetite.

[bb0420] Smith C.E., Hill S.E., Amos A. (2020). Impact of specialist and primary care stop smoking support on socio-economic inequalities in cessation in the United Kingdom: a systematic review and national equity analysis. Addiction.

[bb0425] Steel N., Ford J.A., Newton J.N., Davis A.C.J., Vos T., Naghavi M. (2018). Changes in health in the countries of the UK and 150 English local authority areas 1990–2016: a systematic analysis for the global burden of disease study 2016. Lancet.

[bb0430] Stiehl E., Shivaprakash N., Thatcher E., Ornelas I.J., Kneipp S., Baron S.L. (2018). Worksite health promotion for low-wage workers: a scoping literature review. Am. J. Health Promot..

[bb0435] The King's Fund (2019). Advancing our Health: Prevention in the 2020s Consultation – The King's Fund Response. https://www.kingsfund.org.uk/publications/advancing-health-prevention-consultation.

[bb0440] The Lancet (2020). https://www.thelancet.com/pb-assets/Lancet/gbd/summaries/risks/dietary-risks.pdf.

[bb0445] The Lancet (2020). https://www.thelancet.com/pb-assets/Lancet/gbd/summaries/risks/alcohol-use.pdf.

[bb0450] The Lancet (2020). https://www.thelancet.com/pb-assets/Lancet/gbd/summaries/risks/low-physical-activity.pdf.

[bb0455] Tricco A.C., Lillie E., Zarin W., O’Brien K.K., Colquhoun H., Levac D. (2018). PRISMA extension for scoping reviews (PRISMA-ScR): checklist and explanation. Ann. Intern. Med..

[bb0460] Tully M.A., Kee F., Foster C., Cardwell C.R., Weightman A.L., Cupples M.E. (2013). Built environment interventions for increasing physical activity in adults and children. Cochrane Database Syst. Rev..

[bb0465] Twyman L., Bonevski B., Paul C., Bryant J. (2014). Perceived barriers to smoking cessation in selected vulnerable groups: a systematic review of the qualitative and quantitative literature. BMJ Open.

[bb0470] van Wijk E.C., Landais L.L., Harting J. (2019). Understanding the multitude of barriers that prevent smokers in lower socioeconomic groups from accessing smoking cessation support: a literature review. Prev. Med..

[bb0475] Vargas-Garcia E.J., El Evans C., Cade J.E. (2015). Impact of interventions to reduce sugar-sweetened beverage intake in children and adults: a protocol for a systematic review and meta-analysis. Syst. Rev..

[bb0480] Verghese A., Raber M., Sharma S. (2019). Interventions targeting diet quality of supplemental nutrition assistance program (SNAP) participants: a scoping review. Prev. Med..

[bb0485] von Philipsborn P., Stratil J.M., Burns J., Busert L.K., Pfadenhauer L.M., Polus S. (2019). Environmental interventions to reduce the consumption of sugar-sweetened beverages and their effects on health. Cochrane Database Syst. Rev..

[bb0490] Whitehead M., Dahlgren G. (2007). https://www.euro.who.int/__data/assets/pdf_file/0010/74737/E89383.pdf.

[bb0495] Whitehead M., Taylor-Robinson D., Barr B. (2021). Poverty, health, and covid-19. BMJ.

[bb0500] World Health Organization (2018). Noncommunicable diseases (Factsheet). https://www.who.int/en/news-room/fact-sheets/detail/noncommunicable-diseases.

[bb0505] World Health Organization (2020). WHO reveals leading causes of death and disability worldwide: 2000-2019. https://www.who.int/news/item/09-12-2020-who-reveals-leading-causes-of-death-and-disability-worldwide-2000-2019.

[bb0510] World Health Organization, United Nations Development Programme (2020). Responding to Noncommunicable Diseases during and Beyond the COVID-19 Pandemic. Geneva. https://www.who.int/publications/i/item/WHO-2019-nCoV-Non-communicable_diseases-Policy_brief-2020.1.

[bb0515] Wright N., Bleakley A., Butt C., Chadwick O., Mahmood K., Patel K. (2011). Peer health promotion in prisons: a systematic review. Int. J. Prison. Health.

[bb0520] Zhang Q., Alsuliman M.A., Wright M., Wang Y., Cheng X. (2020). Fruit and vegetable purchases and consumption among WIC participants after the 2009 WIC food package revision: a systematic review. Adv. Nutr..

[bb0525] Zorbas C., Palermo C., Chung A., Iguacel I., Peeters A., Bennett R. (2018). Factors perceived to influence healthy eating: a systematic review and meta-ethnographic synthesis of the literature. Nutr. Rev..

