# Supplementary material for: Reducing lifestyle risk behaviours in disadvantaged groups in high-income countries: A scoping review of systematic reviews
Source: Prev Med. 2022 Jan;154:106916. doi: 10.1016/j.ypmed.2021.106916 (PMC8803546; doi:10.1016/j.ypmed.2021.106916)
Supplement: Supplementary file 1 — Supplementary material [file mmc1.docx]

**Appendix**

**Appendix 1: Search strategies**

**1.1 MEDLINE strategy 1**

MeSH terms used for disadvantaged groups were restricted to Major focus.

Database: Ovid MEDLINE(R) ALL <1946 to January 13, 2020>

Search Strategy:

--------------------------------------------------------------------------------

1 *Poverty/ (15821)

2 *Vulnerable Populations/ (4505)

3 *Socioeconomic Factors/ (12289)

4 *Minority Health/ (407)

5 *Health Status Disparities/ (9557)

6 ((health or social$ or socioeconomic$ or socio-economic$ or racial$) adj2 (disadvantage$ or disparit$ or inequalit$)).ti,bt,ab. (35561)

7 (("at risk" or deprived or disadvantaged or disengaged or excluded or forgotten or "hard to reach" or hidden or "high risk" or inaccessible or inequitable or isolated or "low SES" or "low socioeconomic status" or marginal$ or neglected or transient or underserved or unengaged or vulnerable) adj2 (area$ or background$ or communit$ or demographic$ or group$ or inner cit$ or neighbourhood$ or neighborhood$ or population$ or rural or urban)).ti,bt,ab. (118747)

8 (("at risk" or deprived or disadvantaged or disengaged or excluded or forgotten or "hard to reach" or hidden or "high risk" or inequitable or isolated or "low SES" or "low socioeconomic status" or marginal$ or neglected or transient or underserved or unengaged or vulnerable) adj2 (adult$ or group$ or man or men or people or person$ or women or youth)).ti,bt,ab. (86991)

9 ((low level$ adj2 education) or low education level$ or low educational attainment or (low attainment adj2 education)).ti,bt,ab. (3161)

10 1 or 2 or 3 or 4 or 5 or 6 or 7 or 8 or 9 (219778)

11 *Poverty Areas/ (2584)

12 *Public housing/ (912)

13 *Residence Characteristics/ (13197)

14 (slum or slums or tenement$).ti,bt,ab. (3105)

15 tower block$.ti,bt,ab. (13)

16 (high-rise adj2 (block$ or building$)).ti,bt,ab. (216)

17 (housing adj2 (council or insecur$ or instability or poor or short-term or "short term" or social or temporary or unstable)).ti,bt,ab. (2232)

18 (house adj2 (council or insecur$ or instability or poor or short-term or "short term" or social or temporary or unstable)).ti,bt,ab. (113)

19 (houses adj2 (council or insecur$ or instability or poor or short-term or "short term" or social or temporary or unstable)).ti,bt,ab. (75)

20 (housing adj2 ("at risk" or deprived or disadvantage$ or "high risk" or inequitable or marginal$ or poverty or transient or vulnerab$)).ti,bt,ab. (226)

21 (house adj2 ("at risk" or deprived or disadvantage$ or "high risk" or inequitable or marginal$ or poverty or transient or vulnerab$)).ti,bt,ab. (21)

22 (houses adj2 ("at risk" or deprived or disadvantage$ or "high risk" or inequitable or marginal$ or poverty or transient or vulnerab$)).ti,bt,ab. (12)

23 (home$ adj2 ("at risk" or deprived or disadvantage$ or "high risk" or inequitable or marginal$ or poverty or transient or vulnerab$)).ti,bt,ab. (973)

24 (overcrowd$ adj2 (home$ or house or houses or housing)).ti,bt,ab. (150)

25 11 or 12 or 13 or 14 or 15 or 16 or 17 or 18 or 19 or 20 or 21 or 22 or 23 or 24 (22296)

26 *Unemployment/ (3408)

27 *Public Assistance/ (1677)

28 (unemployed or unemployment or workless$).ti,bt,ab. (17017)

29 (universal credit or incapacity benefit$ or income support$).ti,bt,ab. (334)

30 ((welfare or disability) adj2 benefit$).ti,bt,ab. (1645)

31 "welfare to work".ti,bt,ab,kw. (253)

32 ("minimum wage" or "minimum pay" or "low wage" or "low pay").ti,bt,ab. (936)

33 (deprivation or financial hardship or poverty).ti,bt,ab. (87722)

34 ((benefit$ or social or welfare) adj2 claimant$).ti,bt,ab. (60)

35 ((arrear$ or debt$ or deprived or low income$ or low-income$ or low pay$ or low paid) adj2 (communit$ or famil$ or group$ or neighborhood$ or neighbourhood$ or people or person$ or population$)).ti,bt,ab. (9980)

36 26 or 27 or 28 or 29 or 30 or 31 or 32 or 33 or 34 or 35 (116382)

37 *Food Assistance/ (699)

38 (food adj2 (bank$ or desert$ or environment$ or insecurity or pantry$ or program$ or stamp$ or supplemental or voucher$)).ti,bt,ab,kw. (11590)

39 "special supplemental nutrition program for women infants and children".ti,bt,ab,kw. (624)

40 WIC.ti,bt,ab,kw. (1386)

41 "Supplemental nutrition assistance program".ti,bt,ab,kw. (433)

42 (SNAP and food).ti,bt,ab. (394)

43 37 or 38 or 39 or 40 or 41 or 42 (13375)

44 *Prisoners/ (12619)

45 (prisoner$ or offender$).ti,bt,ab. (18168)

46 (release$ adj2 (convict$ or correctional or custod$ or detainee$ or detention or felon$ or incarcerat$ or inmate$ or jail$ or offender$ or penitentiar$ or prisoner$ or "secure accommodat$" or "secure facilit$" or "secure unit$")).ti,bt,ab. (736)

47 (ex-convict$ or ex-felon$ or ex-inmate$ or ex-offender$ or ex-prisoner$).ti,bt,ab. (297)

48 ((correctional facilit$ or detention center$ or detention centre$ or jail$ or penal or penitentiar$ or prison$ or "secure accommodat$" or "secure facilit$" or "secure unit$") adj2 (convict$ or detainee$ or felon$ or inmate$ or population or remand$ or system$)).ti,bt,ab. (3334)

49 (parole or probation).ti,bt,ab. (1488)

50 (custodial adj1 (care or sentence)).ti,bt,ab. (191)

51 (incarceration or incarcerated or imprisonment).ti,bt,ab. (12256)

52 44 or 45 or 46 or 47 or 48 or 49 or 50 or 51 (35531)

53 exp *Homeless Persons/ (6830)

54 homelessness.ti,bt,ab. (4287)

55 (rough sleep$ or rough-sleep$ or runaway$).ti,bt,ab. (1611)

56 ((destitute or homeless$) adj2 (accommodat$ or individual$ or group$ or hostel$ or man or men or people or person$ or population or shelter$ or woman or women)).ti,bt,ab. (4037)

57 53 or 54 or 55 or 56 (11251)

58 *Gypsies/ (673)

59 (traveller$ or Gypsies or Gypsy or Gipsy or Gipsies or Romany or Romanies or Romani or Romanis or Rromani or Rromanis or Roma or Rroma).ti,bt,ab. (8510)

60 58 or 59 (8546)

61 *Sex workers/ (1573)

62 (prostitut$ or sex work$).ti,bt,ab. (9307)

63 61 or 62 (9413)

64 *"Emigration and Immigration"/ (15014)

65 *"Emigrants and Immigrants"/ (8365)

66 *"Transients and Migrants"/ (7977)

67 *Refugees/ (7970)

68 (immigrant$ or migrant$ or asylum or refugee$).ti,bt,ab. (48905)

69 (displaced adj2 (people or person$1)).ti,bt,ab. (884)

70 (undocumented adj2 (people or person$1)).ti,bt,ab. (31)

71 (born adj2 overseas).ti,bt,ab. (333)

72 ("foreign born" or "non uk born" or "non-uk born").ti,bt,ab. (3301)

73 64 or 65 or 66 or 67 or 68 or 69 or 70 or 71 or 72 (63921)

74 *Minority Groups/ (7710)

75 *Ethnic Groups/ (29479)

76 *African Americans/ (27684)

77 *African Continental Ancestry Group/ (16610)

78 *Asian Continental Ancestry Group/ (24637)

79 ethnic minorit$.ti,bt,ab. (10389)

80 ((black or minority or ethnic) adj2 (communit$ or group$ or man or men or people or person$ or population$ or woman or women)).ti,bt,ab. (62632)

81 ("people of colo?r" or "women of colo?r" or "men of colo?r").ti,bt,ab. (1157)

82 (West Indian$ or Afrocaribbean$).ti,bt,ab. (920)

83 74 or 75 or 76 or 77 or 78 or 79 or 80 or 81 or 82 (151599)

84 *Disabled Persons/ (30463)

85 *Persons with Hearing Impairments/ (2200)

86 *Visually Impaired Persons/ (1956)

87 *Mentally Disabled Persons/ (2886)

88 *Intellectual Disability/ (37982)

89 ((learning or mental$ of physical$ or intellectual$) adj2 (disabled or disabilit$ or handicap$ or impair$)).ti,bt,ab. (33001)

90 ((adult$ or individual$ or people or person$) adj2 (disabled or disability or handicap$ or impair$)).ti,bt,ab. (18220)

91 84 or 85 or 86 or 87 or 88 or 89 or 90 (111266)

92 10 or 25 or 36 or 43 or 52 or 57 or 60 or 63 or 73 or 83 or 91 (683163)

93 exp Obesity/ (206007)

94 exp Weight Gain/ or exp Weight Loss/ (69420)

95 (obese or obesity or overweight).ti,bt,ab. (301076)

96 (healthy adj2 (diet$ or eating)).ti,bt,ab. (12670)

97 exp Diet/ (272768)

98 (fruit$ adj2 (intake or consum$ or increase or portion$ or serving$ or frequenc$ or number$ or preference$ or choice$ or choos$)).ti,bt,ab. (9828)

99 (vegetable$ adj2 (intake or consum$ or increase or portion$ or serving$ or frequenc$ or number$ or preference$ or choice$ or choos$)).ti,bt,ab. (10035)

100 "5 a day".ti,bt,ab. (175)

101 "five a day".ti,bt,ab. (44)

102 ((food or diet$) adj (choice$ or frequenc$ or intake)).ti,bt,ab. (79399)

103 Feeding Behavior/ (80284)

104 food habits/ or food preferences/ (90350)

105 nutrition therapy/ or exp diet therapy/ or exp diet/ (282942)

106 93 or 94 or 95 or 96 or 97 or 98 or 99 or 100 or 101 or 102 or 103 or 104 or 105 (714991)

107 (comment or editorial or letter).pt. (1800905)

108 106 not 107 (686155)

109 (physical adj2 (fit$ or train$ or activ$ or endur$)).ti,bt,ab. (120841)

110 (exercis$ adj2 (fit$ or train$ or activ$ or endur$)).ti,bt,ab. (30529)

111 ((promot$ or uptak$ or encourag$ or increas$ or start$ or adher$) adj2 (exercis$ or gym$ or sport$ or fitness)).ti,bt,ab. (24403)

112 ((decreas$ or reduc$ or discourag$) adj2 (sedentary or deskbound)).ti,bt,ab. (1308)

113 (sport$ or walk$ or running or jogging or bicycling or biking or swimming).ti,bt,ab. (259490)

114 (active adj (travel$ or transport$ or commut$)).ti,bt,ab. (9026)

115 physical fitness/ (26824)

116 exp Recreation/ or exp Sports/ (202699)

117 exp Exercise Therapy/ or exp exercise/ (218787)

118 running/ or jogging/ or swimming/ or walking/ (66537)

119 109 or 110 or 111 or 112 or 113 or 114 or 115 or 116 or 117 or 118 (579361)

120 (letter or editorial or comment).pt. (1800905)

121 119 not 120 (558867)

122 exp smoking/ (144374)

123 (smoking or antismoking or anti-smoking).ti,bt,ab. (214614)

124 (smoker or smokers).ti,bt,ab. (84655)

125 tobacco/ or tobacco.ti,bt,ab. (107652)

126 122 or 123 or 124 or 125 (346305)

127 (letter or editorial or comment).pt. (1800905)

128 126 not 127 (333078)

129 exp Alcohol Drinking/ (67424)

130 exp Alcoholic Intoxication/ (12385)

131 exp Alcoholic Beverages/ (19701)

132 exp Drinking Behavior/ (73834)

133 (beer or wine$ or cider or alcopop$ or spirit or spirits).ti,bt,ab. (33431)

134 alcohol$.ti,bt,ab. (322016)

135 (drink$ adj2 (binge or excessive or harm$ or heavy or misus$ or abus$ or consum$)).ti,bt,ab. (18509)

136 (intoxicat$ or inebriat$ or drunk$).ti,bt,ab. (49518)

137 129 or 130 or 131 or 132 or 133 or 134 or 135 or 136 (414686)

138 (comment or editorial or letter).pt. (1800905)

139 137 not 138 (403447)

140 Lifestyle/ (55338)

141 (lifestyle adj2 (intervention$ or program$ or chang$ or modif$ or improv$ or enhanc$)).ti,bt,ab. (24377)

142 (life style adj2 (intervention$ or program$ or chang$ or modif$ or improv$ or enhanc$)).ti,bt,ab. (2060)

143 (health adj2 (intervention$ or program$ or chang$ or modif$ or improv$ or enhanc$)).ti,bt,ab. (124691)

144 (behavior$ change adj (intervention$ or program$)).ti,bt,ab. (983)

145 (behaviour$ change adj (intervention$ or program$)).ti,bt,ab. (681)

146 (health behavior$ adj (program$ or intervention$)).ti,bt,ab. (251)

147 (health behaviour$ adj (program$ or intervention$)).ti,bt,ab. (80)

148 140 or 141 or 142 or 143 or 144 or 145 or 146 or 147 (194756)

149 92 and 108 (41254)

150 92 and 121 (25391)

151 92 and 128 (24749)

152 92 and 139 (24224)

153 92 and 148 (27472)

154 149 or 150 or 151 or 152 or 153 (113024)

155 systematic review.pt. (119940)

156 systematic$ review$.ti,ab. (163486)

157 meta-analysis as topic/ (17539)

158 meta-analytic$.ti,ab. (6956)

159 meta-analysis.ti,ab,pt. (165441)

160 metanalysis.ti,ab. (213)

161 metaanalysis.ti,ab. (1554)

162 meta analysis.ti,ab. (139269)

163 meta-synthesis.ti,ab. (813)

164 metasynthesis.ti,ab. (305)

165 meta synthesis.ti,ab. (813)

166 meta-regression.ti,ab. (7246)

167 metaregression.ti,ab. (640)

168 meta regression.ti,ab. (7246)

169 (synthes$ adj3 literature).ti,ab. (3317)

170 (synthes$ adj3 evidence).ti,ab. (9985)

171 integrative review.ti,ab. (2809)

172 data synthesis.ti,ab. (10923)

173 (research synthesis or narrative synthesis).ti,ab. (2958)

174 (systematic study or systematic studies).ti,ab. (11775)

175 (systematic comparison$ or systematic overview$).ti,ab. (3269)

176 evidence based review.ti,ab. (1959)

177 comprehensive review.ti,ab. (14279)

178 critical review.ti,ab. (15306)

179 quantitative review.ti,ab. (654)

180 structured review.ti,ab. (812)

181 realist review.ti,ab. (289)

182 realist synthesis.ti,ab. (196)

183 or/155-182 (340181)

184 review.pt. (2599616)

185 medline.ab. (111185)

186 pubmed.ab. (108173)

187 cochrane.ab. (78189)

188 embase.ab. (85122)

189 cinahl.ab. (25843)

190 psyc?lit.ab. (918)

191 psyc?info.ab. (33124)

192 (literature adj3 search$).ab. (57876)

193 (database$ adj3 search$).ab. (57630)

194 (bibliographic adj3 search$).ab. (2488)

195 (electronic adj3 search$).ab. (21247)

196 (electronic adj3 database$).ab. (27895)

197 (computeri?ed adj3 search$).ab. (3499)

198 (internet adj3 search$).ab. (3152)

199 included studies.ab. (22541)

200 (inclusion adj3 studies).ab. (15707)

201 inclusion criteria.ab. (81873)

202 selection criteria.ab. (29725)

203 predefined criteria.ab. (1898)

204 predetermined criteria.ab. (1040)

205 (assess$ adj3 (quality or validity)).ab. (76995)

206 (select$ adj3 (study or studies)).ab. (64679)

207 (data adj3 extract$).ab. (60528)

208 extracted data.ab. (13911)

209 (data adj2 abstracted).ab. (5194)

210 (data adj3 abstraction).ab. (1654)

211 published intervention$.ab. (173)

212 ((study or studies) adj2 evaluat$).ab. (180675)

213 (intervention$ adj2 evaluat$).ab. (10981)

214 confidence interval$.ab. (400789)

215 heterogeneity.ab. (160135)

216 pooled.ab. (86279)

217 pooling.ab. (11950)

218 odds ratio$.ab. (261475)

219 (Jadad or coding).ab. (178225)

220 or/185-219 (1405926)

221 184 and 220 (238085)

222 review.ti. (454352)

223 222 and 220 (138011)

224 (review$ adj4 (papers or trials or studies or evidence or intervention$ or evaluation$)).ti,ab. (181214)

225 183 or 221 or 223 or 224 (556194)

226 letter.pt. (1058680)

227 editorial.pt. (514719)

228 comment.pt. (824142)

229 226 or 227 or 228 (1800905)

230 225 not 229 (543078)

231 154 and 230 (5020)

232 limit 231 to yr="2009 -Current" (3996)

233 animals/ not (humans/ and animals/) (4630525)

234 232 not 233 (3986)

235 (child$ or childhood$ or children$).ti,bt. (733448)

236 234 not 235 (3625)

237 234 not 236 (361)

238 (adult$ or parent$ or woman$ or women$ or mother$ or maternal$ or man or mans or men mens or father$ or paternal$).ti,bt. (898675)

239 237 and 238 (68)

240 236 or 239 (3693)

**1.2 MEDLINE strategy 2**

Database: Ovid MEDLINE(R) ALL <1946 to January 13, 2020>

Search Strategy:

--------------------------------------------------------------------------------

1 Nutrition Policy/ (9081)

2 (Food/ or Food Industry/) and Government Regulation/ (223)

3 (Food adj2 (policy or policies)).ti,bt,ab. (1081)

4 Food/ and Mass Media/ (51)

5 Food Labelling/ or Food Packaging/ (8010)

6 (Food/ or Food Industry/) and Marketing/ (423)

7 (Food/ or Food Industry/) and Social Marketing/ (55)

8 (Food/ or Food Industry/) and Social Control, Formal/ (35)

9 (Food/ or Food Industry/) and Public Policy/ (202)

10 (Food/ or Food Industry/) and Social Environment/ (121)

11 food subsid$.ti,bt,ab. (103)

12 (food and (macro level intervention$ or media campaign$ or public health intervention$ or (population adj3 intervention$) or whole community intervention$)).ti,bt,ab. (721)

13 or/1-12 (18880)

14 (Diet/ or Healthy Diet/) and Government Regulation/ (59)

15 (Diet/ or Healthy Diet/) and Marketing/ (178)

16 (Diet/ or Healthy Diet/) and Social Marketing/ (61)

17 (Diet/ or Healthy Diet/) and Social Control, Formal/ (7)

18 (Diet/ or Healthy Diet/) and Public Policy/ (113)

19 (Diet/ or Healthy/) and Social Environment/ (414)

20 Nutritional Requirements/ and Government Regulation/ (14)

21 Nutritional Requirements/ and Public Policy/ (18)

22 or/14-21 (829)

23 Food, Fortified/ (9176)

24 ((flour or food$ or iodine$ or iron) adj2 (fortified or fortifies or fortify$ or fortification)).ti,bt,ab. (3611)

25 or/23-24 (11057)

26 Beverages/ (14772)

27 Dietary Sugars/ (589)

28 Carbonated Beverages/ (2815)

29 Sugars/ (1312)

30 Diet, Sodium Restricted/ (6210)

31 Sodium Chloride, Dietary/ (6766)

32 snacks/ or candy/ (2755)

33 26 or 27 or 28 or 29 or 30 or 31 or 32 (33076)

34 Government Regulation/ (20943)

35 Marketing/ or Social Marketing/ (7712)

36 Social Control, Formal/ (11738)

37 Public Policy/ (31060)

38 Social Environment/ (42301)

39 Mass Media/ (10782)

40 (macro level intervention$ or media campaign$ or public health intervention$ or (population adj3 intervention$) or whole community intervention$).ti,bt,ab. (13287)

41 or/34-40 (127132)

42 33 and 41 (524)

43 ((carbonated or fizzy or soft or sweetened) adj2 (beverage$ or drink$)).ti,bt,ab. (7532)

44 (salt adj2 (less or lessen or reduc$ or restrict$)).ti,bt,ab. (3949)

45 43 or 44 (11468)

46 (initiative$ or policies or policy or regulation$ or law$ or tax or taxation or taxes).ti,bt,ab. (1263760)

47 (macro level intervention$ or media campaign$ or public health intervention$ or (population adj3 intervention$) or whole community intervention$).ti,bt,ab. (13287)

48 46 or 47 (1274831)

49 45 and 48 (1485)

50 13 or 22 or 25 or 42 or 49 (31196)

51 Alcoholic Beverages/ and Government Regulation/ (80)

52 (alcohol adj2 (policy or policies)).ti,bt,ab. (1234)

53 Alcoholic Beverages/ and Mass Media/ (31)

54 Alcoholic Beverages/ and Marketing/ (164)

55 Alcoholic Beverages/ and Social Marketing/ (11)

56 Alcoholic Beverages/ and Social Control, Formal/ (38)

57 Alcoholic Beverages/ and Public Policy/ (206)

58 Alcoholic Beverages/ and Social Environment/ (166)

59 Alcoholic Beverages/ and Taxation/ (258)

60 (alcohol$ and (macro level intervention$ or media campaign$ or public health intervention$ or (population adj3 intervention$) or whole community intervention$)).ti,bt,ab. (734)

61 (alcohol$ adj3 (initiative$ or policies or policy or regulation$ or law$ or tax or taxation or taxes)).ti,bt,ab. (2559)

62 (alcohol adj6 (price or prices or priced or pricing)).ti,bt,ab. (499)

63 or/51-62 (4055)

64 (exp Smoking/ or exp "Tobacco Use"/ or exp Tobacco Products/) and Government Regulation/ (520)

65 ((smoking or tobacco or cigarette$) adj3 (policy or policies)).ti,bt,ab. (3568)

66 (exp Smoking/ or exp "Tobacco Use"/ or exp Tobacco Products/) and Mass Media/ (478)

67 (exp Smoking/ or exp "Tobacco Use"/ or exp Tobacco Products/) and Marketing/ (674)

68 (exp Smoking/ or exp "Tobacco Use"/ or exp Tobacco Products/) and Social Marketing/ (233)

69 (exp Smoking/ or exp "Tobacco Use"/ or exp Tobacco Products/) and Social Control, Formal/ (189)

70 (exp Smoking/ or exp "Tobacco Use"/ or exp Tobacco Products/) and Public Policy/ (782)

71 (exp Smoking/ or exp "Tobacco Use"/ or exp Tobacco Products/) and Social Environment/ (1274)

72 (exp Smoking/ or exp "Tobacco Use"/ or exp Tobacco Products/) and Taxation/ (1095)

73 ((smoking or tobacco or cigarette$) and (macro level intervention$ or media campaign$ or public health intervention$ or (population adj3 intervention$) or whole community intervention$)).ti,bt,ab. (1553)

74 ((smoking or tobacco or cigarette$) adj3 (initiative$ or policies or policy or regulation$ or law$ or tax or taxation or taxes)).ti,bt,ab. (6236)

75 ((smoking or tobacco or cigarette$) adj3 (price or prices or priced or pricing)).ti,bt,ab. (944)

76 ((smoking or tobacco or cigarette$) adj3 (ban or banned or bans or prohibit$)).ti,bt,ab. (2846)

77 or/64-76 (12868)

78 (Transportation/ or Mass Transit/ or Walking/ or Bicycling/) and Government Regulation/ (46)

79 ((transport$ or bus or buses or rail or car or cars or bicycling or cycling) adj3 (policy or policies)).ti,bt,ab. (403)

80 (Transportation/ or Mass Transit/ or Walking/ or Bicycling/) and Mass Media/ (36)

81 (Transportation/ or Mass Transit/ or Walking/ or Bicycling/) and Marketing/ (19)

82 (Transportation/ or Mass Transit/ or Walking/ or Bicycling/) and Social Marketing/ (69)

83 (Transportation/ or Mass Transit/ or Walking/ or Bicycling/) and Social Control, Formal/ (23)

84 (Transportation/ or Mass Transit/ or Walking/ or Bicycling/) and Public Policy/ (209)

85 (Transportation/ or Mass Transit/ or Walking/ or Bicycling/) and Social Environment/ (372)

86 (Transportation/ or Mass Transit/ or Walking/ or Bicycling/) and Taxation/ (17)

87 (Transportation/ or Mass Transit/ or Walking/ or Bicycling/) and Built Environment/ (61)

88 ((transport$ or car or cars or bicycling or bus or buses or cycling or walking or commuting) and (macro level intervention$ or media campaign$ or public health intervention$ or (population adj3 intervention$) or whole community intervention$ or environment$ intervention$)).ti,bt,ab. (483)

89 ((transport$ or commut$ or walk$ or bicycling or cycling) adj3 (initiative$ or policies or policy or regulation$ or law$ or tax or taxation or taxes)).ti,bt,ab. (6915)

90 ((fuel or petrol) adj3 (price or prices or priced or pricing)).ti,bt,ab. (137)

91 ((driving or motor$ or vehicle$) adj3 (ban or banned or bans or prohibit$)).ti,bt,ab. (133)

92 Exercise/ and Built Environment/ (56)

93 physical$ activit$.ti,bt,ab. and Built Environment/ (68)

94 ((physical$ activit$ or exercis$) and (macro level intervention$ or media campaign$ or public health intervention$ or (population adj3 intervention$) or whole community intervention$ or environment$ intervention$)).ti,bt,ab. (1465)

95 or/78-94 (9720)

96 50 or 63 or 77 or 95 (56636)

97 systematic review.pt. (119940)

98 (systematic$ adj2 review$).ti,ab. (171579)

99 meta-analysis as topic/ (17539)

100 meta-analytic$.ti,ab. (6956)

101 meta-analysis.ti,ab,pt. (165441)

102 metanalysis.ti,ab. (213)

103 metaanalysis.ti,ab. (1554)

104 meta analysis.ti,ab. (139269)

105 meta-synthesis.ti,ab. (813)

106 metasynthesis.ti,ab. (305)

107 meta synthesis.ti,ab. (813)

108 meta-regression.ti,ab. (7246)

109 metaregression.ti,ab. (640)

110 meta regression.ti,ab. (7246)

111 (synthes$ adj3 literature).ti,ab. (3317)

112 (synthes$ adj3 evidence).ti,ab. (9985)

113 integrative review.ti,ab. (2809)

114 data synthesis.ti,ab. (10923)

115 (research synthesis or narrative synthesis).ti,ab. (2958)

116 (systematic study or systematic studies).ti,ab. (11775)

117 (systematic comparison$ or systematic overview$).ti,ab. (3269)

118 evidence based review.ti,ab. (1959)

119 comprehensive review.ti,ab. (14279)

120 critical review.ti,ab. (15306)

121 quantitative review.ti,ab. (654)

122 structured review.ti,ab. (812)

123 realist review.ti,ab. (289)

124 realist synthesis.ti,ab. (196)

125 pooled analysis.ti,ab. (8188)

126 or/97-125 (347563)

127 review.pt. (2599616)

128 medline.ab. (111185)

129 pubmed.ab. (108173)

130 cochrane.ab. (78189)

131 embase.ab. (85122)

132 cinahl.ab. (25843)

133 psyc?lit.ab. (918)

134 psyc?info.ab. (33124)

135 (literature adj3 search$).ab. (57876)

136 (database$ adj3 search$).ab. (57630)

137 (bibliographic adj3 search$).ab. (2488)

138 (electronic adj3 search$).ab. (21247)

139 (electronic adj3 database$).ab. (27895)

140 (computeri?ed adj3 search$).ab. (3499)

141 (internet adj3 search$).ab. (3152)

142 included studies.ab. (22541)

143 (inclusion adj3 studies).ab. (15707)

144 inclusion criteria.ab. (81873)

145 selection criteria.ab. (29725)

146 predefined criteria.ab. (1898)

147 predetermined criteria.ab. (1040)

148 (assess$ adj3 (quality or validity)).ab. (76995)

149 (select$ adj3 (study or studies)).ab. (64679)

150 (data adj3 extract$).ab. (60528)

151 extracted data.ab. (13911)

152 (data adj2 abstracted).ab. (5194)

153 (data adj3 abstraction).ab. (1654)

154 published intervention$.ab. (173)

155 ((study or studies) adj2 evaluat$).ab. (180675)

156 (intervention$ adj2 evaluat$).ab. (10981)

157 confidence interval$.ab. (400789)

158 heterogeneity.ab. (160135)

159 pooled.ab. (86279)

160 pooling.ab. (11950)

161 odds ratio$.ab. (261475)

162 (Jadad or coding).ab. (178225)

163 or/128-162 (1405926)

164 127 and 163 (238085)

165 review.ti. (454352)

166 165 and 163 (138011)

167 (review$ adj4 (papers or trials or studies or evidence or intervention$ or evaluation$)).ti,ab. (181214)

168 126 or 164 or 166 or 167 (561912)

169 letter.pt. (1058680)

170 editorial.pt. (514719)

171 comment.pt. (824142)

172 169 or 170 or 171 (1800905)

173 168 not 172 (548648)

174 exp animals/ not humans/ (4663639)

175 173 not 174 (535154)

176 96 and 175 (2503)

177 (child$ or childhood$ or children$).ti,bt. (733448)

178 176 not 177 (2283)

179 176 not 178 (220)

180 (adult$ or parent$ or woman$ or women$ or mother$ or maternal$ or man or mans or men or mens or father$ or paternal$).ti,bt. (967232)

181 179 and 180 (23)

182 178 or 181 (2306)

183 176 not 182 (197)

184 limit 182 to yr="2009 -Current" (1771)

**1.3 Embase strategy 1**

Database: Embase <1974 to 2020 January 13>

Search Strategy:

--------------------------------------------------------------------------------

1 *Poverty/ (12511)

2 *Vulnerable Population/ (3160)

3 *Socioeconomics/ (20820)

4 *Minority Health/ (338)

5 *Health Disparity/ (9085)

6 ((health or social$ or socioeconomic$ or socio-economic$ or racial$) adj2 (disadvantage$ or disparit$ or inequalit$)).ti,bt,ab. (43014)

7 (("at risk" or deprived or disadvantaged or disengaged or excluded or forgotten or "hard to reach" or hidden or "high risk" or inaccessible or inequitable or isolated or "low SES" or "low socioeconomic status" or marginal$ or neglected or transient or underserved or unengaged or vulnerable) adj2 (area$ or background$ or communit$ or demographic$ or group$ or inner cit$ or neighbourhood$ or neighborhood$ or population$ or rural or urban)).ti,bt,ab. (167188)

8 (("at risk" or deprived or disadvantaged or disengaged or excluded or forgotten or "hard to reach" or hidden or "high risk" or inequitable or isolated or "low SES" or "low socioeconomic status" or marginal$ or neglected or transient or underserved or unengaged or vulnerable) adj2 (adult$ or group$ or man or men or people or person$ or women or youth)).ti,bt,ab. (123664)

9 ((low level$ adj2 education) or low education level$ or low educational attainment or (low attainment adj2 education)).ti,bt,ab. (4036)

10 1 or 2 or 3 or 4 or 5 or 6 or 7 or 8 or 9 (293725)

11 *Housing/ (7905)

12 *Demography/ (19709)

13 (slum or slums or tenement$).ti,bt,ab. (3402)

14 tower block$.ti,bt,ab. (16)

15 (high-rise adj2 (block$ or building$)).ti,bt,ab. (321)

16 (housing adj2 (council or insecur$ or instability or poor or short-term or "short term" or social or temporary or unstable)).ti,bt,ab. (2800)

17 (house adj2 (council or insecur$ or instability or poor or short-term or "short term" or social or temporary or unstable)).ti,bt,ab. (138)

18 (houses adj2 (council or insecur$ or instability or poor or short-term or "short term" or social or temporary or unstable)).ti,bt,ab. (94)

19 (housing adj2 ("at risk" or deprived or disadvantage$ or "high risk" or inequitable or marginal$ or poverty or transient or vulnerab$)).ti,bt,ab. (291)

20 (house adj2 ("at risk" or deprived or disadvantage$ or "high risk" or inequitable or marginal$ or poverty or transient or vulnerab$)).ti,bt,ab. (23)

21 (houses adj2 ("at risk" or deprived or disadvantage$ or "high risk" or inequitable or marginal$ or poverty or transient or vulnerab$)).ti,bt,ab. (15)

22 (home$ adj2 ("at risk" or deprived or disadvantage$ or "high risk" or inequitable or marginal$ or poverty or transient or vulnerab$)).ti,bt,ab. (1244)

23 (overcrowd$ adj2 (home$ or house or houses or housing)).ti,bt,ab. (189)

24 11 or 12 or 13 or 14 or 15 or 16 or 17 or 18 or 19 or 20 or 21 or 22 or 23 (34886)

25 *Unemployment/ (3715)

26 *Social Care/ (2234)

27 (unemployed or unemployment or workless$).ti,bt,ab. (21967)

28 (universal credit or incapacity benefit$ or income support$).ti,bt,ab. (416)

29 ((welfare or disability) adj2 benefit$).ti,bt,ab. (1995)

30 "welfare to work".ti,bt,ab,kw. (256)

31 ("minimum wage" or "minimum pay" or "low wage" or "low pay").ti,bt,ab. (1044)

32 (deprivation or financial hardship or poverty).ti,bt,ab. (109962)

33 ((benefit$ or social or welfare) adj2 claimant$).ti,bt,ab. (71)

34 ((arrear$ or debt$ or deprived or low income$ or low-income$ or low pay$ or low paid) adj2 (communit$ or famil$ or group$ or neighborhood$ or neighbourhood$ or people or person$ or population$)).ti,bt,ab. (11531)

35 25 or 26 or 27 or 28 or 29 or 30 or 31 or 32 or 33 or 34 (145653)

36 *Food Assistance/ (588)

37 (food adj2 (bank$ or desert$ or environment$ or insecurity or pantry$ or program$ or stamp$ or supplemental or voucher$)).ti,bt,ab,kw. (14309)

38 "special supplemental nutrition program for women infants and children".ti,bt,ab,kw. (661)

39 WIC.ti,bt,ab,kw. (1652)

40 "Supplemental nutrition assistance program".ti,bt,ab,kw. (514)

41 (SNAP and food).ti,bt,ab. (553)

42 36 or 37 or 38 or 39 or 40 or 41 (16349)

43 *Prisoner/ (8464)

44 (prisoner$ or offender$).ti,bt,ab. (22174)

45 (release$ adj2 (convict$ or correctional or custod$ or detainee$ or detention or felon$ or incarcerat$ or inmate$ or jail$ or offender$ or penitentiar$ or prisoner$ or "secure accommodat$" or "secure facilit$" or "secure unit$")).ti,bt,ab. (888)

46 (ex-convict$ or ex-felon$ or ex-inmate$ or ex-offender$ or ex-prisoner$).ti,bt,ab. (323)

47 ((correctional facilit$ or detention center$ or detention centre$ or jail$ or penal or penitentiar$ or prison$ or "secure accommodat$" or "secure facilit$" or "secure unit$") adj2 (convict$ or detainee$ or felon$ or inmate$ or population or remand$ or system$)).ti,bt,ab. (3868)

48 (parole or probation).ti,bt,ab. (1951)

49 (custodial adj1 (care or sentence)).ti,bt,ab. (250)

50 (incarceration or incarcerated or imprisonment).ti,bt,ab. (14873)

51 43 or 44 or 45 or 46 or 47 or 48 or 49 or 50 (40672)

52 *Homelessness/ (5639)

53 homelessness.ti,bt,ab. (5194)

54 (rough sleep$ or rough-sleep$ or runaway$).ti,bt,ab. (1621)

55 ((destitute or homeless$) adj2 (accommodat$ or individual$ or group$ or hostel$ or man or men or people or person$ or population or shelter$ or woman or women)).ti,bt,ab. (4718)

56 52 or 53 or 54 or 55 (11930)

57 *"Romani (people)"/ (123)

58 (traveller$ or Gypsies or Gypsy or Gipsy or Gipsies or Romany or Romanies or Romani or Romanis or Rromani or Rromanis or Roma or Rroma).ti,bt,ab. (10903)

59 57 or 58 (10907)

60 *Sex worker/ (721)

61 (prostitut$ or sex work$).ti,bt,ab. (10194)

62 60 or 61 (10220)

63 exp *Migration/ (21408)

64 exp *Migrant/ (14769)

65 exp *Refugee/ (6939)

66 (immigrant$ or migrant$ or asylum or refugee$).ti,bt,ab. (54007)

67 (displaced adj2 (people or person$1)).ti,bt,ab. (929)

68 (undocumented adj2 (people or person$1)).ti,bt,ab. (38)

69 (born adj2 overseas).ti,bt,ab. (474)

70 ("foreign born" or "non uk born" or "non-uk born").ti,bt,ab. (3948)

71 63 or 64 or 65 or 66 or 67 or 68 or 69 or 70 (71289)

72 *Minority Group/ (5042)

73 *Ethnic Group/ (15620)

74 exp Black People/ (100688)

75 ethnic minorit$.ti,bt,ab. (12972)

76 ((black or minority or ethnic) adj2 (communit$ or group$ or man or men or people or person$ or population$ or woman or women)).ti,bt,ab. (79597)

77 ("people of colo?r" or "women of colo?r" or "men of colo?r").ti,bt,ab. (1312)

78 (West Indian$ or Afrocaribbean$).ti,bt,ab. (962)

79 72 or 73 or 74 or 75 or 76 or 77 or 78 (185525)

80 *Disabled Person/ (15805)

81 *Hearing Impaired Person/ (314)

82 *Visually Impaired Person/ (1581)

83 *Mentally Disabled Person/ (152)

84 *Intellectual Impairment/ (11083)

85 ((learning or mental$ of physical$ or intellectual$) adj2 (disabled or disabilit$ or handicap$ or impair$)).ti,bt,ab. (45032)

86 ((adult$ or individual$ or people or person$) adj2 (disabled or disability or handicap$ or impair$)).ti,bt,ab. (24975)

87 80 or 81 or 82 or 83 or 84 or 85 or 86 (85681)

88 10 or 24 or 35 or 42 or 51 or 56 or 59 or 62 or 71 or 79 or 87 (810601)

89 exp Obesity/ (499137)

90 exp Body Weight Gain/ or exp Body Weight Loss/ (43782)

91 (obese or obesity or overweight).ti,bt,ab. (444752)

92 (healthy adj2 (diet$ or eating)).ti,bt,ab. (17525)

93 exp Diet/ (309609)

94 (fruit$ adj2 (intake or consum$ or increase or portion$ or serving$ or frequenc$ or number$ or preference$ or choice$ or choos$)).ti,bt,ab. (12277)

95 (vegetable$ adj2 (intake or consum$ or increase or portion$ or serving$ or frequenc$ or number$ or preference$ or choice$ or choos$)).ti,bt,ab. (12577)

96 "5 a day".ti,bt,ab. (237)

97 "five a day".ti,bt,ab. (64)

98 ((food or diet$) adj (choice$ or frequenc$ or intake)).ti,bt,ab. (103150)

99 Feeding Behavior/ (79994)

100 Food preference/ (13150)

101 exp diet therapy/ or exp diet/ (586484)

102 89 or 90 or 91 or 92 or 93 or 94 or 95 or 96 or 97 or 98 or 99 or 100 or 101 (1205105)

103 (editorial or letter).pt. (1741606)

104 102 not 103 (1152333)

105 (physical adj2 (fit$ or train$ or activ$ or endur$)).ti,bt,ab. (163949)

106 (exercis$ adj2 (fit$ or train$ or activ$ or endur$)).ti,bt,ab. (41658)

107 ((promot$ or uptak$ or encourag$ or increas$ or start$ or adher$) adj2 (exercis$ or gym$ or sport$ or fitness)).ti,bt,ab. (31374)

108 ((decreas$ or reduc$ or discourag$) adj2 (sedentary or deskbound)).ti,bt,ab. (1681)

109 (sport$ or walk$ or running or jogging or bicycling or biking or swimming).ti,bt,ab. (341989)

110 (active adj (travel$ or transport$ or commut$)).ti,bt,ab. (9402)

111 Fitness/ (36361)

112 exp Recreation/ or exp Sport/ (223496)

113 exp kinesiotherapy/ or exp exercise/ (367517)

114 running/ or jogging/ or swimming/ or walking/ (112659)

115 105 or 106 or 107 or 108 or 109 or 110 or 111 or 112 or 113 or 114 (885589)

116 (letter or editorial).pt. (1741606)

117 115 not 116 (857335)

118 exp smoking/ (376067)

119 (smoking or antismoking or anti-smoking).ti,bt,ab. (309684)

120 (smoker or smokers).ti,bt,ab. (128770)

121 tobacco/ or tobacco.ti,bt,ab. (124822)

122 118 or 119 or 120 or 121 (523394)

123 (letter or editorial).pt. (1741606)

124 122 not 123 (500801)

125 exp Drinking Behavior/ (47458)

126 exp Alcoholic Intoxication/ (11959)

127 exp Alcoholic Beverages/ (28571)

128 exp Drinking Behavior/ (47458)

129 (beer or wine$ or cider or alcopop$ or spirit or spirits).ti,bt,ab. (40422)

130 alcohol$.ti,bt,ab. (435404)

131 (drink$ adj2 (binge or excessive or harm$ or heavy or misus$ or abus$ or consum$)).ti,bt,ab. (25814)

132 (intoxicat$ or inebriat$ or drunk$).ti,bt,ab. (61959)

133 125 or 126 or 127 or 129 or 130 or 131 or 132 (538316)

134 (editorial or letter).pt. (1741606)

135 133 not 134 (528219)

136 Lifestyle/ (109886)

137 (lifestyle adj2 (intervention$ or program$ or chang$ or modif$ or improv$ or enhanc$)).ti,bt,ab. (36253)

138 (life style adj2 (intervention$ or program$ or chang$ or modif$ or improv$ or enhanc$)).ti,bt,ab. (3879)

139 (health adj2 (intervention$ or program$ or chang$ or modif$ or improv$ or enhanc$)).ti,bt,ab. (150519)

140 (behavior$ change adj (intervention$ or program$)).ti,bt,ab. (1082)

141 (behaviour$ change adj (intervention$ or program$)).ti,bt,ab. (880)

142 (health behavior$ adj (program$ or intervention$)).ti,bt,ab. (268)

143 (health behaviour$ adj (program$ or intervention$)).ti,bt,ab. (97)

144 136 or 137 or 138 or 139 or 140 or 141 or 142 or 143 (280403)

145 88 and 104 (65738)

146 88 and 117 (37904)

147 88 and 124 (39052)

148 88 and 135 (32965)

149 88 and 144 (35877)

150 145 or 146 or 147 or 148 or 149 (162764)

151 systematic$ review$.ti,ab. (203855)

152 systematic$ literature review$.ti,ab. (15056)

153 "systematic review"/ (230584)

154 "systematic review (topic)"/ (24355)

155 meta analysis/ (179037)

156 "meta analysis (topic)"/ (41094)

157 meta-analytic$.ti,ab. (8174)

158 meta-analysis.ti,ab. (182238)

159 metanalysis.ti,ab. (583)

160 metaanalysis.ti,ab. (7783)

161 meta analysis.ti,ab. (182238)

162 meta-synthesis.ti,ab. (892)

163 metasynthesis.ti,ab. (362)

164 meta synthesis.ti,ab. (892)

165 meta-regression.ti,ab. (9121)

166 metaregression.ti,ab. (1048)

167 meta regression.ti,ab. (9121)

168 (synthes$ adj3 literature).ti,ab. (3874)

169 (synthes$ adj3 evidence).ti,ab. (11207)

170 (synthes$ adj2 qualitative).ti,ab. (2968)

171 integrative review.ti,ab. (2750)

172 data synthesis.ti,ab. (13153)

173 (research synthesis or narrative synthesis).ti,ab. (3291)

174 (systematic study or systematic studies).ti,ab. (12849)

175 (systematic comparison$ or systematic overview$).ti,ab. (3685)

176 (systematic adj2 search$).ti,ab. (31473)

177 systematic$ literature research$.ti,ab. (333)

178 (review adj3 scientific literature).ti,ab. (1826)

179 (literature review adj2 side effect$).ti,ab. (16)

180 (literature review adj2 adverse effect$).ti,ab. (3)

181 (literature review adj2 adverse event$).ti,ab. (16)

182 (evidence-based adj2 review).ti,ab. (3722)

183 comprehensive review.ti,ab. (16516)

184 critical review.ti,ab. (16485)

185 critical analysis.ti,ab. (8180)

186 quantitative review.ti,ab. (760)

187 structured review.ti,ab. (1095)

188 realist review.ti,ab. (310)

189 realist synthesis.ti,ab. (186)

190 (pooled adj2 analysis).ti,ab. (20147)

191 (pooled data adj6 (studies or trials)).ti,ab. (2987)

192 (medline and (inclusion adj3 criteria)).ti,ab. (25359)

193 (search adj (strateg$ or term$)).ti,ab. (37327)

194 or/151-193 (550125)

195 medline.ab. (138243)

196 pubmed.ab. (137989)

197 cochrane.ab. (101389)

198 embase.ab. (108150)

199 cinahl.ab. (30204)

200 psyc?lit.ab. (995)

201 psyc?info.ab. (30219)

202 lilacs.ab. (7791)

203 (literature adj3 search$).ab. (74102)

204 (database$ adj3 search$).ab. (72578)

205 (bibliographic adj3 search$).ab. (2931)

206 (electronic adj3 search$).ab. (26013)

207 (electronic adj3 database$).ab. (37765)

208 (computeri?ed adj3 search$).ab. (4227)

209 (internet adj3 search$).ab. (4276)

210 included studies.ab. (28521)

211 (inclusion adj3 studies).ab. (19600)

212 inclusion criteria.ab. (143024)

213 selection criteria.ab. (35767)

214 predefined criteria.ab. (2611)

215 predetermined criteria.ab. (1318)

216 (assess$ adj3 (quality or validity)).ab. (102990)

217 (select$ adj3 (study or studies)).ab. (85213)

218 (data adj3 extract$).ab. (83430)

219 extracted data.ab. (18172)

220 (data adj2 abstracted).ab. (8748)

221 (data adj3 abstraction).ab. (2461)

222 published intervention$.ab. (220)

223 ((study or studies) adj2 evaluat$).ab. (260649)

224 (intervention$ adj2 evaluat$).ab. (15477)

225 confidence interval$.ab. (486063)

226 heterogeneity.ab. (207269)

227 pooled.ab. (122333)

228 pooling.ab. (15999)

229 odds ratio$.ab. (331382)

230 (Jadad or coding).ab. (213017)

231 evidence-based.ti,ab. (141046)

232 or/195-231 (1976599)

233 review.pt. (2524356)

234 232 and 233 (246756)

235 review.ti. (517464)

236 232 and 235 (172509)

237 (review$ adj10 (papers or trials or trial data or studies or evidence or intervention$ or evaluation$ or outcome$ or findings)).ti,ab. (537801)

238 (retriev$ adj10 (papers or trials or studies or evidence or intervention$ or evaluation$ or outcome$ or findings)).ti,ab. (29019)

239 194 or 234 or 236 or 237 or 238 (1017998)

240 letter.pt. (1102013)

241 editorial.pt. (639593)

242 240 or 241 (1741606)

243 239 not 242 (998387)

244 (animal/ or nonhuman/) not exp human/ (5578909)

245 243 not 244 (963164)

246 150 and 245 (9392)

247 (child$ or childhood$ or children$).ti,bt. (823964)

248 246 not 247 (8712)

249 246 not 248 (680)

250 (adult$ or parent$ or woman$ or women$ or mother$ or maternal$ or man or mans or men mens or father$ or paternal$).ti,bt. (1043182)

251 249 and 250 (134)

252 248 or 251 (8846)

253 limit 252 to yr="2009 -Current" (7100)

254 limit 253 to embase (3941)

**1.4 Embase strategy 2**

Database: Embase <1974 to 2020 January 13>

Search Strategy:

--------------------------------------------------------------------------------

1 Nutrition Policy/ (1243)

2 (Food/ or Food Industry/) and Government Regulation/ (392)

3 (Food adj2 (policy or policies)).ti,bt,ab. (1308)

4 Food/ and Mass Medium/ (131)

5 Food Packaging/ (9915)

6 (Food/ or Food Industry/) and Marketing/ (1217)

7 (Food/ or Food Industry/) and Social Marketing/ (128)

8 (Food/ or Food Industry/) and Social Control, Formal/ (53)

9 (Food/ or Food Industry/) and Public Policy/ (49)

10 (Food/ or Food Industry/) and Social Environment/ (197)

11 food subsid$.ti,bt,ab. (97)

12 (food and (macro level intervention$ or media campaign$ or public health intervention$ or (population adj3 intervention$) or whole community intervention$)).ti,bt,ab. (879)

13 or/1-12 (14848)

14 (Diet/ or Healthy Diet/) and Government Regulation/ (67)

15 (Diet/ or Healthy Diet/) and Marketing/ (340)

16 (Diet/ or Healthy Diet/) and Social Marketing/ (83)

17 (Diet/ or Healthy Diet/) and Social Control, Formal/ (31)

18 (Diet/ or Healthy Diet/) and Public Policy/ (41)

19 (Diet/ or Healthy/) and Social Environment/ (362)

20 Nutritional Requirement/ and Government Regulation/ (29)

21 Nutritional Requirement/ and Public Policy/ (1)

22 or/14-21 (924)

23 Fortified Food/ (988)

24 ((flour or food$ or iodine$ or iron) adj2 (fortified or fortifies or fortify$ or fortification)).ti,bt,ab. (4490)

25 or/23-24 (5135)

26 Beverage/ (17847)

27 Sugar Intake/ (7665)

28 Carbonated Beverages/ (2769)

29 Sugar/ (26384)

30 Sodium Restriction/ (9085)

31 Salt Intake/ (10378)

32 Fast Food/ or Candy/ (8324)

33 26 or 27 or 28 or 29 or 30 or 31 or 32 (75080)

34 Government Regulation/ (26640)

35 Marketing/ or Social Marketing/ (27048)

36 Social Control, Formal/ (14341)

37 Public Policy/ (3585)

38 Social Environment/ (32371)

39 Mass Medium/ (18161)

40 (macro level intervention$ or media campaign$ or public health intervention$ or (population adj3 intervention$) or whole community intervention$).ti,bt,ab. (16708)

41 or/34-40 (130189)

42 33 and 41 (1410)

43 ((carbonated or fizzy or soft or sweetened) adj2 (beverage$ or drink$)).ti,bt,ab. (9779)

44 (salt adj2 (less or lessen or reduc$ or restrict$)).ti,bt,ab. (4961)

45 43 or 44 (14721)

46 (initiative$ or policies or policy or regulation$ or law$ or tax or taxation or taxes).ti,bt,ab. (1542925)

47 (macro level intervention$ or media campaign$ or public health intervention$ or (population adj3 intervention$) or whole community intervention$).ti,bt,ab. (16708)

48 46 or 47 (1556975)

49 45 and 48 (1879)

50 13 or 22 or 25 or 42 or 49 (22617)

51 Alcoholic Beverage/ and Government Regulation/ (98)

52 (alcohol adj2 (policy or policies)).ti,bt,ab. (1534)

53 Alcoholic Beverage/ and Mass Medium/ (41)

54 Alcoholic Beverage/ and Marketing/ (207)

55 Alcoholic Beverage/ and Social Marketing/ (18)

56 Alcoholic Beverage/ and Social Control, Formal/ (26)

57 Alcoholic Beverage/ and Public Policy/ (51)

58 Alcoholic Beverage/ and Social Environment/ (83)

59 Alcoholic Beverage/ and Taxation/ (1)

60 (alcohol$ and (macro level intervention$ or media campaign$ or public health intervention$ or (population adj3 intervention$) or whole community intervention$)).ti,bt,ab. (901)

61 (alcohol$ adj3 (initiative$ or policies or policy or regulation$ or law$ or tax or taxation or taxes)).ti,bt,ab. (3296)

62 (alcohol adj6 (price or prices or priced or pricing)).ti,bt,ab. (603)

63 or/51-62 (4857)

64 (exp Smoking/ or exp "Tobacco Use"/) and Government Regulation/ (794)

65 ((smoking or tobacco or cigarette$) adj3 (policy or policies)).ti,bt,ab. (4080)

66 (exp Smoking/ or exp "Tobacco Use"/) and Mass Medium/ (1076)

67 (exp Smoking/ or exp "Tobacco Use"/) and Marketing/ (1353)

68 (exp Smoking/ or exp "Tobacco Use"/) and Social Marketing/ (327)

69 (exp Smoking/ or exp "Tobacco Use"/) and Social Control, Formal/ (277)

70 (exp Smoking/ or exp "Tobacco Use"/) and Public Policy/ (204)

71 (exp Smoking/ or exp "Tobacco Use"/) and Social Environment/ (1112)

72 (exp Smoking/ or exp "Tobacco Use"/) and Taxation/ (17)

73 ((smoking or tobacco or cigarette$) and (macro level intervention$ or media campaign$ or public health intervention$ or (population adj3 intervention$) or whole community intervention$)).ti,bt,ab. (1881)

74 ((smoking or tobacco or cigarette$) adj3 (initiative$ or policies or policy or regulation$ or law$ or tax or taxation or taxes)).ti,bt,ab. (7295)

75 ((smoking or tobacco or cigarette$) adj3 (price or prices or priced or pricing)).ti,bt,ab. (1046)

76 ((smoking or tobacco or cigarette$) adj3 (ban or banned or bans or prohibit$)).ti,bt,ab. (3453)

77 or/64-76 (15179)

78 ("Traffic and Transport"/ or Walking/ or Cycling/) and Government Regulation/ (83)

79 ((transport$ or bus or buses or rail or car or cars or bicycling or cycling) adj3 (policy or policies)).ti,bt,ab. (483)

80 ("Traffic and Transport"/ or Walking/ or Cycling/) and Mass Medium/ (95)

81 ("Traffic and Transport"/ or Walking/ or Cycling/) and Marketing/ (108)

82 ("Traffic and Transport"/ or Walking/ or Cycling/) and Social Marketing/ (63)

83 ("Traffic and Transport"/ or Walking/ or Cycling/) and Social Control, Formal/ (31)

84 ("Traffic and Transport"/ or Walking/ or Cycling/) and Public Policy/ (36)

85 ("Traffic and Transport"/ or Walking/ or Cycling/) and Social Environment/ (425)

86 ("Traffic and Transport"/ or Walking/ or Cycling/) and Taxation/ (1)

87 ("Traffic and Transport"/ or Walking/ or Cycling/) and Built Environment/ (67)

88 ((transport$ or car or cars or bicycling or bus or buses or cycling or walking or commuting) and (macro level intervention$ or media campaign$ or public health intervention$ or (population adj3 intervention$) or whole community intervention$ or environment$ intervention$)).ti,bt,ab. (615)

89 ((transport$ or commut$ or walk$ or bicycling or cycling) adj3 (initiative$ or policies or policy or regulation$ or law$ or tax or taxation or taxes)).ti,bt,ab. (8018)

90 ((fuel or petrol) adj3 (price or prices or priced or pricing)).ti,bt,ab. (181)

91 ((driving or motor$ or vehicle$) adj3 (ban or banned or bans or prohibit$)).ti,bt,ab. (200)

92 (Exercise/ or Physical Activity/) and Built Environment/ (124)

93 physical$ activit$.ti,bt,ab. and Built Environment/ (133)

94 ((physical$ activit$ or exercis$) and (macro level intervention$ or media campaign$ or public health intervention$ or (population adj3 intervention$) or whole community intervention$ or environment$ intervention$)).ti,bt,ab. (1868)

95 or/78-94 (11550)

96 50 or 63 or 77 or 95 (52600)

97 systematic$ review$.ti,ab. (203855)

98 systematic$ literature review$.ti,ab. (15056)

99 "systematic review"/ (230584)

100 "systematic review (topic)"/ (24355)

101 meta analysis/ (179037)

102 "meta analysis (topic)"/ (41094)

103 meta-analytic$.ti,ab. (8174)

104 meta-analysis.ti,ab. (182238)

105 metanalysis.ti,ab. (583)

106 metaanalysis.ti,ab. (7783)

107 meta analysis.ti,ab. (182238)

108 meta-synthesis.ti,ab. (892)

109 metasynthesis.ti,ab. (362)

110 meta synthesis.ti,ab. (892)

111 meta-regression.ti,ab. (9121)

112 metaregression.ti,ab. (1048)

113 meta regression.ti,ab. (9121)

114 (synthes$ adj3 literature).ti,ab. (3874)

115 (synthes$ adj3 evidence).ti,ab. (11207)

116 (synthes$ adj2 qualitative).ti,ab. (2968)

117 integrative review.ti,ab. (2750)

118 data synthesis.ti,ab. (13153)

119 (research synthesis or narrative synthesis).ti,ab. (3291)

120 (systematic study or systematic studies).ti,ab. (12849)

121 (systematic comparison$ or systematic overview$).ti,ab. (3685)

122 (systematic adj2 search$).ti,ab. (31473)

123 systematic$ literature research$.ti,ab. (333)

124 (review adj3 scientific literature).ti,ab. (1826)

125 (literature review adj2 side effect$).ti,ab. (16)

126 (literature review adj2 adverse effect$).ti,ab. (3)

127 (literature review adj2 adverse event$).ti,ab. (16)

128 (evidence-based adj2 review).ti,ab. (3722)

129 comprehensive review.ti,ab. (16516)

130 critical review.ti,ab. (16485)

131 critical analysis.ti,ab. (8180)

132 quantitative review.ti,ab. (760)

133 structured review.ti,ab. (1095)

134 realist review.ti,ab. (310)

135 realist synthesis.ti,ab. (186)

136 (pooled adj2 analysis).ti,ab. (20147)

137 (pooled data adj6 (studies or trials)).ti,ab. (2987)

138 (medline and (inclusion adj3 criteria)).ti,ab. (25359)

139 (search adj (strateg$ or term$)).ti,ab. (37327)

140 or/97-139 (550125)

141 medline.ab. (138243)

142 pubmed.ab. (137989)

143 cochrane.ab. (101389)

144 embase.ab. (108150)

145 cinahl.ab. (30204)

146 psyc?lit.ab. (995)

147 psyc?info.ab. (30219)

148 lilacs.ab. (7791)

149 (literature adj3 search$).ab. (74102)

150 (database$ adj3 search$).ab. (72578)

151 (bibliographic adj3 search$).ab. (2931)

152 (electronic adj3 search$).ab. (26013)

153 (electronic adj3 database$).ab. (37765)

154 (computeri?ed adj3 search$).ab. (4227)

155 (internet adj3 search$).ab. (4276)

156 included studies.ab. (28521)

157 (inclusion adj3 studies).ab. (19600)

158 inclusion criteria.ab. (143024)

159 selection criteria.ab. (35767)

160 predefined criteria.ab. (2611)

161 predetermined criteria.ab. (1318)

162 (assess$ adj3 (quality or validity)).ab. (102990)

163 (select$ adj3 (study or studies)).ab. (85213)

164 (data adj3 extract$).ab. (83430)

165 extracted data.ab. (18172)

166 (data adj2 abstracted).ab. (8748)

167 (data adj3 abstraction).ab. (2461)

168 published intervention$.ab. (220)

169 ((study or studies) adj2 evaluat$).ab. (260649)

170 (intervention$ adj2 evaluat$).ab. (15477)

171 confidence interval$.ab. (486063)

172 heterogeneity.ab. (207269)

173 pooled.ab. (122333)

174 pooling.ab. (15999)

175 odds ratio$.ab. (331382)

176 (Jadad or coding).ab. (213017)

177 evidence-based.ti,ab. (141046)

178 or/141-177 (1976599)

179 review.pt. (2524356)

180 178 and 179 (246756)

181 review.ti. (517464)

182 178 and 181 (172509)

183 (review$ adj10 (papers or trials or trial data or studies or evidence or intervention$ or evaluation$ or outcome$ or findings)).ti,ab. (537801)

184 (retriev$ adj10 (papers or trials or studies or evidence or intervention$ or evaluation$ or outcome$ or findings)).ti,ab. (29019)

185 140 or 180 or 182 or 183 or 184 (1017998)

186 letter.pt. (1102013)

187 editorial.pt. (639593)

188 186 or 187 (1741606)

189 185 not 188 (998387)

190 (animal/ or nonhuman/) not exp human/ (5578909)

191 189 not 190 (963164)

192 96 and 191 (3154)

193 (child$ or childhood$ or children$).ti,bt. (823964)

194 192 not 193 (2858)

195 192 not 194 (296)

196 (adult$ or parent$ or woman$ or women$ or mother$ or maternal$ or man or mans or men or mens or father$ or paternal$).ti,bt. (1125840)

197 195 and 196 (22)

198 194 or 197 (2880)

**1.5 Further searches for care leavers**

The original database searches did not include care leavers so additional searches for care leavers were undertaken in August 2020.

**MEDLINE strategy**

MeSH terms used for disadvantaged groups were restricted to Major focus.

Database: Ovid MEDLINE(R) ALL <1946 to August 07, 2020>

Search Strategy:

--------------------------------------------------------------------------------

1 (care adj4 leave$).ti,ab,kf. (755)

2 looked after child$.ti,ab. (113)

3 looked-after child$.ti,ab. (113)

4 Foster Home Care/ or Residential Facilities/ (8962)

5 Transition to Adult Care/ (1430)

6 4 and 5 (9)

7 1 or 2 or 3 or 6 (866)

8 (healthy adj2 (diet$ or eating)).ti,ab. (13537)

9 (fruit$ adj2 (intake or consum$ or increase or portion$ or serving$ or frequenc$ or number$ or preference$ or choice$ or choos$)).ti,ab. (10222)

10 (vegetable$ adj2 (intake or consum$ or increase or portion$ or serving$ or frequenc$ or number$ or preference$ or choice$ or choos$)).ti,ab. (10427)

11 "5 a day".ti,ab. (175)

12 "five a day".ti,ab. (46)

13 ((food or diet$) adj (choice$ or frequenc$ or intake)).ti,ab. (82008)

14 Feeding Behavior/ (82309)

15 food habits/ or food preferences/ (92735)

16 nutrition therapy/ or exp diet therapy/ or exp diet/ (290879)

17 8 or 9 or 10 or 11 or 12 or 13 or 14 or 15 or 16 (414112)

18 (comment or editorial or letter).pt. (1872653)

19 17 not 18 (401606)

20 (physical adj3 (fit$ or train$ or activ$ or endur$)).ti,ab. (130981)

21 (exercis$ adj3 (fit$ or train$ or activ$ or endur$)).ti,ab. (39368)

22 ((promot$ or uptak$ or encourag$ or increas$ or start$ or adher$) adj3 (exercis$ or gym$ or sport$ or fitness)).ti,ab. (35160)

23 ((decreas$ or reduc$ or discourag$) adj3 (sedentary or deskbound)).ti,ab. (1689)

24 (sport$ or walk$ or running or jogging or bicycling or biking or swimming).ti,ab. (270863)

25 (active adj (travel$ or transport$ or commut$)).ti,ab. (9239)

26 physical fitness/ (27278)

27 exp Recreation/ (208563)

28 exp Exercise Therapy/ or exp exercise/ (227542)

29 running/ or jogging/ or swimming/ or walking/ (68696)

30 20 or 21 or 22 or 23 or 24 or 25 or 26 or 27 or 28 or 29 (609737)

31 (letter or editorial or comment).pt. (1872653)

32 30 not 31 (588212)

33 exp smoking/ (147336)

34 (smoking or antismoking or anti-smoking).ti,ab. (222158)

35 (smoker or smokers).ti,ab. (87419)

36 tobacco/ or tobacco.ti,ab. (111318)

37 33 or 34 or 35 or 36 (357644)

38 (letter or editorial or comment).pt. (1872653)

39 37 not 38 (343997)

40 exp Alcohol Drinking/ (69105)

41 exp Alcoholic Intoxication/ (12465)

42 exp Alcoholic Beverages/ (20153)

43 exp Drinking Behavior/ (75582)

44 (beer or wine$ or cider or alcopop$ or spirit or spirits).ti,ab. (34819)

45 alcohol$.ti,ab. (332909)

46 (drink$ adj2 (binge or excessive or harm$ or heavy or misus$ or abus$ or consum$)).ti,ab. (19215)

47 (intoxicat$ or inebriat$ or drunk$).ti,ab. (50448)

48 40 or 41 or 42 or 43 or 44 or 45 or 46 or 47 (427873)

49 (comment or editorial or letter).pt. (1872653)

50 48 not 49 (416257)

51 Lifestyle/ (56608)

52 (lifestyle adj2 (intervention$ or program$ or chang$ or modif$ or improv$ or enhanc$)).ti,ab. (25897)

53 (life style adj2 (intervention$ or program$ or chang$ or modif$ or improv$ or enhanc$)).ti,ab. (2100)

54 (health adj2 (intervention$ or program$ or chang$ or modif$ or improv$ or enhanc$)).ti,ab. (131784)

55 (behavior$ change adj (intervention$ or program$)).ti,ab. (1059)

56 (behaviour$ change adj (intervention$ or program$)).ti,ab. (755)

57 (multiple risk factor adj2 (program$ or intervention$)).ti,ab. (427)

58 (multifactorial lifestyle adj (intervention$ or program$)).ti,ab. (15)

59 (health behavior$ adj (program$ or intervention$)).ti,ab. (266)

60 (health behaviour$ adj (program$ or intervention$)).ti,ab. (83)

61 multiple health behavior$ change intervention$.ti,ab. (25)

62 multiple health behaviour$ change intervention$.ti,ab. (9)

63 multiple behavior$ risk factor$ intervention$.ti,ab. (1)

64 multiple behaviour$ risk factor$ intervention$.ti,ab. (0)

65 multiple behavior$ risk factor$ program$.ti,ab. (0)

66 multiple behaviour$ risk factor$ program$.ti,ab. (0)

67 multiple risk behaviour$ intervention$.ti,ab. (4)

68 multiple risk behavior$ intervention$.ti,ab. (5)

69 multiple risk behaviour$ program$.ti,ab. (0)

70 multiple risk behavior$ program$.ti,ab. (0)

71 51 or 52 or 53 or 54 or 55 or 56 or 57 or 58 or 59 or 60 or 61 or 62 or 63 or 64 or 65 or 66 or 67 or 68 or 69 or 70 (204608)

72 7 and 19 (3)

73 7 and 32 (24)

74 7 and 39 (9)

75 7 and 50 (20)

76 7 and 71 (42)

77 72 or 73 or 74 or 75 or 76 (86)

78 systematic review.pt. (132480)

79 systematic$ review$.ti,ab. (180672)

80 meta-analysis as topic/ (18162)

81 meta-analytic$.ti,ab. (7359)

82 meta-analysis.ti,ab,pt. (179101)

83 metanalysis.ti,ab. (248)

84 metaanalysis.ti,ab. (1614)

85 meta analysis.ti,ab. (152064)

86 meta-synthesis.ti,ab. (903)

87 metasynthesis.ti,ab. (336)

88 meta synthesis.ti,ab. (903)

89 meta-regression.ti,ab. (7974)

90 metaregression.ti,ab. (691)

91 meta regression.ti,ab. (7974)

92 (synthes$ adj3 literature).ti,ab. (3635)

93 (synthes$ adj3 evidence).ti,ab. (10972)

94 integrative review.ti,ab. (3093)

95 data synthesis.ti,ab. (11419)

96 (research synthesis or narrative synthesis).ti,ab. (3414)

97 (systematic study or systematic studies).ti,ab. (12292)

98 (systematic comparison$ or systematic overview$).ti,ab. (3446)

99 evidence based review.ti,ab. (2065)

100 comprehensive review.ti,ab. (15568)

101 critical review.ti,ab. (15920)

102 quantitative review.ti,ab. (680)

103 structured review.ti,ab. (844)

104 realist review.ti,ab. (324)

105 realist synthesis.ti,ab. (216)

106 or/78-105 (367183)

107 review.pt. (2678738)

108 medline.ab. (118672)

109 pubmed.ab. (120843)

110 cochrane.ab. (85441)

111 embase.ab. (94046)

112 cinahl.ab. (28256)

113 psyc?lit.ab. (917)

114 psyc?info.ab. (36835)

115 (literature adj3 search$).ab. (62504)

116 (database$ adj3 search$).ab. (62874)

117 (bibliographic adj3 search$).ab. (2670)

118 (electronic adj3 search$).ab. (22912)

119 (electronic adj3 database$).ab. (30552)

120 (computeri?ed adj3 search$).ab. (3586)

121 (internet adj3 search$).ab. (3343)

122 included studies.ab. (25111)

123 (inclusion adj3 studies).ab. (16987)

124 inclusion criteria.ab. (88577)

125 selection criteria.ab. (30591)

126 predefined criteria.ab. (1986)

127 predetermined criteria.ab. (1071)

128 (assess$ adj3 (quality or validity)).ab. (81999)

129 (select$ adj3 (study or studies)).ab. (68325)

130 (data adj3 extract$).ab. (65294)

131 extracted data.ab. (14776)

132 (data adj2 abstracted).ab. (5400)

133 (data adj3 abstraction).ab. (1757)

134 published intervention$.ab. (187)

135 ((study or studies) adj2 evaluat$).ab. (190525)

136 (intervention$ adj2 evaluat$).ab. (11649)

137 confidence interval$.ab. (423731)

138 heterogeneity.ab. (169655)

139 pooled.ab. (92122)

140 pooling.ab. (12585)

141 odds ratio$.ab. (276087)

142 (Jadad or coding).ab. (185924)

143 or/108-142 (1488635)

144 107 and 143 (248198)

145 review.ti. (484674)

146 145 and 143 (153791)

147 (review$ adj4 (papers or trials or studies or evidence or intervention$ or evaluation$)).ti,ab. (191900)

148 106 or 144 or 146 or 147 (594849)

149 letter.pt. (1093364)

150 editorial.pt. (537535)

151 comment.pt. (863708)

152 149 or 150 or 151 (1872653)

153 148 not 152 (580670)

154 77 and 153 (4)

155 limit 154 to yr="2009 -Current" (2)

**Embase strategy**

Database: Embase <1974 to 2020 August 06>

Search Strategy:

--------------------------------------------------------------------------------

1 (care adj4 leave$).ti,ab. (962)

2 looked after child$.ti,ab. (189)

3 looked-after child$.ti,ab. (189)

4 Foster Home Care/ or Residential Facilities/ (10693)

5 Transition to Adult Care/ (1923)

6 4 and 5 (13)

7 1 or 2 or 3 or 6 (1152)

8 exp Obesity/ (519818)

9 exp Body Weight Gain/ or exp Body Weight Loss/ (55162)

10 (obese or obesity or overweight).ti,bt,ab. (464553)

11 (healthy adj2 (diet$ or eating)).ti,bt,ab. (18637)

12 exp Diet/ (322661)

13 (fruit$ adj2 (intake or consum$ or increase or portion$ or serving$ or frequenc$ or number$ or preference$ or choice$ or choos$)).ti,bt,ab. (12807)

14 (vegetable$ adj2 (intake or consum$ or increase or portion$ or serving$ or frequenc$ or number$ or preference$ or choice$ or choos$)).ti,bt,ab. (13131)

15 "5 a day".ti,bt,ab. (236)

16 "five a day".ti,bt,ab. (67)

17 ((food or diet$) adj (choice$ or frequenc$ or intake)).ti,bt,ab. (106917)

18 Feeding Behavior/ (82129)

19 Food preference/ (13482)

20 exp diet therapy/ or exp diet/ (608381)

21 8 or 9 or 10 or 11 or 12 or 13 or 14 or 15 or 16 or 17 or 18 or 19 or 20 (1258508)

22 (editorial or letter).pt. (1792459)

23 21 not 22 (1203957)

24 (physical adj2 (fit$ or train$ or activ$ or endur$)).ti,bt,ab. (172663)

25 (exercis$ adj2 (fit$ or train$ or activ$ or endur$)).ti,bt,ab. (43611)

26 ((promot$ or uptak$ or encourag$ or increas$ or start$ or adher$) adj2 (exercis$ or gym$ or sport$ or fitness)).ti,bt,ab. (32676)

27 ((decreas$ or reduc$ or discourag$) adj2 (sedentary or deskbound)).ti,bt,ab. (1793)

28 (sport$ or walk$ or running or jogging or bicycling or biking or swimming).ti,bt,ab. (358229)

29 (active adj (travel$ or transport$ or commut$)).ti,bt,ab. (9637)

30 Fitness/ (37136)

31 exp Recreation/ or exp Sport/ (234284)

32 exp kinesiotherapy/ or exp exercise/ (383874)

33 running/ or jogging/ or swimming/ or walking/ (117617)

34 24 or 25 or 26 or 27 or 28 or 29 or 30 or 31 or 32 or 33 (925423)

35 (letter or editorial).pt. (1792459)

36 34 not 35 (896160)

37 exp smoking/ (387723)

38 (smoking or antismoking or anti-smoking).ti,bt,ab. (321431)

39 (smoker or smokers).ti,bt,ab. (133647)

40 tobacco/ or tobacco.ti,bt,ab. (129322)

41 37 or 38 or 39 or 40 (542679)

42 (letter or editorial).pt. (1792459)

43 41 not 42 (519513)

44 exp Drinking Behavior/ (48629)

45 exp Alcoholic Intoxication/ (12211)

46 exp Alcoholic Beverages/ (29596)

47 exp Drinking Behavior/ (48629)

48 (beer or wine$ or cider or alcopop$ or spirit or spirits).ti,bt,ab. (41873)

49 alcohol$.ti,bt,ab. (451176)

50 (drink$ adj2 (binge or excessive or harm$ or heavy or misus$ or abus$ or consum$)).ti,bt,ab. (26939)

51 (intoxicat$ or inebriat$ or drunk$).ti,bt,ab. (63374)

52 44 or 45 or 46 or 48 or 49 or 50 or 51 (557105)

53 (editorial or letter).pt. (1792459)

54 52 not 53 (546756)

55 Lifestyle/ (113222)

56 (lifestyle adj2 (intervention$ or program$ or chang$ or modif$ or improv$ or enhanc$)).ti,bt,ab. (38131)

57 (life style adj2 (intervention$ or program$ or chang$ or modif$ or improv$ or enhanc$)).ti,bt,ab. (3992)

58 (health adj2 (intervention$ or program$ or chang$ or modif$ or improv$ or enhanc$)).ti,bt,ab. (159238)

59 (behavior$ change adj (intervention$ or program$)).ti,bt,ab. (1165)

60 (behaviour$ change adj (intervention$ or program$)).ti,bt,ab. (968)

61 (health behavior$ adj (program$ or intervention$)).ti,bt,ab. (287)

62 (health behaviour$ adj (program$ or intervention$)).ti,bt,ab. (100)

63 55 or 56 or 57 or 58 or 59 or 60 or 61 or 62 (293760)

64 7 and 23 (20)

65 7 and 36 (46)

66 7 and 43 (21)

67 7 and 54 (27)

68 7 and 63 (57)

69 64 or 65 or 66 or 67 or 68 (144)

70 systematic$ review$.ti,ab. (224486)

71 systematic$ literature review$.ti,ab. (16392)

72 "systematic review"/ (256142)

73 "systematic review (topic)"/ (25096)

74 meta analysis/ (192942)

75 "meta analysis (topic)"/ (42581)

76 meta-analytic$.ti,ab. (8648)

77 meta-analysis.ti,ab. (198261)

78 metanalysis.ti,ab. (653)

79 metaanalysis.ti,ab. (8144)

80 meta analysis.ti,ab. (198261)

81 meta-synthesis.ti,ab. (996)

82 metasynthesis.ti,ab. (393)

83 meta synthesis.ti,ab. (996)

84 meta-regression.ti,ab. (10020)

85 metaregression.ti,ab. (1114)

86 meta regression.ti,ab. (10020)

87 (synthes$ adj3 literature).ti,ab. (4201)

88 (synthes$ adj3 evidence).ti,ab. (12240)

89 (synthes$ adj2 qualitative).ti,ab. (3394)

90 integrative review.ti,ab. (3029)

91 data synthesis.ti,ab. (13659)

92 (research synthesis or narrative synthesis).ti,ab. (3778)

93 (systematic study or systematic studies).ti,ab. (13350)

94 (systematic comparison$ or systematic overview$).ti,ab. (3853)

95 (systematic adj2 search$).ti,ab. (34605)

96 systematic$ literature research$.ti,ab. (369)

97 (review adj3 scientific literature).ti,ab. (1935)

98 (literature review adj2 side effect$).ti,ab. (16)

99 (literature review adj2 adverse effect$).ti,ab. (4)

100 (literature review adj2 adverse event$).ti,ab. (16)

101 (evidence-based adj2 review).ti,ab. (3895)

102 comprehensive review.ti,ab. (17856)

103 critical review.ti,ab. (17169)

104 critical analysis.ti,ab. (8478)

105 quantitative review.ti,ab. (790)

106 structured review.ti,ab. (1141)

107 realist review.ti,ab. (349)

108 realist synthesis.ti,ab. (213)

109 (pooled adj2 analysis).ti,ab. (21694)

110 (pooled data adj6 (studies or trials)).ti,ab. (3113)

111 (medline and (inclusion adj3 criteria)).ti,ab. (27125)

112 (search adj (strateg$ or term$)).ti,ab. (39649)

113 or/70-112 (590399)

114 medline.ab. (147457)

115 pubmed.ab. (153352)

116 cochrane.ab. (110708)

117 embase.ab. (119091)

118 cinahl.ab. (32983)

119 psyc?lit.ab. (999)

120 psyc?info.ab. (33520)

121 lilacs.ab. (8292)

122 (literature adj3 search$).ab. (79625)

123 (database$ adj3 search$).ab. (79093)

124 (bibliographic adj3 search$).ab. (3151)

125 (electronic adj3 search$).ab. (28078)

126 (electronic adj3 database$).ab. (41093)

127 (computeri?ed adj3 search$).ab. (4328)

128 (internet adj3 search$).ab. (4505)

129 included studies.ab. (31644)

130 (inclusion adj3 studies).ab. (21187)

131 inclusion criteria.ab. (153837)

132 selection criteria.ab. (37156)

133 predefined criteria.ab. (2727)

134 predetermined criteria.ab. (1367)

135 (assess$ adj3 (quality or validity)).ab. (109665)

136 (select$ adj3 (study or studies)).ab. (89920)

137 (data adj3 extract$).ab. (90023)

138 extracted data.ab. (19498)

139 (data adj2 abstracted).ab. (9125)

140 (data adj3 abstraction).ab. (2617)

141 published intervention$.ab. (230)

142 ((study or studies) adj2 evaluat$).ab. (274774)

143 (intervention$ adj2 evaluat$).ab. (16325)

144 confidence interval$.ab. (514512)

145 heterogeneity.ab. (219736)

146 pooled.ab. (130441)

147 pooling.ab. (16869)

148 odds ratio$.ab. (350170)

149 (Jadad or coding).ab. (222573)

150 evidence-based.ti,ab. (149145)

151 or/114-150 (2092342)

152 review.pt. (2605265)

153 151 and 152 (263627)

154 review.ti. (552415)

155 151 and 154 (191072)

156 (review$ adj10 (papers or trials or trial data or studies or evidence or intervention$ or evaluation$ or outcome$ or findings)).ti,ab. (569259)

157 (retriev$ adj10 (papers or trials or studies or evidence or intervention$ or evaluation$ or outcome$ or findings)).ti,ab. (30855)

158 113 or 153 or 155 or 156 or 157 (1079721)

159 letter.pt. (1130530)

160 editorial.pt. (661929)

161 159 or 160 (1792459)

162 158 not 161 (1058867)

163 (animal/ or nonhuman/) not exp human/ (5725152)

164 162 not 163 (1022248)

165 69 and 164 (14)

166 limit 165 to yr="2009 -Current" (11)

**Health Systems Evidence** and **Epistemonikos** were searched using “care leaver” and 2 further records were identified.

**1.6 Epistemonikos searches**

A series of searches were conducted using Epistemonikos (https://www.epistemonikos.org/). Results were downloaded and deduplicated in EndNote software.

31st July 2020

(title:((title:(refugee OR asylum) OR abstract:(refugee OR asylum)) AND (title:(food OR diet) OR abstract:(food OR diet))) OR abstract:((title:(refugee OR asylum) OR abstract:(refugee OR asylum)) AND (title:(food OR diet) OR abstract:(food OR diet)))) 6 records

(title:(refugee OR asylum) OR abstract:(refugee OR asylum)) AND (title:(alcohol) OR abstract:(alcohol)) 1 record

(title:(refugees OR asylum) OR abstract:(refugees OR asylum)) AND (title:(smoking OR tobacco) OR abstract:(smoking OR tobacco)) 1 record

(title:(refugees OR asylum) OR abstract:(refugees OR asylum)) AND (title:(exercise) OR abstract:(exercise)) 0 records

(title:(traveller OR roma OR gypsy OR gypsies) OR abstract:(traveller OR roma OR gypsy OR gypsies)) AND (title:(food OR diet) OR abstract:(food OR diet)) 0 records

(title:(traveller OR roma OR gypsy OR gypsies) OR abstract:(traveller OR roma OR gypsy OR gypsies)) AND (title:(alcohol) OR abstract:(alcohol))

(title:(traveller OR roma OR gypsy OR gypsies) OR abstract:(traveller OR roma OR gypsy OR gypsies)) AND (title:(smoking) OR abstract:(smoking)) 1 records

(title:(traveller OR roma OR gypsy OR gypsies) OR abstract:(traveller OR roma OR gypsy OR gypsies)) AND (title:(exercise) OR abstract:(exercise)) 1 record

title:(unemployed OR employment OR workless OR "job seeker") AND (title:(food OR diet OR alcohol OR smoking OR tobacco OR cigarette OR exercise) OR abstract:(food OR diet OR alcohol OR smoking OR tobacco OR cigarette OR exercise)) 5 records

**1.7 Health Systems Evidence searches**

A series of searches were conducted using Health Systems Evidence (<https://www.healthsystemsevidence.org/?lang=en>).

All searches combined with the "implementation" filter

5^th^ August 2020

Search 1

roma or travellers or gypsies 1 records

Search 2

refugee or asylum 2 records

Search 3

employed 0 records

Search 4

employment 0 records

Search 5

unemployed 3 records

Search 6

Unemployment 9 records

Search 7

Jobless 0 records

Search 8

Workless 0 records

**Appendix 2: Characteristics of intervention reviews (by disadvantaged group)**

**Table A1 Low income or socio-economic status (SES)**

| **Review (and other groups/ behaviours targeted)** | **Intervention and Comparator** | **Outcomes** | **Setting and countries** | **Study designs & number of studies** |
| --- | --- | --- | --- | --- |
| **1. Targeted interventions** | | | | |
| **1.1 Tobacco use** | | | | |
| **1.1.1 Systematic reviews** | | | | |
| Boland (2018)^1^ ^a^  Low SES  + Homeless; Prisoners; Other (mental illness, substance misusers, indigenous/native persons, at-risk youth, disability or chronic illness) | **Intervention** Individual level (Technology-based smoking cessation interventions. Included websites, computer programmes, DVD, video-telephony and text messages.)  **Comparator** Care as usual/ no intervention Active control | Smoking or tobacco use *(Smoking cessation)* | Technology-based  **Countries eligible** Any country  **Countries included** UK, USA, New Zealand | **Study designs eligible** Experimental or quasi-experimental  **Study designs included** Experimental or quasi-experimental  **Number of studies included** 13 (11/13 targeted disadvantaged group; 4/13 low SES; 0/13 prisoners; 0/13 homeless) |
| Bryant (2011)^2^  Low income  +Homeless; Prisoners; Other (mental illness, indigenous/native persons, at-risk youth) | **Intervention** Individual level (Behavioural smoking cessation interventions: brief advice, incentives for quitting, self-help interventions or behavioural support)  **Comparator** Care as usual/ no intervention Other intervention | Smoking or tobacco use *(Smoking abstinence at ≥ 6 months follow-up, short-term abstinence at ≤ 3 months)* | Any  **Countries eligible** OECD/ high income/developed countries  **Countries included** UK, USA, Australia, New Zealand | **Study designs eligible** Experimental or quasi-experimental  **Study designs included** Experimental or quasi-experimental  **Number of studies included** 32 (12/32 low income; 1/32 homeless; 1/32 prisoners) |
| Bull (2014)^3^  Low income  + Diet; physical inactivity | **Intervention** Individual level (Any interventions targeting behaviour change. Ranged from provision of tailored self-help materials to individual counselling/ group programmes.)  **Comparator** Care as usual/ no intervention Other intervention | Physical activity *(Behavioural outcomes relevant to physical activity)* Diet *(Behavioural outcomes relevant to healthy eating)* Smoking or tobacco use *(Behavioural outcomes relevant to smoking cessation)* | Any  **Countries eligible** Any country  **Countries included** UK, USA, Australia, South/central America | **Study designs eligible** Experimental or quasi-experimental  **Study designs included** Experimental or quasi-experimental  **Number of studies included** 35 |
| Ford (2013)^4^ ^a^  Low SES  + Homeless; Prisoners; Other (indigenous/native persons; pregnancy) | **Intervention** Individual level (Smoking cessation interventions utilising peer or partner support)  **Comparator** None/not applicable Care as usual/ no intervention Active control Other intervention | Smoking or tobacco use *(Abstinence)* | Any  **Countries eligible** Any country  **Countries included** UK, USA | **Study designs eligible** Not reported  **Study designs included** Experimental or quasi-experimental  **Number of studies included** 8 (0/8 homeless; 0/8 prisoners; 7/8 low SES) |
| Kock (2019)^5^  Low SES | **Intervention** Individual level (Behavioural smoking cessation interventions delivered to individuals [not groups], e.g. in-person/telephone support, digital behavioural support, financial incentives, brief interventions, motivational interviewing, written materials)  **Comparator** Care as usual/ no intervention Waitlist control Active control | Smoking or tobacco use *(Smoking cessation at ≥6 months follow-up)* | Any  **Countries eligible** Any country  **Countries included** UK, Rest of Europe, USA, Australia, Asia | **Study designs eligible** Experimental or quasi-experimental  **Study designs included** Experimental or quasi-experimental  **Number of studies included** 42 (26/42 tailored for low SES; other interventions universal with differential effects reported) |
| Michie (2009)^6^  Low income  + Diet; physical inactivity | **Intervention** Individual level (Any intervention. Included e.g. education, counselling, peer support, tailored information, lottery/contests, exercise vouchers, self-help materials) Community/workplace level (Community-wide programmes, including e.g. classes, billboards, health fairs, door-to-door campaign, contests, menu labelling)  **Comparator** Any | Physical activity Diet Smoking or tobacco use | Any  **Countries eligible** Any country  **Countries included** UK, Rest of Europe, USA, Canada | **Study designs eligible** Experimental or quasi-experimental  **Study designs included** Experimental or quasi-experimental  **Number of studies included** 13 |
| Murray (2009)^7^ ^b^  Low income  +Homeless; People with learning disabilities; Other (mental illness; ethnic minorities; pregnancy; lone parents; institutionalised people) | **Intervention** Individual level (Interventions to find and support adult smokers. Focus on improving access to services. Cessation interventions included e.g. behavioural support, NHS stop smoking services, nicotine replacement therapy, brief advice, educational materials, incentives)  **Comparator** Any | Smoking or tobacco use *(including cessation rates and quit attempts)* Process outcomes | Smoking cessation services  **Countries eligible** Any country  **Countries included** UK, Rest of Europe, USA (not fully reported) N/A for systematic reviews | **Study designs eligible** Experimental or quasi-experimental, Observational, Qualitative, Systematic reviews  **Study designs included** Experimental or quasi-experimental, Observational, Qualitative, Systematic reviews  **Number of studies included** 48 (Unclear if any on homeless or people with learning disabilities) |
| O'Mara-Eves (2015)^8,9^  Low SES  + Other (ethnic minorities; disadvantage defined by PROGRESS-Plus & other ways in which people may be systematically disadvantaged by discrimination)  + Diet; physical inactivity; alcohol; other (drug misuse, any Marmot priority area) | **Intervention** Individual level (Community engagement interventions. Unclear if all at individual level or not).  **Comparator** Care as usual/ no intervention Waitlist control Active control Other intervention  Other (Inactive control, matched data from target population, other/unclear) | *All behaviours pooled in main analysis. Sensitivity analyses explored effect on specific behaviours.*  Physical activity Diet Alcohol use Smoking or tobacco use  Other behavioural outcomes Intermediate outcomes Physical health Other (Social support in relation to the health behaviours; Community outcomes; Engagee outcomes) | Any  **Countries eligible** OECD/ high income/developed countries  **Countries included** UK, USA, Canada (not fully reported) | **Study designs eligible** Experimental or quasi-experimental  **Study designs included** Experimental or quasi-experimental  **Number of studies included** 131 (34/131 low SES + unspecified number on low SES combined with other PROGRESS factor) |
| Smith (2019)^10^  Low income + SES | **Intervention** Individual level (Behavioural smoking cessation interventions.^c^ Included interventions covered nicotine replacement therapy, behavioural counselling and financial incentives.)  **Comparator** Any | Smoking or tobacco use *(Smoking abstinence)* Intermediate outcomes | Any  **Countries eligible** OECD/ high income/developed countries  **Countries included** UK, Rest of Europe, Canada | **Study designs eligible** Experimental or quasi-experimental, Observational  **Study designs included** Experimental or quasi-experimental, Observational  **Number of studies included** 11 (5/11 targeted; other interventions universal with differential effects reported) |
| Smith (2020)^11^  Low SES | **Intervention** Individual level (GP brief interventions, Stop smoking services [SSS], innovative GP and SSS-based interventions, e.g. ﬁnancial incentive schemes)  **Comparator** Any | Smoking or tobacco use Process outcomes | UK specialist stop smoking services or primary care  **Countries eligible** UK  **Countries included** UK | **Study designs eligible** Experimental or quasi-experimental, Observational  **Study designs included** Experimental or quasi-experimental, Observational  **Number of studies included** 30 (4/30 targeted interventions) |
| Stiehl (2018)^12^  Low income  + Diet; physical inactivity; other (any health promotion intervention) | **Intervention** Individual level (Any health promotion programme for low-wage employees. Included e.g. training/education, incentives, health promotion delivered with occupational health and safety initiatives)  Community/workplace level (e.g. fresh fruit provision, worksite changes)  **Comparator** Any | Physical activity Diet *(e.g. fruit & vegetable consumption/purchases)* Smoking or tobacco use *(e.g. smoking cessation)* Other behavioural outcomes Intermediate outcomes Physical health Process outcomes Other | Workplaces (or in a community- based organisation if the focus is on low-wage employees)  **Countries eligible** USA  **Countries included** USA | **Study designs eligible** Experimental or quasi-experimental, Observational, Qualitative  **Study designs included** Experimental or quasi-experimental, Observational, Qualitative  **Number of studies included** 35 (15/35 intervention studies; unclear how many targeted diet/PA/smoking) |
| Walton-Moss (2014)^13^  Low SES  + Disadvantaged areas or communities; Other (ethnic minorities; low literacy; rural areas)  + Diet; physical inactivity | **Intervention** Individual level (Community-based cardiovascular disease interventions e.g. education, counselling & support, exercise classes, community improvement and food provision) Community/workplace level  **Comparator** Any | Physical activity Diet *(Dietary results relevant to the AHA dietary score)* Smoking or tobacco use *(Smoking status)* Physical health | Community  **Countries eligible** Any country  **Countries included** Rest of Europe, USA, Australia, Asia, Africa, South/central America | **Study designs eligible** Not reported  **Study designs included** Experimental or quasi-experimental  **Number of studies included** 32 (9/32 low income or disadvantaged area) |
| Wilson (2017)^14^  Low SES  + Homeless; Prisoners; Other (mental illness, indigenous/native persons, at-risk youth) | **Intervention** Individual level (e.g. motivational interviewing, CBT-based counselling, access to telephone quitline, mindfulness training, financial incentives)  **Comparator** Any | Smoking or tobacco use *(Abstinence/quit rates)* | Any  **Countries eligible** OECD/ high income/developed countries  **Countries included** UK, Rest of Europe, USA, Australia | **Study designs eligible** Experimental or quasi-experimental  **Study designs included** Experimental or quasi-experimental  **Number of studies included** 24 (9/24 low SES; 1/24 homeless; 2/24 prisoners) |
| **1.1.2. Systematic review protocols** | | | | |
| Burke (2019)^15^  Low income  + Unemployed; Disadvantaged areas or communities; Other (ethnic minorities) | **Intervention** Individual level (Any individual-level smoking cessation intervention, e.g. brief advice, pharmacotherapy, behavioural support)  **Comparator** Care as usual/ no intervention | Smoking or tobacco use *(Biochemically verified smoking abstinence, point-prevalence and continuous abstinence, self-reported abstinence, other smoking behaviour)*  Process outcomes | Any  **Countries eligible** Any country | **Study designs eligible** Experimental or quasi-experimental |
| **1.2. Excessive alcohol use** | | | | |
| **1.2.1 Systematic reviews** | | | | |
| Muckle (2012)^16^  Low income  + Homeless; Other (mental illness, substance misusers, family history of alcohol addiction, family dysfunction, foetal alcohol spectrum disorder, past trauma) | **Intervention** Individual level (Managed alcohol programmes: harm reduction initiatives that treat the alcohol abuse of vulnerable people by serving controlled amounts of alcohol on a daily schedule)  **Comparator** None/not applicable Care as usual/ no intervention Other intervention (Moderate drinking, brief intervention, 12-step programmes) | Alcohol use *(Reduction in drinking & binge drinking; abstinence)* Other behavioural outcomes Physical health | Any  **Countries eligible** Any country  **Countries included** No relevant studies found | **Study designs eligible** Experimental or quasi-experimental  **Number of studies included** 0 (empty review) |
| O'Mara-Eves (2015) | See Table A1 section 1.1.1. (low income/SES: tobacco use) | | | |
| **1.3. Physical activity** | | | | |
| **1.3.1 Systematic reviews** | | | | |
| Bull (2014) | See Table A1 section 1.1.1. (low income/SES: tobacco use) | | | |
| Cleland (2013)^17^  Low income & SES  + Unemployed; Disadvantaged areas or communities | **Intervention** Individual level (Any PA intervention [individually, socially, environmentally or policy targeted]. Included e.g. behavioural counselling, education, structured exercise, self-monitoring, tailored messages) Community/workplace level (Community-wide programmes)  **Comparator** Any | Physical activity *(Any outcome measure, or outcome closely related to physical activity such as cardiorespiratory fitness. Primary outcome was any continuous measure of PA. If not available, the proportions of participants meeting a specified level of physical activity)* | Any  **Countries eligible** Any country  **Countries included** Rest of Europe, USA, Australia, Asia, South/central America | **Study designs eligible** Experimental or quasi-experimental  **Study designs included** Experimental or quasi-experimental  **Number of studies included** 19 |
| Everson-Hock (2013)^18^  Low SES  + Diet | **Intervention** Individual level (Community-based interventions. Included e.g. education, food skills/cooking, counselling, exercise vouchers, access to internet portal, empowerment) Community/workplace level (Included physical activity community awareness campaign, community fruit & veg intervention, building community networks) Physical environment (Opening of food retail outlets)  **Comparator** None/not applicable Care as usual/ no intervention Active control | Physical activity Diet *(Fruit and vegetable intake, consumption of high fat foods, other eating habits)* Intermediate outcomes Physical health Mental health and wellbeing | Community  **Countries eligible** UK  **Countries included** UK | **Study designs eligible** Experimental or quasi-experimental, Observational, Qualitative  **Study designs included** Experimental or quasi-experimental, Observational, Qualitative  **Number of studies included** 35 |
| Michie (2009) | See Table A1 section 1.1.1. (low income/SES: tobacco use) | | | |
| O'Mara-Eves (2015) | See Table A1 section 1.1.1. (low income/SES: tobacco use) | | | |
| Olstad (2017)^19^  Low income & SES  + Disadvantaged areas or communities  + Diet | **Intervention** Any targeted policy eligible.  Individual level (Provision of information/education, implemented as 'policy') Community/workplace level (Organizational policies in disadvantaged schools) Physical environment (Changes to the built environment) Food subsidies (Fruit and vegetable subsidies)  **Comparator** Care as usual/ no intervention | Physical activity *(including greater frequency, duration or intensity across any domain [e.g. leisure, transport, occupational])* Diet *(including increased consumption/purchasing of fruit, vegetables, water or products with government-sanctioned health logos or symbols, reduced intake/purchasing of energy, energy-dense foods, processed foods, fast food, snack foods, sweets, confectionary, sugar-sweetened beverages or competitive foods [i.e. foods sold in schools outside of US school-based meal programs]; reduced portion size, improved overall diet quality)* Physical health | Policies (any level of government and non-government initiatives described as policies, e.g. in schools)  **Countries eligible** OECD/ high income/developed countries  **Countries included** UK, Rest of Europe, USA, New Zealand | **Study designs eligible** Experimental or quasi-experimental, Observational  **Study designs included** Experimental or quasi-experimental, Observational  **Number of studies included** 18 |
| Stiehl (2018) | See Table A1 section 1.1.1. (low income/SES: tobacco use) | | | |
| Stormacq (2020)^20^  Low income & SES  + Other (social/socio-economic disadvantage defined by PROGRESS factors)   + Diet; Other (any) | **Intervention** Individual level (Health literacy interventions at individual, community or societal levels [no societal-level interventions found]). Community/workplace level  **Comparator** None/not applicable Care as usual/ no intervention Active control | Physical activity Diet Other behavioural outcomes Intermediate outcomes Physical health Mental health and wellbeing Quality of life Health service utilisation | Clinical or community settings  **Countries eligible** Any country  **Countries included** USA, Asia, Africa | **Study designs eligible** Experimental or quasi-experimental  **Study designs included** Experimental or quasi-experimental  **Number of studies included** 21 (12/21 low income or SES) |
| Walton-Moss (2014) | See Table A1 section 1.1.1. (low income/SES: tobacco use) | | | |
| **1.4. Unhealthy diet** | | | | |
| **1.4.1. Systematic reviews** | | | | |
| An (2019)^21^  Low income | **Intervention** Individual level (Nutrition education, client-choice intervention, food display intervention, diabetes management intervention)  **Comparator** Any | Diet *(e.g. food choices, fruit, vegetable & wholegrain consumption, use of fats and salts, diabetes self-management behaviours)*  Intermediate outcomes Physical health Other (Food security) | Food pantries  **Countries eligible** USA  **Countries included** USA | **Study designs eligible** Experimental or quasi-experimental  **Study designs included** Experimental or quasi-experimental   **Number of studies included** 14 |
| Black (2012)^22^ ^a^  Low SES  + Disadvantaged areas or communities; Other (indigenous/native persons; ethnic minorities) | **Intervention** Food subsidies (Any direct or indirect strategy to reduce the price of food including policy initiatives, transport and infrastructure subsidies, cross-subsidies. Programmes had to have provided a 10% or greater reduction in the price of targeted foods)  **Comparator** None  Care as usual/ no intervention Waitlist control Active control | Diet *(Nutritional intake/food purchases, including fruit & vegetable intake)* Physical health Health service utilisation Adverse events or unintended effects | Community  **Countries eligible** OECD/ high income/developed countries  **Countries included** UK, USA, New Zealand | **Study designs eligible** Experimental or quasi-experimental  **Study designs included** Experimental or quasi-experimental  **Number of studies included**  14 |
| Bull (2014)^3^ | See Table A1 section 1.1.1. (low income/SES: tobacco use) | | | |
| De Marchis (2019)^23^  Low income | **Intervention** Individual level (Food insecurity interventions: education and/or referral, food or food voucher provision)  **Comparator** Any | Diet *(Fruit and vegetable intake)* Intermediate outcomes Physical health Health service utilisation Process outcomes Other (Food security status) | Healthcare settings  **Countries eligible** USA  **Countries included** USA | **Study designs eligible** Not reported  **Study designs included** Experimental or quasi-experimental, Observational, Qualitative  **Number of studies included** 23 |
| Eicher-Miller (2020)^24^  Low income | **Intervention** Individual level (Interventions to improve food security, diet, or health, e.g. nutrition education, referrals, connection to caseworker/counsellor/health monitoring, client choice, supplementary foods)  **Comparator** Any | Diet *(Dietary quality [ Healthy Eating Index], dietary intake [24-h dietary recall])* Physical health Mental health and wellbeing Other (Food insecurity) | Food pantries  **Countries eligible**  USA  **Countries included**  USA | **Study designs eligible** Not reported  **Study designs included** Experimental or quasi-experimental, Observational (not fully reported)  **Number of studies included** 15 (2/15 diet intervention studies) |
| Engel (2020)^25^  Low income | **Intervention** Food subsidies **(**Fruit and vegetable incentive programs for Supplemental Nutrition Assistance Program [SNAP] participants- mainly vouchers/tokens/coupons)  **Comparator** Any | Diet (*Fruit and vegetable purchases or consumption)* | Retail venues (mainly farmers' markets)  **Countries eligible** USA  **Countries included** USA | **Study designs eligible** Experimental or quasi-experimental  **Study designs included** Experimental or quasi-experimental  **Number of studies included**  19 |
| Everson-Hock (2013) | See Table A1 section 1.3.1. (low income/SES: physical inactivity) | | | |
| Hsiao (2019) ^26^  Low income^d^ | **Intervention** Physical environment (Mobile produce markets or portable fruit and vegetable markets)  **Comparator** Any | Diet *(Fruit and/or vegetable intake)* | Mobile produce markets  **Countries eligible** USA  **Countries included** USA | **Study designs eligible** Experimental or quasi-experimental, Observational Qualitative, Modelling studies  **Study designs included** Experimental or quasi-experimental, Observational, Qualitative, Modelling studies  **Number of studies included** 24 |
| Iacovou (2013)^27^  Low income^d^  + Other (indigenous/native persons) | **Intervention** Community/workplace level (Community kitchens: community-focused and -initiated cooking-type programmes)  **Comparator** Any | Diet *(Intake of nutritious food, variety in food intake, diversity of fruit and vegetables purchased, frequency of fast-food consumption, % consuming 5 portions fruit and vegetables)*  Intermediate outcomes Other (Food security, dignity, access to community services and resources, social interactions, skills and/or support) | Community kitchens  **Countries eligible** Any country  **Countries included** UK, Australia, Canada | **Study designs eligible** Experimental or quasi-experimental, Observational, Qualitative, Systematic reviews  **Study designs included** Observational, Qualitative  **Number of studies included** 10 |
| Langellier (2013) ^28^  Low income^d^ | **Intervention** Physical environment (Corner store conversion: partnering with small ‘corner’ food stores to expand access to high-quality healthy food. Strategies included social marketing and nutrition education)  **Comparator** Any | Diet *(Overall sales of healthy food or individual-level purchases of health foods, consumption of (un)healthy foods, healthfulness of meal preparation)* Intermediate outcomes | Corner stores  **Countries eligible** Any country  **Countries included** USA, Canada, Other (Marshall Islands) | **Study designs eligible** Not reported  **Study designs included** Experimental or quasi-experimental  **Number of studies included** 18 (10/18 intervention studies) |
| Long (2019)^29^  Low income (food pantry/bank users)  + Other (prevention/management of specific chronic diseases) | **Intervention** Individual level (Any intervention focused on disease prevention or management [e.g. diabetes, obesity, infectious diseases]. Interventions relevant to this review involved education plus providing healthy foods, or solely providing healthy foods)  **Comparator** None/not applicable Care as usual/ no intervention Other intervention | Diet *(Intake of specific foods, e.g. vegetables, fruit, dietary fibre, dairy & plant-based meals, fatty foods, money spent on unhealthy foods, e.g. meats, carbonated beverages, desserts)* Alcohol use *(Binge drinking)* Smoking or tobacco use Other behavioural outcomes Intermediate outcomes Physical health Mental health and wellbeing Quality of life Health service utilisation Other (Food insecurity, diabetes distress) | Food pantries or food banks  **Countries eligible** Any country  **Countries included** USA | **Study designs eligible** Experimental or quasi-experimental, Observational  **Study designs included** Experimental or quasi-experimental  **Number of studies included** 6 (2/6 targeted diet in a non-clinical population) |
| Michie (2009) | See Table A1 section 1.1.1. (low income/SES: tobacco use) | | | |
| O'Mara-Eves (2015) | See Table A1 section 1.1.1. (low income/SES: tobacco use) | | | |
| Ohly (2017)^30^  Low income | **Intervention** Food subsidies (Food vouchers from the [UK] Healthy Start or the [USA] Special Supplemental Nutrition Program for Women, Infants and Children [WIC] programmes)  **Comparator** None/not applicable | Diet *(Nutritional benefits)* Other (Financial assistance; Vouchers handed over to others) | Healthy Start or WIC food voucher programmes  **Countries eligible** UK, USA  **Countries included** UK, USA | **Study designs eligible** Experimental or quasi-experimental, Observational, Qualitative  **Study designs included** Observational, Qualitative  **Number of studies included** 38 |
| Olstad (2017) | See Table A1 section 1.3.1. (low income/SES: physical inactivity) | | | |
| Stiehl (2018) | See Table A1 section 1.1.1. (low income/SES: tobacco use) | | | |
| Stormacq (2020) | See Table A1 section 1.3.1. (low income/SES: physical inactivity) | | | |
| Verghese (2019)^31^  Low income | **Intervention** Individual level (Interventions that targeted SNAP beneficiaries: monetary incentives, nutrition education, or combined education + incentives) Food subsidies  **Comparator** Any | Diet *(Food intake [fruits, vegetables, refined grains, whole grains, sugar, sugar sweetened beverages; dietary fat], food insecurity)* Intermediate outcomes | US SNAP  **Countries eligible**  USA  **Countries included** USA | **Study designs eligible** Not reported  **Study designs included** Experimental or quasi-experimental, Observational  **Number of studies included** 12 (16 articles) |
| Walton-Moss (2014) | See Table A1 section 1.1.1. (low income/SES: tobacco use) | | | |
| Zhang (2020)^32^  Low income | **Intervention** Food subsidies (WIC programme after 2009 revision [including fruit & vegetable vouchers], farmers' markets/other fruit & vegetable interventions associated with WIC)  **Comparator** Any | Diet (*Fruit and vegetable consumption or purchase)* | WIC programme  **Countries eligible** USA  **Countries included** USA | **Study designs eligible** Not reported  **Study designs included** Experimental or quasi-experimental, Observational, Qualitative (not fully reported)  **Number of studies included** 39 |
| **2. Targeted and population-level interventions** | | | | |
| **2.1. Tobacco use** | | | | |
| **2.1.1. Systematic reviews** | | | | |
| Guillaumier (2012)^33^ ^a^  Low income & SES  + Homeless; Other (mental illness, indigenous/native persons, ethnic minorities) | **Intervention** Media campaigns  **Comparator** Any | Smoking or tobacco use *(Cessation)* Intermediate outcomes | Mass media campaigns  **Countries eligible** OECD/ high income/developed countries  **Countries included** USA, Australia, New Zealand | **Study designs eligible** Experimental or quasi-experimental, Observational  **Study designs included** Experimental or quasi-experimental, Observational  **Number of studies included** 17 (11/17 low income or SES; 0/17 homeless) |
| Hill (2014)^34^ ^b^  Low SES | **Intervention** Individual level (Smoking cessation support: pharmacotherapy and/or behavioural or motivational support) Community/workplace level (Community-level programmes combining ≥ 2 other intervention types) Fiscal measures (Price increases) Media campaigns Smoking bans Advertising controls (Advertising bans, warning labels [no studies found])  **Comparator** Any | Smoking or tobacco use *(Unclear but included smoking prevalence, quit ratios, quit attempts, quit rates)* Intermediate outcomes Physical health Engagement with services | Any  **Countries eligible** Countries at an advanced stage of the tobacco epidemic  **Countries included** UK, Rest of Europe, USA, Australia, Canada, New Zealand (not fully reported) | **Study designs eligible** Experimental or quasi-experimental, Observational, Qualitative, Systematic reviews  **Study designs included** Experimental or quasi-experimental, Observational, Qualitative, Systematic reviews  **Number of studies included** 84 |
| **2.2. Physical inactivity** | | | | |
| **2.2.1. Reviews of systematic reviews** | | | | |
| Craike (2018)^35^  Low income & SES | **Intervention** Individual level (Any intervention [including policies] with PA change objective. Included e.g. education, structured exercise, counselling, tailored feedback etc.) Community/workplace level Other policy (Targeted/universal policy in general- specific policies not reported)  **Comparator** None/not applicable | Physical activity | Any  **Countries eligible** Any country  **Countries included** N/A | **Study designs eligible** Systematic reviews  **Study designs included** Systematic reviews  **Number of studies included** 17 |
| **3. Population-level interventions** | | | | |
| **3.1. Tobacco use** | | | | |
| **3.1.1. Systematic reviews** | | | | |
| Bader (2011)^36^  Low SES  + Other (mental illness; substance misusers; indigenous/native persons; children & young people; young adults; heavy/long-term smokers) | **Intervention** Fiscal measures (Tobacco taxation and pricing)  **Comparator** None/not applicable | Smoking or tobacco use (*Smoking initiation, cessation, stages of uptake, prevalence, consumption [quantity])* | Taxes and pricing  **Countries eligible** Unclear (focus on high-income but some studies from middle-income)  **Countries included** UK, Rest of Europe, USA, Australia, Canada, New Zealand, Asia, South/central America (not fully reported) | **Study designs eligible** Not reported  **Study designs included** Not reported  **Number of studies included** 108 (25/108 low SES) |
| Beauchamp (2010)^37^  Low SES | **Intervention** Individual level (Subsidised nicotine replacement therapy) Fiscal measures (Increased cigarette prices through raised taxation) | Smoking or tobacco use *(Rates of smoking prevalence or consumption)* | Any  **Countries eligible** Any country  **Countries included** UK, Rest of Europe, USA, Australia, Canada, New Zealand, Asia, Africa | **Study designs eligible** Not reported  **Study designs included** Experimental or quasi-experimental, Observational  **Number of studies included** 49 (29/49 conducted in healthy populations) |
| Bell (2009)^38^  Low SES  + Other (ethnicity; gender) | **Intervention** Smoking bans (indoor workplaces and hospitality settings)  **Comparator** None/not applicable | Smoking or tobacco use *(Consumption of cigarettes, other smoking outcomes, including smoking prevalence)* Physical health Adverse events or unintended effects | Workplaces and hospitality settings  **Countries eligible** Any country  **Countries included** UK, Rest of Europe, USA, Australia | **Study designs eligible** Not reported  **Study designs included** Experimental or quasi-experimental, Observational  **Number of studies included** 16 |
| Brown (2014a)^39^  Low SES | **Intervention** Any ‘population-level’ intervention eligible.  Individual level (Individual cessation support services with national coverage) Community/workplace level (Settings-based interventions) Fiscal measures Media campaigns (including promoting the use of quitlines and nicotine replacement products) Smoking bans (including voluntary, regional or partial) Advertising controls (including warning labels) Other policy (Multiple policy interventions, New Deal for Communities)  **Comparator** Any | Smoking or tobacco use *(Quit attempts, smoking prevalence)* Intermediate outcomes Physical health Engagement with services | Population-level  **Countries eligible** Europe and non-European countries at stage 4 of the tobacco epidemic  **Countries included** UK, Rest of Europe, USA, Australia, Canada, New Zealand | **Study designs eligible** Experimental or quasi-experimental, Observational, Qualitative  **Study designs included** Experimental or quasi-experimental, Observational, Qualitative  **Number of studies included** 117 |
| Brown (2014b)^40^  Low SES | **Intervention**.  Individual level (cessation support and smoke-free homes interventions) Community/workplace level (school-based prevention)  Any policy eligible.  Fiscal measures (Price/tax increases) Media campaigns Smoking bans Advertising controls Controls on access to tobacco, alcohol or unhealthy food Other policy (Multiple policies [e.g. Finnish Tobacco Control Act- smoke free, age-of-sale and health warnings])  **Comparator** Any | Smoking or tobacco use *(including intentions/attitudes/ perceptions, smoking behaviour, initiation, relapse and cessation rates, smoking prevalence)* Intermediate outcomes Physical health Environment | Any  **Countries eligible** Europe and non-European countries at stage 4 of the tobacco epidemic  **Countries included** UK, Rest of Europe, USA, Australia, Canada, New Zealand, Asia | **Study designs eligible** Experimental or quasi-experimental, Observational, Qualitative  **Study designs included** Experimental or quasi-experimental, Observational  **Number of studies included** 38 |
| Durkin (2012)^41^ ^b^  Low SES | **Intervention** Media campaigns  **Comparator** Any | Smoking or tobacco use *(Quitline calls, smoking-related activity and behaviour)* Intermediate outcomes | Mass-media campaigns  **Countries eligible** Any country  **Countries included** Rest of Europe, USA, Australia, Canada, New Zealand, South/central America | **Study designs eligible** Not reported  **Study designs included** Experimental or quasi-experimental, Observational, Systematic reviews  **Number of studies included** 26 |
| Mozaffarian (2012)^42^ ^b^  Low income & SES  + Diet; physical inactivity | **Intervention** Community/workplace level (Workplace incentives for individuals, school and workplace-based approaches) Physical environment (Physical activity labelling/information, financial incentives to influence shop locations, local environmental change) Media campaigns Smoking bans Advertising controls (including cigarette warnings)  Controls on access to tobacco, alcohol or unhealthy food Food subsidies Menu/food labelling Other policy (Agricultural policy)  **Comparator** Any | Physical activity Diet Smoking or tobacco use Intermediate outcomes Physical health | Population-level approaches (organisational [e.g. schools, workplaces] community, regional/state, or national levels)  **Countries eligible** Any country  **Countries included** Not reported | **Study designs eligible** Experimental or quasi-experimental, Observational, Modelling studies, Systematic reviews  **Study designs included** Experimental or quasi-experimental, Observational, Modelling studies, Systematic reviews  **Number of studies included** Not reported |
| **3.1.2. Reviews of systematic reviews** | | | | |
| Jepson (2010)^43^  Low SES  +Diet; physical inactivity; alcohol; other (drug misuse, sexual risk behaviours) | **Intervention** Individual level (behavioural or educational component) Community/workplace level (Community-level and school-based interventions, smoke-free policies) Physical environment (Supermarket interventions) Media campaigns Controls on access to tobacco, alcohol or unhealthy food (Reducing tobacco sales to minors) Other policy (Interventions to reduce drink driving)  **Comparator** None/not applicable | Physical activity *(Prevention of health problems related to sedentary lifestyle, increased uptake of exercise, increase in exercise levels)* Diet *(Fruit and vegetable intake, changes in shopping habits)* Alcohol use *(Prevent/reduce alcohol consumption, prevent/reduce drink driving, promote moderate drinking)* Smoking or tobacco use *(Smoking cessation, smoking prevention)* Other behavioural outcomes | Any, except secondary or tertiary care  **Countries eligible** Any country  **Countries included** N/A | **Study designs eligible** Systematic reviews  **Study designs included** Systematic reviews  **Number of studies included** 103 |
| Lorenc (2013)^44^  Low SES  + Diet; physical inactivity; other (any health status or behaviour) | **Intervention** Any intervention outside healthcare system eligible. Individual level (Printed materials on folic acid) Community/workplace level (Community-based physical activity or smoking interventions, school-based physical activity/diet or smoking interventions, free fruit in schools) Fiscal measures (Tobacco price increases) Media campaigns Smoking bans (Workplace smoking bans) Advertising controls Controls on access to tobacco, alcohol or unhealthy food (Restrictions on tobacco sales to minors) Other policy (Free folic acid supplements)  **Comparator** None/not applicable | Physical activity Diet *(Folic acid intake, healthy eating behaviours)* Smoking or tobacco use *(Smoking behaviour, smoking-related outcomes)* Other behavioural outcomes Intermediate outcomes Other ('Various health status outcomes') | Any  **Countries eligible** OECD/ high income/developed countries  **Countries included** N/A | **Study designs eligible** Systematic reviews  **Study designs included** Systematic reviews  **Number of studies included** 12 reviews (9/12 focused on diet/PA/smoking) |
| Stead (2019)^45^ ^e^  Low SES  + Diet; physical inactivity; alcohol; other (drug misuse, sexual and reproductive health) | **Intervention** Media campaigns  **Comparator** None/not applicable | Physical activity Diet Alcohol use Smoking or tobacco use Other behavioural outcomes Intermediate outcomes Engagement with services | Mass media campaigns  **Countries eligible** Any country  **Countries included** UK, Rest of Europe, USA, Australia, Canada, New Zealand, Asia, N/A for systematic reviews | **Study designs eligible** Experimental or quasi-experimental, Observational, Systematic reviews  **Study designs included** Experimental or quasi-experimental, Observational, Systematic reviews  **Number of studies included** 36 reviews in review of reviews; 24 primary studies in systematic review |
| Thomson (2018)^46^  Low SES  + Diet; alcohol (& covers 5 other policy domains, e.g. workplace regulations) | **Intervention** Fiscal measures Media campaigns Smoking bans Advertising controls Food subsidies Product interventions Menu/food labelling Other policy  **Comparator** Any | Diet Alcohol use Smoking or tobacco use Physical health | Policies  **Countries eligible** OECD/ high income/developed countries  **Countries included** N/A | **Study designs eligible** Systematic reviews  **Study designs included** Systematic reviews  **Number of studies included** 29 |
| **3.2. Alcohol use** | | | | |
| **3.2.1. Reviews of systematic reviews** | | | | |
| Jepson (2010) | See Table A1 section 3.1.2. (low income/SES: tobacco use) | | | |
| Stead (2019) | See Table A1 section 3.1.2. (low income/SES: tobacco use) | | | |
| Thomson (2018) | See Table A1 section 3.1.2. (low income/SES: tobacco use) | | | |
| **3.3. Physical inactivity** | | | | |
| **3.3.1. Systematic reviews** | | | | |
| Baker (2015a)^47^  Low income & SES  + Disadvantaged areas or communities | **Intervention** Community/workplace level (community-wide approaches, utilising ≥2 of: social marketing; other communication strategies; individual counselling; working with voluntary, government and non-government organisations; working within specific settings e.g. schools, workplaces; environmental change strategies)  **Comparator** Any | Physical activity *(Dichotomous [% physically active/ active during leisure time/ inactive] or continuous [leisure time physical activity time spent, walking time spent, energy expenditure as metabolic equivalents])* Adverse events or unintended effects | Geographically-defined communities  **Countries eligible** Any country  **Countries included** UK, Rest of Europe, USA, Australia, Canada, Asia | **Study designs eligible** Experimental or quasi-experimental  **Study designs included** Experimental or quasi-experimental  **Number of studies included** 33 |
| Boelsen-Robinson (2015)^48^  Low SES  + Diet | **Intervention** Community/workplace level (Multicomponent whole-of-community interventions)  **Comparator** Any | Physical activity *(Stages of change in physical activity)* Diet *(Dietary fat, saturated fatty acid intake, "dietary factors")* Physical health | 'Whole-of-community' interventions  **Countries eligible** Any country  **Countries included** UK, Rest of Europe, USA, Australia | **Study designs eligible** Experimental or quasi-experimental, Observational, Qualitative  **Study designs included** Observational  **Number of studies included** 12 |
| Olstad (2016)^49^  Low SES  + Diet | **Intervention** Any universal policy eligible  Community/workplace level (Local policies; Girl Scout policies) Physical environment (Land use and transportation plans) Fiscal measures (Unhealthy food/drink taxes, children's fitness tax credit) Media campaigns Menu/food labelling Other policy (National or state/regional school policies, French national nutrition and public health programme, Finnish national diabetes prevention programme, free bus pass for older adults)  **Comparator** Any | Physical activity *(including greater frequency, duration or intensity across any domain of physical activity [e.g. leisure, transport, occupation])* Diet *(food consumption and food purchasing [increased consumption of fruit, vegetables, products with government-sanctioned health logos or symbols, breast milk, reduced intake of energy including energy-dense foods, processed foods, fast food, snack foods, sweets, confectionary, sugar-sweetened beverages, carbonated beverages, caloric beverages, competitive food [i.e. foods sold outside of US-based school meal programmes]; reduced portion size; improved overall diet quality])* Physical health | Policies (any level of government and non-government initiatives described as policies, e.g. in schools)  **Countries eligible** Any country  **Countries included** UK, Rest of Europe, USA, Australia, Canada, Asia | **Study designs eligible** Experimental or quasi-experimental, Observational  **Study designs included** Experimental or quasi-experimental, Observational  **Number of studies included** 36 |
| Smith (2017)^50^  Low SES  + Other (ethnicity) | **Intervention** Physical environment (Built environment interventions or changes occurring at the individual, neighbourhood or town level)  **Comparator** Any | Physical activity *(total PA, moderate PA, moderate-to-vigorous PA, recreational PA, active transport, total walking, total cycling, walking or cycling for transport, visitation to or use of a setting)* | Built environment  **Countries eligible** Any country  **Countries included** UK, Rest of Europe, USA, Australia, New Zealand | **Study designs eligible** Experimental or quasi-experimental, Observational  **Study designs included** Experimental or quasi-experimental, Observational  **Number of studies included** 28 |
| Thomas (2018)^51^  Low SES | **Intervention** Media campaigns  **Comparator** None/not applicable | Physical activity Intermediate outcomes | Mass media campaigns  **Countries eligible** OECD/ high income/developed countries  **Countries included** UK, Rest of Europe, USA, Australia, Canada | **Study designs eligible** Experimental or quasi-experimental, Observational  **Study designs included** Experimental or quasi-experimental, Observational  **Number of studies included** 23 papers; 17 campaigns |
| **3.3.2. Review protocols** | | | | |
| Baker (2015b)^52^ | **Intervention** Unclear ("Any public health intervention")  **Comparator** Care as usual/ no intervention Waitlist control Active control Other | Physical activity *(Proportion of the population achieving moderate to vigorous physical activity, duration of physical activity*) Physical health Adverse events or unintended effects  Other (Sedentary behaviour/TV viewing) | Any  **Countries eligible** Any country | **Study designs eligible** Systematic reviews |
| Tully (2013)^53^  Low SES | **Intervention** Physical environment (Built environment interventions)  **Comparator** Any | Physical activity *(Changes in total PA level [transformed to minutes MVPA if data allows], changes in domain-specific PA levels [active travel or recreational]).* Physical health Mental health and wellbeing Quality of life | Built environment  **Countries eligible** Any country | **Study designs eligible** Experimental or quasi-experimental, Observational |
| **3.3.3. Reviews of systematic reviews** | | | | |
| Jepson (2010) | See Table A1 section 3.1.2. (low income/SES: tobacco use) | | | |
| Lorenc (2013) | See Table A1 section 3.1.2. (low income/SES: tobacco use) | | | |
| Stead (2019) | See Table A1 section 3.1.2. (low income/SES: tobacco use) | | | |
| **3.4. Unhealthy diet** | | | | |
| **3.4.1. Systematic reviews** | | | | |
| Backholer (2016)^54^  Low SES | **Intervention** Fiscal measures (Change in sugar-sweetened beverage price)  **Comparator** None/not applicable | Diet *(Sugar sweetened beverage purchase or consumption, energy intake)* Physical health | Taxes  **Countries eligible** OECD/ high income/developed countries  **Countries included** UK, Rest of Europe, USA, Australia, New Zealand | **Study designs eligible** Not reported  **Study designs included** Observational, Modelling studies  **Number of studies included** 11 |
| Hillier-Brown (2017)^55^  Low SES | **Intervention** Interventions to restrict, guide or enable choice or provide information.  Individual level (Personalised receipts) Product interventions (Changing pre-packed children’s meal content, trans-fat law) Menu/food labelling (Calorie labelling, signposting (un)healthier choices) Other policy (Food outlet award schemes, price decreases/increases for healthier/unhealthier choices, incentives for healthier choices, telemarketing, personalised receipts)  **Comparator** Any | Diet *(e.g. energy intake)*  Other behavioural outcomes Intermediate outcomes Process outcomes | Food outlets that as their main business sold ready-to-eat meals and were openly accessible to the general public  **Countries eligible** Any country  **Countries included** UK, USA, Australia | **Study designs eligible** Experimental or quasi-experimental  **Study designs included** Experimental or quasi-experimental  **Number of studies included** 30 |
| Lobstein (2020)^56^ ^b^  Low income & SES | **Intervention** Fiscal measures (Sugar-sweetened beverage taxes) Advertising controls (Restrictions on advertising to children) Menu/food labelling (Front-of-pack nutrition labelling)  **Comparator** None/not applicable Other intervention (modelled alternative interventions) | Diet *(Consumption or purchase of unhealthy food etc.)*  Intermediate outcomes  Physical health  Other (Cost implications, acceptability) | Policies  **Countries eligible** Any country  **Countries included** UK, Rest of Europe, USA, Australia, New Zealand, Asia, Africa, South/central America (not fully reported) | **Study designs eligible** Not reported  **Study designs included** Modelling studies, Systematic reviews (not fully reported)  **Number of studies included** 125 |
| McGill (2015)^57^  Low SES | **Intervention** Any eligible (no studies found for advertising controls, labelling, recommendations/guidance).  Individual level (Included health education or dietary counselling)  Community/workplace level (Environmental measures in specific settings) Fiscal measures (Taxes on high-energy density foods) Media campaigns Food subsidies Product interventions (Salt reformulation)  **Comparator** Any | Diet *(Dietary intake)* Physical health | Any  **Countries eligible** Any country  **Countries included** UK, Rest of Europe, USA, Australia, Canada, New Zealand | **Study designs eligible** Experimental or quasi-experimental, Observational, Modelling studies  **Study designs included** Experimental or quasi-experimental, Observational, Modelling studies  **Number of studies included** 36 |
| McLaren (2016)^58^  Low SES | **Intervention**  Sodium reduction interventions (most initiatives consisted of multiple interventions)  Community/workplace level (e.g. limits on sodium content in foods in certain environments) Fiscal measures (No studies found) Media campaigns Advertising controls Product interventions Menu/food labelling  **Comparator** Any | Diet *(Salt intake in grams per day)* | Population-level interventions in government jurisdictions (e.g. country, state/region/province)  **Countries eligible** Any country  **Countries included** UK, Rest of Europe, USA, Canada, New Zealand, Asia | **Study designs eligible** Experimental or quasi-experimental, Observational  **Study designs included** Experimental or quasi-experimental, Observational  **Number of studies included** 15 national initiatives (data from 25 published articles, 15 grey literature documents, and 13 country questionnaires) |
| Olstad (2016) | See Table A1 section 3.3.1. (low income/SES: physical inactivity) | | | |
| Pfinder (2020)^59^  Low income & SES  + Other (indigenous/native persons; disability or chronic illness; PROGRESS categories) | **Intervention** Fiscal measures (unprocessed sugar or sugar-added foods taxes)  **Comparator** None/not applicable Care as usual/ no intervention | Diet *(consumption of unprocessed sugar/ sugar-added foods, energy intake from unprocessed sugar/ sugar-added foods, total energy intake, diet composition, including consumption of untaxed sugar and sugar-added foods, difference in mean consumption of taxed sugar-added foods compared with untaxed sugar-added foods)* Physical health  Quality of life Other (expenditure, sales) | Taxes  **Countries eligible** Any country  **Countries included** Rest of Europe (Hungary) | **Study designs eligible** Experimental or quasi-experimental, Observational  **Study designs included** Experimental or quasi-experimental  **Number of studies included** 1 |
| Sarink (2016)^60^  Low SES | **Intervention** Menu/food labelling (Menu energy labelling)  **Comparator** Any | Diet *(Calories purchased or consumed)* Intermediate outcomes Physical health | Restaurants/ food outlets  **Countries eligible** Any country  **Countries included** USA (natural experiments) / Not reported (experimental studies) | **Study designs eligible** Experimental or quasi-experimental, Observational, Qualitative  **Study designs included** Experimental or quasi-experimental, Observational, Qualitative  **Number of studies included** 14 |
| Thow (2014)^61^  Low income | **Intervention** Fiscal measures Food subsidies  **Comparator** None/not applicable | Diet *(Food/nutrient consumption)* | Taxes/subsidies  **Countries eligible** Any country  **Countries included** UK, Rest of Europe, USA, Australia, New Zealand, South/central America | **Study designs eligible** Experimental or quasi-experimental, Observational, Modelling studies  **Study designs included** Experimental or quasi-experimental, Modelling studies  **Number of studies included** 38 |
| von Philipsborn (2019)^62^  Low SES  + Disadvantaged areas or communities | **Intervention** Physical environment (Interventions that alter the physical or social environment in which individuals make beverage choices, excluding taxes)  **Comparator** Any | Diet *(Direct and indirect measures of sugar-sweetened beverages [SSB] intake, consumption of beverages other than SSB)* Physical health Adverse events or unintended effects Other (Measures of financial and economic viability, diet-related psychosocial variables, target group perceptions) | Environmental interventions, e.g. schools, workplaces, shops, restaurants  **Countries eligible** Any country  **Countries included** UK, Rest of Europe, USA, Australia, Canada, New Zealand, Asia, South/central America | **Study designs eligible** Experimental or quasi-experimental  **Study designs included** Experimental or quasi-experimental  **Number of studies included** 58 |
| **3.4.2. Review protocols** | | | | |
| Vargas-Garcia (2015)^63^  Low SES  + Disadvantaged areas or communities | **Intervention** Unclear (Interventions addressing a reduction in SSB or increase in water consumption)  **Comparator** Any | Diet *(Change in SSB or water consumption [millilitres/per day])* Intermediate outcomes Environment Other (Changes in health inequalities measured by interactions between socio-demographic characteristics and effects/outcomes) | Community settings (or clinical locations if participants had been allocated into groups by random methods)  **Countries eligible** Any country | **Study designs eligible** Experimental or quasi-experimental |
| **3.4.3. Reviews of systematic reviews** | | | | |
| Jepson (2010) | See Table A1 section 3.1.2. (low income/SES: tobacco use) | | | |
| Lorenc (2013) | See Table A1 section 3.1.2. (low income/SES: tobacco use) | | | |
| Stead (2019) | See Table A1 section 3.1.2. (low income/SES: tobacco use) | | | |
| Thomson (2018) | See Table A1 section 3.1.2. (low income/SES: tobacco use) | | | |

*AHA: American Heart Association; CBT: cognitive behavioural therapy; GP: General practitioner; MVPA: moderate to vigorous physical activity; N/A: Not applicable; NHS: National Health Service; OECD: Organisation for Economic Co-operation and Development; PA: physical activity; PROGRESS: place of residence, race/ethnicity/culture/language, occupation, gender/sex, religion, education, socioeconomic status, and social capital; SES: socio-economic status; SNAP: Supplemental Nutrition Assistance Program; SSB: sugar-sweetened beverages; SSS: stop smoking services; WIC: Special Supplemental Nutrition Program for Women, Infants and Children*

^a^ Findings are not synthesised/reported separately for each specific disadvantaged group.

^b^ Review of primary studies and reviews.

^c^ Focuses on behavioural elements of interventions rather than overall effects.
^d^ Low income/SES not specified in inclusion criteria but framed as an intervention for disadvantaged areas or low income populations.

^e^ Systematic review of primary studies of alcohol campaigns (no differential effects reported) and review of reviews on six behaviours (differential effects reported).

**Table A2 Disadvantaged areas or communities**

| **Review (and other groups/ behaviours targeted)** | **Intervention and Comparator** | **Outcomes and measures** | **Setting and country** | **Study designs** |
| --- | --- | --- | --- | --- |
| **1. Targeted interventions** | | | | |
| **1.1. Tobacco use** | | | | |
| **1.1.1. Systematic reviews** | | | | |
| Walton-Moss (2014) | See Table A1 section 1.1.1. (low income/SES: tobacco use) | | | |
| **1.1.2. Systematic review protocols** | | | | |
| Burke (2019) | See Table A1 section 1.1.2. (low income/SES: tobacco use) | | | |
| **1.2. Physical inactivity** | | | | |
| **1.2.1. Systematic reviews** | | | | |
| Cleland (2012)^64^ | **Intervention** Individual level (Interventions aiming to increase physical activity as main objective or within a comprehensive intervention) Community/workplace level  **Comparator** Any | Physical activity *(Any measure of free-living physical activity)* | Any  **Countries eligible** Any country  **Countries included** UK, Rest of Europe, USA, Africa, South/central America | **Study designs eligible** Experimental or quasi-experimental, Observational  **Study designs included** Experimental or quasi-experimental  **Number of studies included** 27 |
| Cleland (2013) | See Table A1 section 1.3.1. (low income/SES: physical inactivity) | | | |
| Olstad (2017) | See Table A1 section 1.3.1. (low income/SES: physical inactivity) | | | |
| Walton-Moss (2014) | See Table A1 section 1.1.1. (low income/SES: tobacco use) | | | |
| **1.3. Unhealthy diet** | | | | |
| **1.3.1. Systematic reviews** | | | | |
| Black (2012) | See Table A1 section 1.4.1. (low income/SES: unhealthy diet) | | | |
| Hollis-Hansen (2019)^65^ | **Intervention** Physical environment (Introduction of new food retail into a lower-income community)  **Comparator** None/not applicable | Diet *(Fruit and vegetable intake)* Process outcomes | Food retail (Community Supported Agriculture, farmers’ market, farm stand, mobile produce market, healthy corner store, or grocery store)  **Countries eligible** USA  **Countries included** USA | **Study designs eligible** Not reported  **Study designs included** Experimental or quasi-experimental, Observational  **Number of studies included** 15 |
| Olstad (2017) | See Table A1 section 1.3.1. (low income/SES: physical inactivity) | | | |
| Walton-Moss (2014) | See Table A1 section 1.1.1. (low income/SES: tobacco use) | | | |
| **2. Population-level interventions** | | | | |
| **2.1. Physical inactivity** | | | | |
| **2.1.1. Systematic reviews** | | | | |
| Baker (2015a) | See Table A1 section 3.3.1. (low income/SES: physical inactivity) | | | |
| **2.2. Unhealthy diet** | | | | |
| **2.2.1. Systematic reviews** | | | | |
| von Philipsborn (2019) | See Table A1 section 3.4.1. (low income/SES: unhealthy diet) | | | |
| **2.2.2. Review protocols** | | | | |
| Vargas-Garcia (2015) | See Table A1 section 3.4.2. (low income/SES: unhealthy diet) | | | |

*SES: socio-economic status*

**Table A3 Unemployed people**

| **Review (and other groups/ behaviours targeted)** | **Intervention and Comparator** | **Outcomes and measures** | **Setting and country** | **Study designs** |
| --- | --- | --- | --- | --- |
| **1. Targeted interventions** | | | | |
| **1.1. Tobacco use** | | | | |
| **1.1.1. Systematic reviews** | | | | |
| Hollederer (2019)^66^  **+** Diet; physical inactivity; alcohol; other (any health promotion) | **Intervention** Individual level (Health promotion interventions. Including e.g. CBT training, employment programmes, health/behaviour counselling)  **Comparator** Any | Physical activity Diet Alcohol use Smoking or tobacco use Intermediate outcomes  Physical health Mental health and wellbeing Quality of life Other (Job search outcomes, labour market integration) | Active labour market measure, course, job centre, employment agency, social enterprises for unemployed, employment centre, self-help, communities, healthy environments, counselling service etc.  **Countries eligible** Any country  **Countries included** UK, Rest of Europe, USA, Australia | **Study designs eligible** Experimental or quasi-experimental  **Study designs included** Experimental or quasi-experimental  **Number of studies included** Unclear (characteristics of 14 controlled studies reported but further uncontrolled studies included in narrative synthesis) |
| **1.1.2. Systematic review protocols** | | | | |
| Burke (2019) | See Table A1 section 1.1.2. (low income/SES: tobacco use) | | | |
| **1.2. Alcohol use** | | | | |
| **1.2.1. Systematic reviews** | | | | |
| Hollederer (2019) | See Table A3 section 1.1. (unemployed: tobacco use) | | | |
| **1.3. Physical inactivity** | | | | |
| **1.3.1. Systematic reviews** | | | | |
| Cleland (2013) | See Table A1 section 1.3.1. (low income/SES: physical inactivity) | | | |
| Hollederer (2019) | See Table A3 section 1.1. (unemployed: tobacco use) | | | |
| **1.4. Unhealthy diet** | | | | |
| **1.4.1. Systematic reviews** | | | | |
| Hollederer (2019) | See Table A3 section 1.1. (unemployed: tobacco use) | | | |

*CBT: cognitive behavioural therapy; SES: socio-economic status* All reviews are systematic reviews.

**Table A4 Homeless people**

| **Review (and other groups/ behaviours targeted)** | **Intervention and Comparator** | **Outcomes and measures** | **Setting and country** | **Study designs** |
| --- | --- | --- | --- | --- |
| **1. Targeted interventions** | | | | |
| **1.1. Tobacco use** | | | | |
| **1.1.1. Systematic reviews** | | | | |
| Boland (2018) | See Table A1 section 1.1.1. (low income/SES: tobacco use). No studies on homeless people | | | |
| Bryant (2011) | See Table A1 section 1.1.1. (low income/SES: tobacco use). | | | |
| Ford (2013) | See Table A1 section 1.1.1. (low income/SES: tobacco use). No studies on homeless people | | | |
| Gentry (2019)^67^ ^a^  + Prisoners; Other (mental illness, substance misusers, other stages of criminal justice system) | **Intervention** Individual level (E-cigarettes, including interventions with free provision etc.)  **Comparator** Care as usual/ no intervention Waitlist control Other intervention (Another type of nicotine or non-nicotine e-cigarette, smoking cessation intervention [e.g. nicotine replacement therapy, behavioural intervention]) | Smoking or tobacco use *(Smoking cessation at longest follow-up, smoking reduction at longest follow-up)* Adverse events or unintended effects Engagement with services Other (Perceived barriers and facilitators to e-cigarette use; health economics outcomes) | Any  **Countries eligible** Any country  **Countries included** Rest of Europe, USA, Australia, New Zealand, other (1 international online study) | **Study designs eligible** Experimental or quasi-experimental, Observational, Qualitative  **Study designs included** Experimental or quasi-experimental, Observational, Qualitative  **Number of studies included** 9 (0/9 prisoners; 2/9 homeless [on barrier/facilitators not interventions]) |
| Murray (2009) | See Table A1 section 1.1.1. (low income/SES: tobacco use). Unclear if any studies on homeless people. | | | |
| Wilson (2017) | See Table A1 section 1.1.1. (low income/SES: tobacco use). | | | |
| **1.2. Alcohol use** | | | | |
| **1.2.1. Systematic reviews** | | | | |
| Muckle (2012) | See Table A1 section 1.2.1. (low income/SES: alcohol use) | | | |
| **1.3. Unhealthy diet** | | | | |
| **1.3.1. Systematic reviews** | | | | |
| Ijaz (2018)^68^  Problem drinkers experiencing homelessness | **Intervention** Individual level (Interventions to improve nutritional status, or macro- or micro-nutrient deficiencies: education, information or support, supplements or food provision or multi-component)  **Comparator** Any | Diet *(Nutrition status or deficiencies [including intake, e.g. fruit and vegetable intake])* Alcohol use *(Drinking behaviour)* Physical health Mental health and wellbeing Quality of life Process outcomes Other (Cognitive function) | Any  **Countries eligible** Any country  **Countries included** UK, Rest of Europe, USA, Australia, Canada, South/central America | **Study designs eligible** Experimental or quasi-experimental, Observational  **Study designs included** Experimental or quasi-experimental, Observational  **Number of studies included** 25 |
| **2. Targeted and population-level interventions** | | | | |
| **2.1. Tobacco use** | | | | |
| **2.1.1. Systematic reviews** | | | | |
| Guillaumier (2012) | See Table A1 section 2.1.1. (low income/SES: tobacco use). No studies on homeless people | | | |

*SES: Socio-economic status*

All reviews are systematic reviews.

^a^ Findings are not synthesised/reported separately for each specific disadvantaged group.

**Table A5 Prisoners**

| **Review (and other groups/ behaviours targeted)** | **Intervention and Comparator** | **Outcomes and measures** | **Setting and country** | **Study designs** |
| --- | --- | --- | --- | --- |
| **1. Targeted interventions** | | | | |
| **1.1. Tobacco use** | | | | |
| **1.1.1. Systematic reviews** | | | | |
| Boland (2018) | See Table A1 section 1.1.1. (low income/SES: tobacco use). No studies on prisoners | | | |
| Bryant (2011) | See Table A1 section 1.1.1. (low income/SES: tobacco use). | | | |
| de Andrade (2016)^69^ | **Intervention** Individual level (Smoking cessation programmes. Including motivational interviewing and/or cognitive–behaviour therapy, pharmacological support or multicomponent cessation programme). Community/workplace level (Prison smoking bans)  **Comparator** Any | Smoking or tobacco use *(Smoking cessation outcomes [including reduction in use, abstinence])* Other behavioural outcomes Other (Any behavioural outcomes attributed to the intervention.) | Prisons  **Countries eligible** Any country  **Countries included** UK, Rest of Europe, USA, Australia, Canada, Asia | **Study designs eligible** Experimental or quasi-experimental, Observational  **Study designs included** Experimental or quasi-experimental, Observational  **Number of studies included** 20 |
| Ford (2013) | See Table A1 section 1.1.1. (low income/SES: tobacco use). No studies on prisoners | | | |
| Frazer (2016)^70^  +Other (healthcare settings, higher education) | **Intervention** Community/workplace level (Partial or complete indoor smoking ban or policy in the specified settings)  **Comparator** Any | Smoking or tobacco use *(Active smoking outcomes [including reported smoking rates, evidence of smoking cessation or quit attempts]).* Physical health Adverse events or unintended effects | Specialist settings, including healthcare facilities, higher education, and correctional facilities (prisons and military institutions)  **Countries eligible** Any country  **Countries included** Rest of Europe, USA, Australia, Canada, Asia | **Study designs eligible** Experimental or quasi-experimental, Observational  **Study designs included**  Experimental or quasi-experimental, Observational  **Number of studies included** 17 (3/17 in prisons) |
| Gentry (2019) | See Table A4 section 1.1.1 (homeless people: tobacco use). No studies for prisoners | | | |
| Mohan (2018)^71^  + Diet; physical inactivity | **Intervention** Individual level (Structured physical activity, nutrition interventions, mixed interventions combining physical activity and education sessions, smoking cessation)  **Comparator** Any | Diet Smoking or tobacco use *(Smoking status)* Physical health | Correctional settings  **Countries eligible** Any country  **Countries included** Rest of Europe, USA, Australia, Canada | **Study designs eligible** Experimental or quasi-experimental, Observational  **Study designs included** Experimental or quasi-experimental  **Number of studies included** 11 |
| Puljevic (2019)^72^ ^a^ | **Intervention** Individual level (Pre-release intensive behavioural intervention, education, free nicotine patches on prison entry and a Quitline telephone smoking-cessation support service)  Community/workplace level (Prison smoking ban) | Smoking or tobacco use *(Pre-release intention to smoke or remain abstinent following release, smoking relapse or abstinence following release, quit attempts following post-release smoking relapse)* | Prisons  **Countries eligible** Any country  **Countries included** USA, Australia | **Study designs eligible** Not reported  **Study designs included** Experimental or quasi-experimental, Observational, Qualitative  **Number of studies included** 15 |
| Wilson (2017) | See Table A1 section 1.1.1. (low income/SES: tobacco use). | | | |
| Wright (2011)^73 b^  + Diet; physical inactivity; alcohol; other (any health promotion: drug misuse, sexual health, HIV/hepatitis prevention included) | **Intervention** Individual level (Peer education)  **Comparator** Any | Other behavioural outcomes Intermediate outcomes | Prisons  **Countries eligible** Any country  **Countries included** USA, Asia, Africa | **Study designs eligible** Experimental or quasi-experimental, Observational, Qualitative  **Study designs included** Experimental or quasi-experimental, Qualitative  **Number of studies included** 10 (No studies found on smoking/diet/PA and no relevant alcohol studies.) |
| **1.1.2. Review protocols** | | | | |
| Almondes (2017)^74^  + Alcohol; physical inactivity; diet; other (any behaviour) | **Intervention** Individual level (Health coaching interventions)  **Comparator** Care as usual/ no intervention Other intervention | Physical activity Diet Alcohol use Smoking or tobacco use Other behavioural outcomes Intermediate outcomes Physical health Mental health and wellbeing Other (Medical adherence) | Prisons, correctional facilities or young offender institutions  **Countries eligible** Any country | **Study designs eligible** Experimental or quasi-experimental, Observational |
| **1.2. Excessive alcohol use** | | | | |
| **1.2.1. Systematic reviews** | | | | |
| Newbury-Birch (2016)^75^  + Other (other stages of criminal justice system) | **Intervention** Individual level (Up to three hours of face-to-face brief intervention)  **Comparator** Any | Alcohol use *(including AUDIT, number of drinking days, weekly units of alcohol, alcohol-related problems, drinks consumed per day/drinking day, heavy drinking days)* Other behavioural outcomes Engagement with services | Prisons, police custody, magistrates' courts, probation settings, juvenile correctional facilities  **Countries eligible** Any country  **Countries included** UK, USA | **Study designs eligible** Experimental or quasi-experimental  **Study designs included** Experimental or quasi-experimental  **Number of studies included** 10 (3/10 prisoners) |
| Newbury-Birch (2018)^76^ | **Intervention** Individual level (Brief interventions, and extended longer alcohol psychosocial interventions)  **Comparator** Care as usual/ no intervention Active control Other intervention | Alcohol use *(Various outcomes, including level of alcohol use, sobriety, addiction severity, alcohol use disorders)* Other behavioural outcomes Intermediate outcomes Mental health and wellbeing Engagement with services | Prisons  **Countries eligible** Any country  **Countries included** UK, USA | **Study designs eligible** Experimental or quasi-experimental  **Study designs included** Experimental or quasi-experimental  **Number of studies included** 9 |
| Wright (2011) | See Table A5 section 1.1.1 (prisoners: tobacco use) | | | |
| Almondes (2017) | See Table A5 section 1.1.2. (prisoners: tobacco use) | | | |
| **1.3. Physical inactivity** | | | | |
| **1.3.1. Systematic reviews** | | | | |
| Mohan (2018) | See Table A5 section 1.1.1 (prisoners: tobacco use) | | | |
| Wright (2011) | See Table A5 section 1.1.1 (prisoners: tobacco use) | | | |
| **1.3.2. Review protocols** | | | | |
| Almondes (2017) | See Table A5 section 1.1.2. (prisoners: tobacco use) | | | |
| **1.4. Unhealthy diet** | | | | |
| **1.4.1. Systematic reviews** | | | | |
| Mohan (2018) | See Table A5 section 1.1.1 (prisoners: tobacco use) | | | |
| Wright (2011) | See Table A5 section 1.1.1 (prisoners: tobacco use) | | | |
| **1.4.2. Review protocols** | | | | |
| Almondes (2017) | See Table A5 section 1.1.2. (prisoners: tobacco use) | | | |

*AUDIT: Alcohol Use Disorder Identification Test; PA: physical activity; SES: socio-economic status*

^a^ Focus on smoking after release from prison but intervention(s) delivered while in prison.

^b^ Peer health promotion interventions for any health issue eligible but no studies found on smoking, diet or physical. One intervention included alcohol/drug use content but no alcohol-related outcomes.

**Table A6 People with learning disabilities**

| **Review (and other groups/ behaviours targeted)** | **Intervention and Comparator** | **Outcomes and measures** | **Setting and countries** | **Study designs** |
| --- | --- | --- | --- | --- |
| **1. Targeted interventions** | | | | |
| **1.1. Tobacco use** | | | | |
| Kerr (2013)^77^  + Alcohol | **Intervention** Individual level (Information/advice, education, brief interventions, intensive interventions, group support, other psychological interventions, pharmacological interventions all eligible. Included e.g. educational interventions, motivational interviewing, mindfulness)  **Comparator** Any | Alcohol use *(Biological markers, self-reported consumption)* Smoking or tobacco use *(Carbon monoxide levels, cotinine levels, self-reported consumption)* Intermediate outcomes Quality of life Process outcomes | Any  **Countries eligible** Any country  **Countries included** UK, USA, Australia | **Study designs eligible** Experimental or quasi-experimental, Observational, Qualitative  **Study designs included** Experimental or quasi-experimental, Observational  **Number of studies included** 9 |
| Murray (2009) | See Table A1 section 1.1.1. (low income/SES: tobacco use). Unclear if any studies on people with learning disabilities. | | | |
| **1.2. Excessive alcohol use** | | | | |
| Kerr (2013) | See Table A6 section 1.1. (people with learning disabilities: tobacco use) | | | |
| **1.3. Physical inactivity** | | | | |
| Bondar (2020)^78^ | **Intervention** Individual level (Physical activity or sports-based interventions, e.g. exercise/sports programmes, walking interventions, water-based exercise training)  **Comparator** Any | Intermediate outcomes *(Intention, motivation and attitude regarding PA/sport participation, influence of these psychological factors on behavioural change [e.g. PA level] and quality of life)* Quality of life | Any  **Countries eligible** Any country  **Countries included** Not reported (all from high income countries) | **Study designs eligible** Experimental or quasi-experimental, Observational  **Study designs included** Experimental or quasi-experimental, Observational  **Number of studies included** 13 |
| Brooker (2015)^79^ | **Intervention** Individual level (Any intervention eligible. All included interventions were health education or promotion programmes with physical activity, nutrition and weight loss components)  **Comparator** Any | Physical activity | Any  **Countries eligible** Any country  **Countries included** UK, USA | **Study designs eligible** Experimental or quasi-experimental, Observational  **Study designs included** Experimental or quasi-experimental  **Number of studies included** 6 |
| Hassan (2019)^80^ | **Intervention** Individual level (Aim to increase PA, including structured physical activity, cycling or walking programmes and behavioural change programmes that included education and nutrition components + PA)  **Comparator** Any (Not specified but included trials had wait list, care as usual, active controls and other PA interventions) | Physical activity | Any  **Countries eligible** Any country  **Countries included** UK, Rest of Europe, USA, Australia | **Study designs eligible** Experimental or quasi-experimental  **Study designs included** Experimental or quasi-experimental  **Number of studies included** 9 |
| Temple (2017)^81^ | **Intervention** Individual level (Any PA intervention. Included multi-component lifestyle programmes, health education sessions, exercise videos and a motor-sequence learning exercise)  **Comparator** Any | Physical activity Diet  Intermediate outcomes Physical health Quality of life | Any  **Countries eligible** Any country  **Countries included** Rest of Europe, USA | **Study designs eligible** Experimental or quasi-experimental, Observational, Qualitative  **Study designs included** Experimental or quasi-experimental  **Number of studies included** 6 |
| Willems (2018)^82^  + Diet | **Intervention** Individual level (Lifestyle change interventions, aimed at physical activity, nutrition or both. Limited information reported)  **Comparator** Any | Physical activity Diet Intermediate outcomes Physical health Mental health and wellbeing Quality of life Environment Other (Activities of daily living) | Any (except laboratory-based)  **Countries eligible** Any country  **Countries included** Not reported | **Study designs eligible** Experimental or quasi-experimental  **Study designs included** Experimental or quasi-experimental  **Number of studies included** 8 |
| **1.4. Unhealthy diet** | | | | |
| Willems (2018) | See people Table A6 section 1.3 (learning disabilities: physical inactivity) | | | |

*PA: physical activity; SES: socio-economic status*

All reviews are systematic reviews.

**Appendix 3: Reviews of barriers and facilitators to behaviour change**

**Table A7 Review characteristics and reported barriers and facilitators**

| **Review** | **Review characteristics** | **Barriers and facilitators to behaviour change** |
| --- | --- | --- |
| **1. Low income/SES** | | |
| **1.1. Tobacco use** | | |
| Hefler (2015)^83^  Low income (age 10-24)  + Disadvantaged areas/communities; Other (socially excluded/ marginalised/ vulnerable youth; young people who have been involved with criminal justice system) | **Countries eligible** Countries at the final stage (stage 4) of the tobacco epidemic  **Countries included** UK, USA, Australia, New Zealand  **Study designs eligible** Qualitative  **Study designs included** Qualitative  **Number of studies included** 17 | **Barriers** Lack of social/ family support: *initiation facilitated by family members; parental acceptance by early-to-mid teens; peer pressure, bullying and physical intimidation to smoke*  Social environment/ cultural norms: s*ense of inhabiting a social context and/or physical environment where exceptions to wider legal and social smoking-related norms applied; smoking seen as normal in the social context; prominent role in peer relations, socialising & often family relationships; smoking tied up with developing and projecting an identity, and a marker to distinguish between groups and indicate group commitment and affiliation; refusing or quitting smoking without an acceptable justification when it was a pervasive norm was equivalent to rejecting friends.* Physical environment: *access to cigarettes in the home (willingly provided or absence of control and perceived lack of rules around smoking); informal markets with older adolescents selling to younger peers at school, procuring cigarettes through proxy sales or targeting stores that were known to be willing to sell to underage young people.* Mental health issues: *in stigmatised communities with few leisure choices and high stress levels, smoking compensated for exclusion and was a way to engage in caring and emotional support.*  Attitudes: *perceived contradictory standard between legal status of tobacco and harms may facilitate flouting the law in relation to supplying young people with cigarettes* Other: *lack of challenge by authority figures, e.g. probation, police, teachers; cannabis as a reverse gateway to smoking tobacco; continued use of cannabis likely to undermine quit attempts*  **Facilitators**  Other: *non-smoking rules in households* |
| Lucherini (2020)^84^  Low SES | **Countries eligible** High income countries at stage 4 of the tobacco epidemic  **Countries included** UK, Australia  **Study designs eligible** Qualitative  **Study designs included** Qualitative  **Number of studies included** 9 | **Barriers** (to using non-combustible nicotine products [NCNP] for harm reduction or cessation)  Social environment/ cultural norms: *smoking perceived as a normal part of everyday life*  Mental health issues: *stress resulting in a return to tobacco (plus the reliability of combustible tobacco compared to NCNP and retention as a last resort*  Financial constraints: *perceived high price of NCNP; cigarettes easily obtainable through informal networks when money tight*  Attitudes: *hedonism; enjoyment of smoking and lack of concern about continued smoking; e-cigarettes sometimes experienced as unsettling because of similarity to smoking.*  Lack of knowledge/ understanding: *lack of clear information on relative harm of NCNP*  Living/ working circumstances: *unemployment- rarely encounter situations of enforced temporary abstinence*  Time constraints: *females prioritise family caregiving so little time for self-care e.g. smoking reduction*  Other: *e-cigarette use dominated by young men- some women reluctant to visit vape shops; NCNP do not carry enough relative advantage over smoking (e.g. taste, less reliable); NCP does not negate necessity for willpower*  **Facilitators**  Attitudes: *novelty technology attractive for some young men- vaper identity; perceived relative advantage of NCNP over cigarettes* Knowledge/ information: *accepted knowledge about relative harm and NCNP* Other: *informal e-cigarette economy to avoid higher prices* |
| Twyman (2014)^85^  Low SES  + Homeless; Prisoners; Other (mental illness; substance misusers; indigenous/native persons; at-risk youth) | **Countries eligible** OECD/ high income/developed countries  **Countries included** UK, Rest of Europe, USA, Australia, Canada, New Zealand  **Study designs eligible** Observational, Qualitative  **Study designs included** Observational, Qualitative  **Number of studies included** 65 (25/64 low SES; 4/65 homeless; 2/65 prisoners) | **Barriers** Lack of social/ family support: *lack of social support; lack of support to quit from health professionals and other service providers (including management and staff in prisons, homeless shelters)*  Social environment/ cultural norms: *high prevalence and acceptability of smoking within vulnerable communities; cultural norms*  Mental health issues: *smoking used as stress management; mental health benefits; loneliness*  Addiction: *addiction to nicotine*  Financial constraints: *socioeconomic disadvantage* Risk/ safety: *concerns regarding treatment* Lack of opportunities or resources: *lack of access to quit resources* Adverse effects of behaviour change: *weight gain*  Motivation: *social activity; rationalisations* Attitudes*: perceived cognitive benefits; enjoyment of smoking; low risk of harm; positive smoker image*  Lack of confidence/self-efficacy: *low confidence; autonomy; past failed attempts*  Living/ working circumstances: *boredom; living and working circumstances*  Habit/ routine  Competing needs: *competing priorities; competing needs and prioritising the need to find shelter/place to live were unique barriers for individuals who were homeless* Other: *other substance use* |
| van Wijk (2019)^86 a^  Low SES | **Countries eligible** Any country  **Countries included** UK, Rest of Europe, USA, Australia, Canada  **Study designs eligible** Not reported  **Study designs included** Experimental or quasi-experimental, Observational, Qualitative, Systematic reviews  **Number of studies included** 43 (including some studies of general population) | **Barriers** (to accessing smoking cessation support) Lack of social/ family support: *lack of social support from social environment & healthcare providers*  Social environment/ cultural norms: *pro-smoking social norm; cultural barriers to seek support, e.g. pressure to accept cigarettes, cultural identity*  Physical environment: *area effect- live in places with limited resources and in isolation from wider community* Transport issues: *low mobility- lack transport/childcare facilities to reach support*  Financial constraints: *financial difficulties & limited reimbursement from health insurance (in countries where free support unavailable)* Lack of opportunities or resources: *cessation support seen as inaccessible- distance & long waiting lists* Issues with services: v*arious barriers associated with smoking cessation support services: poor approachability (professionals provide limited quit advice, poor recruitment); low acceptability; poor availability and accommodation (not locally available, inflexible, fragmented); expensive; appropriateness (e.g. insufficiently intensive, too inflexible, no procedure for relapse, not well targeted at ethnic minorities)* Adverse effects of behaviour change: *weight gain* Motivation: *low motivation to quit* Attitudes: *positive attitudes towards smoking (seen as reward, relaxing, gives sense of control, personal freedom); afraid of failure and being judged for using cessation support- quitting seen as own responsibility, willpower sufficient; negative attitudes towards cessation support; resistance to outsiders complicates recruitment and outreach; cessation support perceived as inappropriate as disallows controlling own destiny*  Lack of knowledge/ understanding: *low risk perception (insufficiently aware of health consequences); lack of knowledge of support available; do not know what to expect from cessation support* Lack of confidence/self-efficacy: *low self-efficacy; high prevalence of unsuccessful quit attempts; negative experiences with former quit attempts* Living/ working circumstances: *smoking as coping mechanism to stressful living conditions, loneliness, boredom*  Habit/ routine: *strong smoking habits* Competing needs: *waiting for the right time to quit; competing priorities, e.g. poor housing, environmental problems, mental health issues* Other: *limited options for alternative activities* |
| **1.2. Physical inactivity** | | |
| Everson-Hock (2013)^18^ ^b^  Low SES  + Unhealthy diet | **Countries eligible** UK  **Countries included** UK  **Study designs eligible** Experimental or quasi-experimental, Observational, Qualitative  **Study designs included** Experimental or quasi-experimental, Observational, Qualitative  **Number of studies included** 35 | **Barriers** Lack of social/ family support: *parental influence*  Social environment/ cultural norms: *traditional food tastes and preferences of family members prioritised*  Physical environment: *lack of local amenities in shopping for healthy foods; food shopping with children and pushchairs, including getting on and off transport and getting to upstairs flats*  Weather: *dark evenings and poor weather*  Mental health issues: *stress, depression*  Financial constraints: *cost of buying healthy food; public transport costs; cost of cooking different meals to suit family preferences; fear of financial risk; lack of prioritisation of healthy food over convenience food when shopping; marketing strategies promoting unhealthy foods; wasting money buying food that the family won’t eat; cost of physical activity* Risk/ safety: *fear of crime and attack* Attitudes: *overweight attributed to flawed metabolism and genetics; no clear perceived link between food and health; seeking cheap, healthy food less of a concern to some; ‘bad’ food seen as a treat and healthy food seen as ‘boring’ and unsatisfying; men prefer to be overweight than ‘thin’; wanting to uphold the right to personal choice through eating unhealthily; unhealthy habits not seen as unhealthy in the light of positive health status; lack of control and perceived self-importance* Lack of knowledge/ understanding: *extent and nature of available information – information bombardment, confused messages, distrust of information; food messages seen as complex (e.g. compared with stop smoking message); misinterpretation of terms such as ‘balanced diet’; misinterpretation of 5-a-day message*  Lack of confidence/self-efficacy; *perceived lack of fitness/sporting capabilities; perceived lack of cooking skills; lack of confidence in cooking meals from scratch; lack of confidence about being able to eat the recommended amount of fruit and vegetables – can be inhibiting and demotivating* Living/ working circumstances: *boredom; living alone*  Habit/ routine: *habit in unhealthy shopping and eating; being stuck in a rut; comfort eating*  Time constraints: *commitments and responsibilities; lack of time*  Other: *embarrassment*  **Facilitators** Family /social support: *participants in interventions can positively influence the health behaviours of family and friends* Motivation: *women motivated to cook healthy food to enhance the health of their children; men motivated to engage in ‘masculine’ behaviours like physical activity to compensate for an unhealthy diet*  Attitudes: *cheap, healthy food was seen as positive by some and actively sought*  Confidence/self-efficacy: *confidence in experimenting with food and cooking*  Knowledge/ information: *TV as source of information, when used positively* |
| Rawal (2020)^87^  Low SES | **Countries eligible**  Industrialised countries  **Countries included** UK, Rest of Europe, USA, Australia, Canada  **Study designs eligible** Qualitative  **Study designs included** Qualitative  **Number of studies included** 19 | **Barriers**  Lack of social/ family support: *guilt as PA considered selfish and low priority by family; weight-related teasing, social misconceptions, and cultural stigma within and between families*  Physical environment: *availability of peers in close proximity; poor neighbourhood appearance* Transport issues: *lack of access to reliable transportation*  Health & disability: *perceived low personal functioning (e.g. due to age, health, disability)*  Financial constraints: *cost of equipment and sports clubs; costs of refreshments, childcare, transportation; membership commitments; perceived stigma when requesting subsidies* Risk/ safety: *threatening situations in environments where exercise may be taken; perceived safety/danger of neighbourhood* Lack of opportunities or resources: *lack of tailored activities- language difficulties and lack of multi-lingual resources* Issues with services: *lack of childcare and inconvenient opening hours of facilities; limited impact of programme marketing*  Attitudes: *transport-related walking perceived as negative experience*  Lack of skills: p*erceived low physical competence* Lack of confidence/self-efficacy: *poor self-image* Time constraints: *work, school, family are priorities; time constraints- inflexible work and family responsibilities*  **Facilitators**  Family /social support: *social influence- support from community networks* Available opportunities: *low-cost PA alternatives (gym subsidies, running outside)* |
| **1.3. Unhealthy diet** |  |  |
| Everson-Hock (2013)  Low SES | See section 1.2 (low income/SES: physical inactivity) | |
| Ohly (2017)^30^  Low income | **Countries eligible** UK, USA  **Countries included** UK, USA  **Study designs eligible** Experimental or quasi-experimental, Observational, Qualitative  **Study designs included** Observational, Qualitative  **Number of studies  included** 38 | **Barriers** (related to use of food vouchers from the Healthy Start or the Special Supplemental Nutrition Program for Women, Infants and Children programmes)  Social environment/ cultural norms: *women who are disempowered are more likely to hand over their Healthy Start vouchers to other family members who then decide how they are used*  Financial constraints Competing needs: *food vouchers may be considered as one part of the household resources and decisions must be made about how best to use the vouchers* |
| Zhang (2020)^32^  Low income (Special Supplemental Nutrition Program for Women, Infants, and Children (WIC) participants) | **Countries eligible** USA  **Countries included** USA  **Study designs eligible** Not reported  **Study designs included** Experimental or quasi-experimental, Observational, Qualitative (Not fully reported)  **Number of studies included** 39 | **Barriers** (to WIC participants’ fruit and vegetable consumption) Financial constraints: *fruit and vegetable cost* Issues with services: *accessibility; negative store redemption experiences (WIC vouchers); varying voucher rules across stores* Lack of knowledge/ understanding: *lack of knowledge about fruits and vegetables*  Time constraints  **Facilitators**  Social environment: *perceptions of fruit and veg as socially desirable*  Services: *flexibility of voucher benefits; inclusion of fresh and processed fruit and vegetables*  Available opportunities Confidence/self-efficacy: *self-efficacy* Knowledge/ information: *fruit and vegetable knowledge* |
| Zorbas (2018)^88^  Low SES^c^ | **Countries eligible** OECD/ high income/developed countries  **Countries included** UK, Rest of Europe, USA, Australia, Canada  **Study designs eligible** Qualitative  **Study designs included** Qualitative  **Number of studies** **included** 39 | **Barriers** Lack of social/ family support: *difficult for families to eat healthfully because easier to oblige to children’s unhealthy food preferences; participants from a low SEP were more likely to describe their social networks as barriers to healthy eating compared with participants in general population groups* Social environment/ cultural norms: *social acceptability of healthy eating generally considered low; preference for spending spare time socializing with family or friends than engaging in food preparation and healthy eating* Physical environment: *presence of tempting unhealthy foods; idea that fast foods are readily available and convenient whenever one is hungry; food insecurity (including low availability of fruit and vegetables) and low access to supermarkets (often exacerbated by limited transportation)*  Weather Transport issues: *limited personal transportation constraining ability to access high-quality supermarkets or markets and to carry purchases; public transport options inconvenient*  Mental health issues: *food cravings, food addiction, and eating for comfort because of life stresses or boredom; lack of mental well-being or the presence of mental health issues*  Addiction  Financial constraints: *high financial price associated with purchasing healthy food; coupons and price promotions within the supermarket setting perceived to incentivise unhealthy food purchases* Risk/ safety: *transportation- concerns about personal safety (e.g. purchases being stolen)* Motivation Attitudes: *acceptability of* *overconsumption during pregnancy; eating out being thought of as treat occasions that permitted unhealthy eating; distrust of scientific nutrition guidelines and information; dieting misperceptions; fear of adverse health outcomes associated with consuming pesticides from fruits and vegetables or the fat content of milk*  Lack of knowledge/ understanding: *doubt over the reputability of information sources; sufficient nutrition literacy required to use nutrition information labels* Lack of skills Lack of confidence/self-efficacy: *negative self-perceptions (or the absence of positive self-perceptions)* Habit/routine Time constraints *&* *the need to plan and prepare healthy meals and foods (especially fruit and vegetables)*  Other: *physiological preferences; marketing and media channels used to heavily promote unhealthy foods and confuse messages around healthy eating; healthy foods such as fruits and vegetables considered unpredictable in terms of perishability and quality*  **Facilitators** Family/social support: *children thought to promote healthy eating by encouraging good role modelling; friends sharing food or helping with meal planning; managers supporting the provision of healthy food within the workplace* Social environment  Physical environment Motivation  Attitudes: *belief that healthy eating could achieve shorter-term benefits of enhanced energy or sport performance; belief that healthy eating could improve an individual’s physical appearance and attractiveness (through weight loss or more generally)*  Skills/ ability: *shopping and food preparation skills; food planning skills; knowing how to shop around* Confidence/self-efficacy & *self-esteem* Knowledge/ information: *nutrition knowledge; food labels; nutrition and cooking information that is accurate, tailored, and accessible*  Health: *witnessing family members experience ill health*  Other: *autonomy* |
| **2. Disadvantaged areas or communities** | | |
| **2.1. Tobacco use** | | |
| Hefler (2015) | See section 1.1. (low income/SES: tobacco use) | |
| **2.2. Physical inactivity** | | |
| Kramer (2017)^89^ | **Countries eligible** Any country  **Countries included** UK, Rest of Europe, USA, Canada, New Zealand  **Study designs eligible** Not reported  **Study designs included** Qualitative  **Number of studies included** 13 | **Barriers** Social environment/ cultural norms: *low levels of social capital constraining social support for walking and social interaction while walking*  Physical environment: *poor aesthetics making walking less relaxing and stress releasing; poor infrastructure makes it inconvenient to walk; lack of leisure-time walking facilities creating a shortage of settings for walking*  Risk/ safety: *safety problems and poor physical neighbourhood design making adults fearful of walking*  **Facilitators**  Family/ social support |
| **3. Homeless people** | | |
| **3.1. Tobacco use** | | |
| Gentry (2019)^67 d^  + Prisoners; Other (mental illness, substance misusers, other stages of criminal justice system) | **Countries eligible** Any country  **Countries included** Rest of Europe, USA, Australia, New Zealand, Other (international online study)  **Study designs eligible** Experimental or quasi-experimental, Observational, Qualitative  **Study designs included** Experimental or quasi-experimental, Observational, Qualitative  **Number of studies included** 9 (0/9 prisoners, 2/9 homeless people) | **Barriers** (to e-cigarette use as a means of smoking cessation/reduction) Social environment/ cultural norms: *fear that e-cigarettes reverse the de-normalisation of smoking*  Mental health issues: *e-cigarettes were perceived to have both beneficial and negative effects on psychiatric symptoms and medication side effects*  Addiction: *concerns about continued or worsening habit/addiction with e-cigarette use*  Financial constraints: *organising finances to retain supplies for constant use of an e-cigarette; expense of e-cigarette personalisation*  Risk/ safety: *safely refilling, charging, and cleaning e-cigarettes; risks of deliberate self-harm from vaping fluid* Adverse effects of behaviour change: *physical side effects (e.g. sore throat)*  **Facilitators**  Family/social support: *family/friends, healthcare professionals, and online posters facilitated e-cigarette initiation and provided moral and practical support* Social environment: *e-cigarettes were socially acceptable and provided a community of “vapers” with opportunities for interaction and connectedness*  Physical environment: *permitted use of e-cigarettes in environments where smoking is banned* Attitudes: *e-cigarettes were more desirable than NRT; e-cigarettes considered less harmful than cigarettes and an alternative source of nicotine for cessation and reduction* Confidence/self-efficacy: *ability to take charge of nicotine addiction was empowering, facilitated by ability to choose and personalise the device* Other: *visible vapour provided an experience similar to smoking which NRT cannot offer; e-cigarettes were perceived to have both beneficial and negative effects on psychiatric symptoms and medication side effects* |
| Twyman (2014) | See section 1.1. (low income/SES: tobacco use) | |
| **4. Prisoners** | | |
| **4.1. Tobacco use** | | |
| Gentry | See section 3.1 (homeless people: tobacco use). No studies on prisoners. | |
| Puljevic (2019)^72 e^ | **Countries eligible** Any country  **Countries included** USA, Australia  **Study designs eligible**  Not reported  **Study designs included** Experimental or quasi-experimental, Observational, Qualitative  **Number of studies included** 15 | **Barriers** Lack of social/ family support: *lack of social support or post-release cessation support* Physical environment: *availability of cigarettes; being surrounded by smokers*  Mental health issues: *stress*  **Facilitators** Attitudes: *negative stigma of smoking; wanting to be a physical role model*  Health: *desire to be physically healthier* Other: *high cost of tobacco* |
| Twyman (2014) | See section 1.1. (low income/SES: tobacco use) | |
| **5. People with learning disabilities** | | |
| **5.1. Physical inactivity** | | |
| Bodde (2009)^90^ | **Countries eligible** Any country  **Countries included** Not reported  **Study designs eligible** Observational, Qualitative  **Study designs included** Observational, Qualitative  **Number of studies included** 7 | **Barriers** Lack of social/ family support: *lack of support/guidance from others (caregivers, authority figures); family member constraints (unavailability)*  Physical environment: *proximity to fitness centres or open space*  Weather Transport issues: *lack of transport*  Financial constraints Risk/ safety: *risk assessment issues; discouragement from others for safety reasons* Lack of opportunities or resources*/ limited options; lack of awareness of the options* Issues with services: *lack of clear policies for engaging in regular activity in residential and day service programs: residential/day care staffing constraints (unavailability, staff's attention directed to consumers with higher needs)* |
| Bossink (2017)^91^ | **Countries eligible** Any country  **Countries included** UK, Rest of Europe, USA, Australia, Canada, Asia  **Study designs eligible** Observational, Qualitative  **Study designs included** Observational, Qualitative  **Number of studies included** 24 | **Barriers**  Lack of social/ family support: *time constraints of parents; family support; staff lack of interest in PA*  Social environment/ cultural norms: *negative societal influences (e.g., discrimination, negative attitudes, and behaviours on the part of others)*  Physical environment: *geographical location and environment*  Weather: *e.g. winter months, cold weather, or rain* Transport issues: *transport difficulties; high stress; cost*  Health & disability: *health issues (e.g., overweight, illness, ear problems, heart conditions); physical disabilities; intellectual disability (lower intellectual functioning); challenging behaviour; ageing*  Financial constraint: *lack of financial support; financial resources of individuals* Risk/ safety: *anxiety on the part of staff and parent; fear (e.g. fear of falling)* Lack of opportunities or resources: *limited options for physical activity; lack of adapted and accessible activities; competitive component of PA; lack of inclusive activities (e.g., segregated leisure facilities)* Issues with services: s*taffing levels; staff expertise (e.g., staff having difficulty thinking of activities); lack of clear policy guidelines in local service agencies; irregular nature of PA programmes; lack of community support (e.g., discontinued classes, lack of acceptance and awareness, high turnover among staff); work routines (e.g., other priorities, staff resistance to change in routines)* Motivation: *lacking motivation; preferences (preferring sedentary behaviour)* Lack of skills: *behavioural skills* Habit/ routine: *resistance to change established routines*  Other: *Physical discomfort*  **Facilitators** Family/ social support: *staff interest in PA (positive support); family support; receiving support from a research team; social interaction (social engagement with peers, friends, or a team)* Social environment: *societal influences*  Services: *staffing level; staff expertise; existence of policy guidelines concerning physical activity; community support (good support, a warm working climate, and encouragement for physical activity within the organisation)*  Available opportunities: *activity with fun component; adapted and accessible activities; inclusive activities; one-to-one nature of programme to meet individual needs; regular nature of physical activity programmes* Physical environment: *geographical location and environment* Weather: *summer* Motivation: *preferences; PA to prevent or reduce health issues; PA to reduce negative behaviour; enjoying travelling* Skills/ ability: *behavioural/social skills; physical abilities; intellectual ability* Other: *having a pet; work routines; activity part of routine; physical comfort (feeling good and energetic); younger age;* *being rewarded* */praised* |
| **6. Refugees or asylum seekers** | | |
| **6.1. Unhealthy diet** | | |
| Elshahat (2020)^92^ ^f^  + Other (Arabic-speaking immigrants) | **Countries eligible** Western societies (North America, Western Europe, Australia, New Zealand)  **Countries included** Rest of Europe *(*Unclear if includes UK evidence), USA, Canada  **Study designs eligible** Experimental or quasi-experimental, Observational, Qualitative  **Study designs included** Observational, Qualitative  **Number of studies included** 24 (21/24 barriers & facilitators) | **Barriers**  Lack of social/ family support: *family preferences for energy-dense, poor nutrient food*  Social environment/ cultural norms: *cultural habits/values; food social stigma; religious dietary proscription*  Physical environment: *high availability of fast/convenience food stores*  Transport issues: *e.g. not owning private car*  Limited availability of healthy food: *lack of food quality/freshness; lack of availability of ethnic food*  Financial constraints *(& expensive healthy food)* Attitudes: *negative attitudes to healthy eating* Lack of knowledge/ understanding: *lack of nutritional knowledge and awareness; literacy/language issues; unfamiliarity with food items in host country, unfamiliarity with grocery store locations/layout* Lack of confidence/ self-efficacy: *low self-efficacy* Time constraints: *family responsibility and time pressures* Other: *lack of genetically modified foods labelling*  **Facilitators**  Family/social support  Social environment: *community cohesion (building social networks with people from mainstream culture)*  Available opportunities: *community educational programmes about healthy eating; community cooking classes* Physical environment: *availability of ethnic stores and traditional food items; bilingual supermarket employees; availability/discounts on healthy food items* Attitudes: *perceived benefits of health eating and risks of unhealthy eating*  Skills/ ability: *increased food and nutrition literacy* Knowledge/ information: *food labels and nutrition labelling*  Financial support  Other: *translated grocery store layouts* |
| Lawlis (2018)^93^ | **Countries eligible** Australia  **Countries included** Australia  **Study designs eligible** Experimental or quasi-experimental, Observational, Qualitative  **Study designs included** Observational, Qualitative  **Number of studies included** 7 | **Barriers**  Social environment/ cultural norms: *adapting to Australian meal patterns- school lunch not seen as important part of child's diet*  Physical environment: *increased availability, food outlet location and cheaper prices of unhealthy foods; relying on small number of food outlets (e.g. food banks) due to location of food outlets; being unsure of where to buy good quality food and difficulties finding traditional and familiar foods*  Transport issues: *transport difficulties in accessing food*  Limited availability of healthy food: *availability of traditional foods, price and variety of food in food outlets; traditional foods available erratically, difficult to purchase or costly; religiously permissible meat difficult to find, resulting in food substitutions or the introduction of new (less healthy) foods*  Financial constraints: *unemployment; low income; limited access to income and welfare entitlements/services* Lack of opportunities or resources: *limited availability of adequate cooking, storage and preparation facilities* Lack of skills: *limited or no preparation and cooking skills (e.g. unfamiliarity with available fruit & veg)* Other: c*hanges in familial responsibilities, e.g. from shared to sole responsibility* |

*NCTP: non-combustible tobacco product; NRT: nicotine replacement therapy; PA: physical activity; SEP: socio-economic position; SES: socio-economic status; WIC: Special Supplemental Nutrition Program for Women, Infants, and Children*

All barriers/facilitators reviews are systematic reviews.

^a^ Review of primary studies and reviews.

^b^ Data reported on barriers and/or facilitators but not a stated objective of the review.

^c^ Included studies of general population as well but concluded that factors identified were generalisable across general and low SES populations or more salient in low SES groups.

^d^ Results not reported separately for homeless people and people with mental illness.

^e^ Focus on smoking after release from smoke-free prisons but includes data on pre-release intentions.

^f^ Results not reported separately for refugees and immigrants.

**Appendix 4: Mapping of behaviours and disadvantaged groups included in reviews**

**
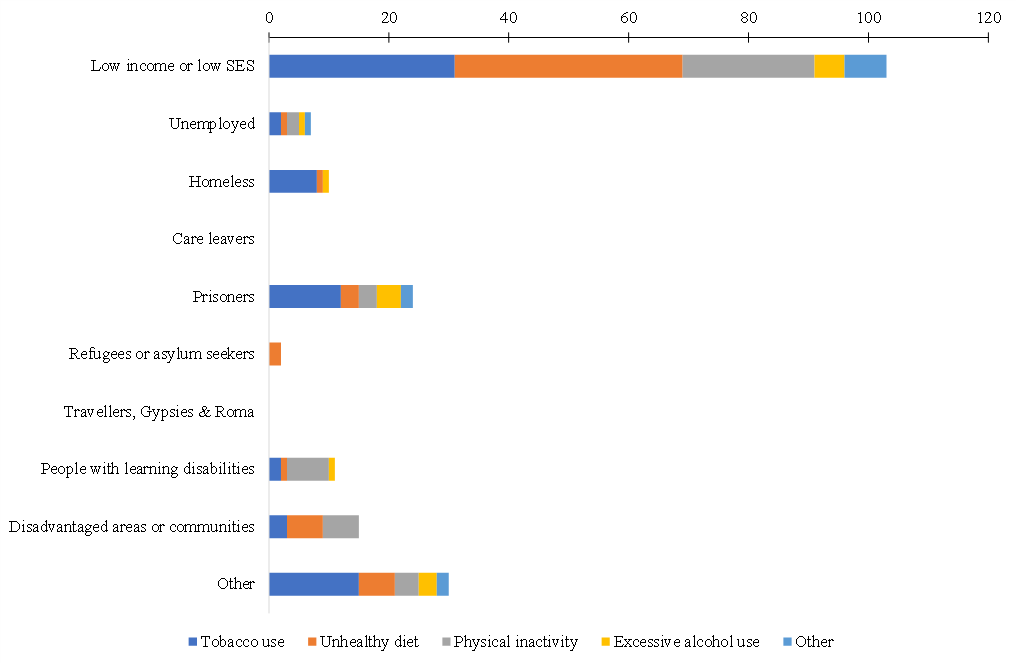
**

**Figure A1 Distribution of included reviews by disadvantaged group and behaviour**

Some reviews targeted more than one behaviour. This chart shows the total number of reviews for each group/behaviour combination rather than the total number of reviews identified for each population group. The ‘Other’ categories refer to behaviours or population groups that were eligible for included reviews but were outside the scope of our review.

**Table A8 Number of included reviews targeting each behaviour and group combination**

|  | **Low income or low SES** | **Unemployed** | **Homeless** | **Care leavers** | **Prisoners** | **Refugees or asylum seekers** | **Travellers, Gypsies & Roma** | **People with learning disabilities** | **Disadvantaged areas or communities** | **Other** |
| --- | --- | --- | --- | --- | --- | --- | --- | --- | --- | --- |
| Tobacco use | 31 | 2 | 8 | 0 | 12 | 0 | 0 | 2 | 3 | 15 |
| Unhealthy diet | 38 | 1 | 1 | 0 | 3 | 2 | 0 | 1 | 6 | 6 |
| Physical inactivity | 22 | 2 | 0 | 0 | 3 | 0 | 0 | 7 | 6 | 4 |
| Excessive alcohol use | 5 | 1 | 1 | 0 | 4 | 0 | 0 | 1 | 0 | 3 |
| Other | 7 | 1 | 0 | 0 | 2 | 0 | 0 | 0 | 0 | 2 |

Some reviews targeted multiple behaviours and/or groups. The ‘Other’ categories refer to behaviours or population groups that were eligible for included reviews but were outside the scope of our review.

**
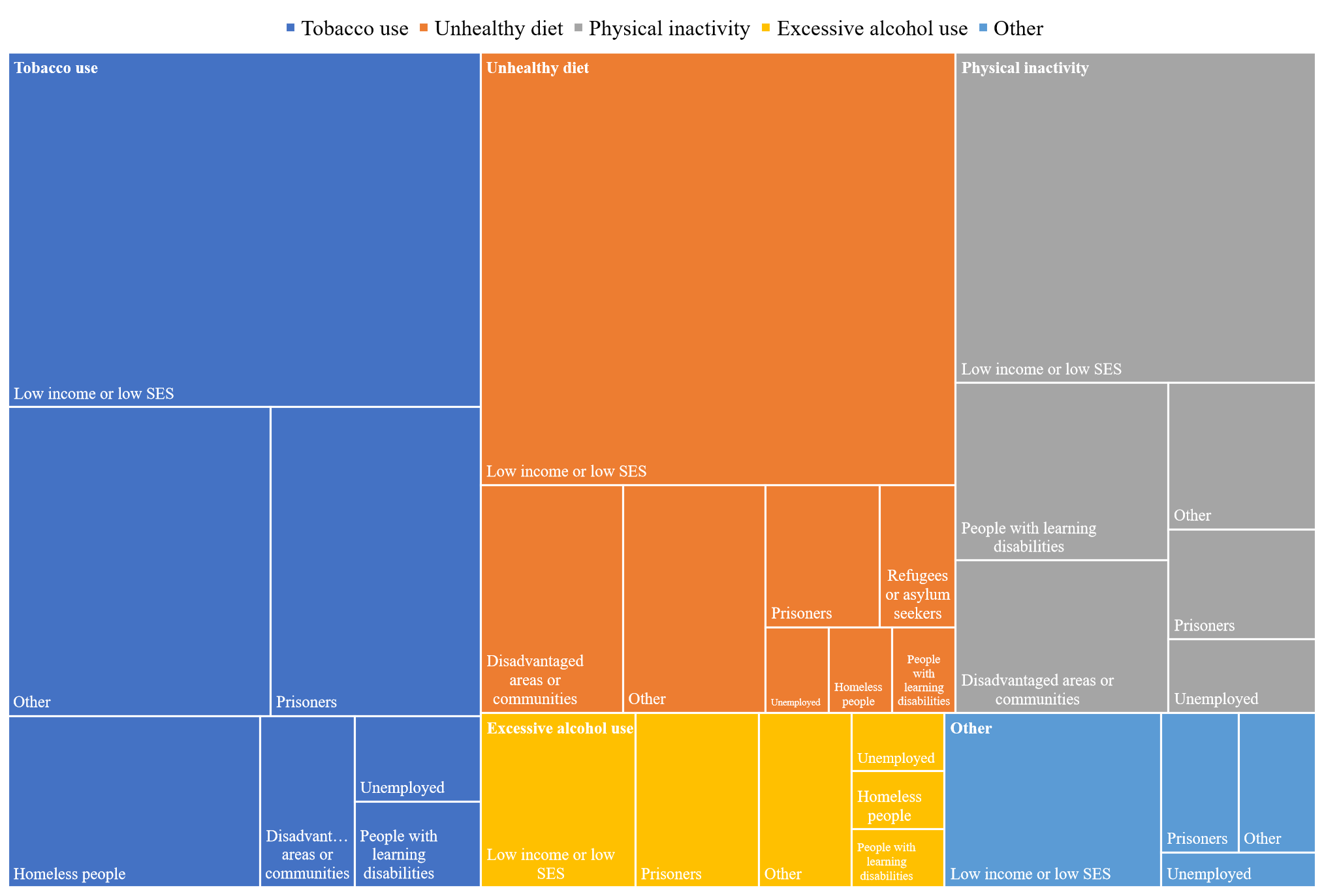
**

**Figure A2 Distribution of included reviews by behaviour and disadvantaged group**

Each rectangle represents the total number of reviews that included the specific behaviour/group combination. Some reviews included more than one behaviour and/or group and are represented in multiple rectangles. The ‘Other’ categories refer to behaviours or population groups included in reviews that were outside the scope of our review.

**References**

1. Boland VC, Stockings EA, Mattick RP, McRobbie H, Brown J, Courtney RJ. The Methodological Quality and Effectiveness of Technology-Based Smoking Cessation Interventions for Disadvantaged Groups: A Systematic Review and Meta-analysis. *Nicotine Tob Res*; **20(3):** 276–85, <https://doi.org/10.1093/ntr/ntw391>

2. Bryant J, Bonevski B, Paul C, McElduff P, Attia J. A systematic review and meta-analysis of the effectiveness of behavioural smoking cessation interventions in selected disadvantaged groups. *Addiction* 2011; **106(9):**1568–85, <https://doi.org/10.1111/j.1360-0443.2011.03467.x>

3. Bull ER, Dombrowski SU, McCleary N, Johnston M. Are interventions for low-income groups effective in changing healthy eating, physical activity and smoking behaviours? A systematic review and meta-analysis. *BMJ Open* 2014; **4(11)**: e006046, <https://doi.org/10.1136/bmjopen-2014-006046>

4. Ford P, Clifford A, Gussy K, Gartner C. A systematic review of peer-support programs for smoking cessation in disadvantaged groups. *International Journal of Environmental Research & Public Health* 2013; **10(11):** 5507–22, <https://doi.org/10.3390/ijerph10115507>

5. Kock L, Brown J, Hiscock R, Tattan-Birch H, Smith C, Shahab L. Individual-level behavioural smoking cessation interventions tailored for disadvantaged socioeconomic position: a systematic review and meta-regression. *Lancet Public Health* 2019; **4(12)**: e628–44, <https://doi.org/10.1016/S2468-2667(19)30220-8>

6. Michie S, Jochelson K, Markham WA, Bridle C. Low-income groups and behaviour change interventions: a review of intervention content, effectiveness and theoretical frameworks. *Journal of Epidemiology & Community Health* 2009; **63(8)**: 610–22, <https://doi.org/10.1136/jech.2008.078725>

7. Murray RL, Bauld L, Hackshaw LE, McNeill A. Improving access to smoking cessation services for disadvantaged groups: a systematic review. *Journal of Public Health* 2009; **31(2):** 258–77, <https://doi.org/10.1093/pubmed/fdp008>

8. O'Mara-Eves A, Brunton G, Oliver S, Kavanagh J, Jamal F, Thomas J. The effectiveness of community engagement in public health interventions for disadvantaged groups: a meta-analysis. *BMC Public Health* 2015; **15:** 129, <https://doi.org/10.1186/s12889-015-1352-y>

9. O'Mara-Eves A, Brunton G, McDaid D, Oliver S, Kavanagh J, Jamal F, et al. Community engagement to reduce inequalities in health: a systematic review, meta-analysis and economic analysis. *Public Health Res* 2013; **1(4)**, <https://doi.org/10.3310/phr01040>

10. Smith P, Poole R, Mann M, Nelson A, Moore G, Brain K. Systematic review of behavioural smoking cessation interventions for older smokers from deprived backgrounds. *BMJ Open* 2019; **9(11):** e032727, <https://doi.org/10.1136/bmjopen-2019-032727>

11. Smith CE, Hill SE, Amos A. Impact of specialist and primary care stop smoking support on socio-economic inequalities in cessation in the United Kingdom: a systematic review and national equity analysis. *Addiction* 2020; **115(1):** 34–46, <https://doi.org/10.1111/add.14760>

12. Stiehl E, Shivaprakash N, Thatcher E, Ornelas IJ, Kneipp S, Baron SL, et al. Worksite Health Promotion for Low-Wage Workers: A Scoping Literature Review. *Am J Health Promot* 2018; **32(2):** 359–73, <https://doi.org/10.1177/0890117117728607>

13. Walton-Moss B, Samuel L, Nguyen TH, Commodore-Mensah Y, Hayat MJ, Szanton SL. Community-based cardiovascular health interventions in vulnerable populations: a systematic review. *J Cardiovasc Nurs*; **29(4):** 293–307, <https://doi.org/10.1097/JCN.0b013e31828e2995>

14. Wilson A, Guillaumier A, George J, Denham A, Bonevski B. A systematic narrative review of the effectiveness of behavioural smoking cessation interventions in selected disadvantaged groups (2010-2017). *Expert Rev Respir Med* 2017; **11(8):** 617–30, <https://doi.org/10.1080/17476348.2017.1340836>

15. Burke E, Dobbie F, Dougall N, Adebolu Oluwaseun M, Mockler D, Vance J, et al. Smoking cessation programmes for women living in disadvantaged communities, "We Can Quit 2": A systematic review protocol. *HRB open res* 2019; **2:** 10, <https://doi.org/10.12688/hrbopenres.12901.1>

16. Muckle W, Muckle J, Welch V, Tugwell P. Managed alcohol as a harm reduction intervention for alcohol addiction in populations at high risk for substance abuse. *Cochrane Database of Systematic Reviews* 2012; **12:** CD006747, <https://doi.org/10.1002/14651858.CD006747.pub2>

17. Cleland V, Granados A, Crawford D, Winzenberg T, Ball K. Effectiveness of interventions to promote physical activity among socioeconomically disadvantaged women: a systematic review and meta-analysis. *Obes Rev* 2013; **14(3):** 197–212, <https://doi.org/10.1111/j.1467-789X.2012.01058.x>

18. Everson-Hock ES, Johnson M, Jones R, Woods HB, Goyder E, Payne N, et al. Community-based dietary and physical activity interventions in low socioeconomic groups in the UK: a mixed methods systematic review. *Prev Med* 2013; **56(5):** 265–72 <https://doi.org/10.1016/j.ypmed.2013.02.023>

19. Olstad DL, Ancilotto R, Teychenne M, Minaker LM, Taber DR, Raine KD, et al. Can targeted policies reduce obesity and improve obesity-related behaviours in socioeconomically disadvantaged populations? A systematic review. *Obes Rev* 2017; **18(7):** 791–807, <https://doi.org/10.1111/obr.12546>

20. Stormacq C, Wosinski J, Boillat E, Van den Broucke S. Effects of health literacy interventions on health-related outcomes in socioeconomically disadvantaged adults living in the community: a systematic review. *JBI Evid Synth* 2020; **18(7):** 1389–469, <https://doi.org/10.11124/JBISRIR-D-18-00023>

21. An R, Wang J, Liu J, Shen J, Loehmer E, McCaffrey J. A systematic review of food pantry-based interventions in the USA. *Public Health Nutr* 2019**; 22(9):**1704–16, <https://doi.org/10.1017/S1368980019000144>

22. Black AP, Brimblecombe J, Eyles H, Morris P, Vally H, K OD. Food subsidy programs and the health and nutritional status of disadvantaged families in high income countries: a systematic review. *BMC Public Health* 2012; **12:** 1099, <https://doi.org/10.1186/1471-2458-12-1099>

23. De Marchis EH, Torres JM, Benesch T, Fichtenberg C, Allen IE, Whitaker EM, et al. Interventions Addressing Food Insecurity in Health Care Settings: A Systematic Review. *Ann Fam Med* 2019; **17(5):** 436–47, <https://doi.org/10.1370/afm.2412>

24. Eicher-Miller HA. A review of the food security, diet and health outcomes of food pantry clients and the potential for their improvement through food pantry interventions in the United States. *Physiol Behav* 2020; **220:** 112871, <https://doi.org/10.1016/j.physbeh.2020.112871>

25. Engel K, Ruder EH. Fruit and Vegetable Incentive Programs for Supplemental Nutrition Assistance Program (SNAP) Participants: A Scoping Review of Program Structure. *Nutrients* 2020; **12(6):** 1676, <https://doi.org/10.3390/nu12061676>

26. Hsiao BS, Sibeko L, Troy LM. A Systematic Review of Mobile Produce Markets: Facilitators and Barriers to Use, and Associations with Reported Fruit and Vegetable Intake. *J Acad Nutr Diet* 2019; **119(1):** 76–97.e1, <https://doi.org/10.1016/j.jand.2018.02.022>

27. Iacovou M, Pattieson DC, Truby H, Palermo C. Social health and nutrition impacts of community kitchens: a systematic review. *Public Health Nutr* 2013; **16(3):** 535–43, <https://doi.org/10.1017/S1368980012002753>

28. Langellier BA, Garza JR, Prelip ML, Glik D, Brookmeyer R, Ortega AN. Corner Store Inventories, Purchases, and Strategies for Intervention: A Review of the Literature. *Calif J Health Promot* 2013; **11(3):** 1–13, <https://doi.org/10.32398/cjhp.v11i3.1537>

29. Long CR, Rowland B, Steelman SC, McElfish PA. Outcomes of disease prevention and management interventions in food pantries and food banks: a scoping review. *BMJ Open* 2019; **9(8):** e029236, <https://doi.org/10.1136/bmjopen-2019-029236>

30. Ohly H, Crossland N, Dykes F, Lowe N, Hall-Moran V. A realist review to explore how low-income pregnant women use food vouchers from the UK's Healthy Start programme. *BMJ Open* 2017; **7(4):** e013731., <https://doi.org/10.1136/bmjopen-2016-013731>

31. Verghese A, Raber M, Sharma S. Interventions targeting diet quality of Supplemental Nutrition Assistance Program (SNAP) participants: A scoping review. *Prev Med* 2019; **119:** 77–86, <https://doi.org/10.1016/j.ypmed.2018.12.006>

32. Zhang Q, Alsuliman MA, Wright M, Wang Y, Cheng X. Fruit and Vegetable Purchases and Consumption among WIC Participants after the 2009 WIC Food Package Revision: A Systematic Review. Adv Nutr 2020; **11(6):** 1646–62, <https://doi.org/10.1093/advances/nmaa060>

33. Guillaumier A, Bonevski B, Paul C. Anti-tobacco mass media and socially disadvantaged groups: a systematic and methodological review. *Drug Alcohol Rev* 2012; **31(5):** 698–708, <https://doi.org/10.1111/j.1465-3362.2012.00466.x>

34. Hill S, Amos A, Clifford D, Platt S. Impact of tobacco control interventions on socioeconomic inequalities in smoking: review of the evidence. *Tob Control* 2014; **23(e2):** e89-97, <https://doi.org/10.1136/tobaccocontrol-2013-051110>

35. Craike M, Wiesner G, Hilland TA, Bengoechea EG. Interventions to improve physical activity among socioeconomically disadvantaged groups: an umbrella review. *International Journal of Behavioral Nutrition and Physical Activity* 2018; **15(1):** 43, <https://doi.org/10.1186/s12966-018-0676-2>

36. Bader P, Boisclair D, Ferrence R. Effects of tobacco taxation and pricing on smoking behavior in high risk populations: a knowledge synthesis. *International Journal of Environmental Research & Public Health* 2011; **8(11):** 4118–39, <https://doi.org/10.3390/ijerph8114118>

37. Beauchamp A, Peeters A, Tonkin A, Turrell G. Best practice for prevention and treatment of cardiovascular disease through an equity lens: a review. *Eur J Cardiovasc Prev Rehabil* 2010; **17(5):** 599–606, <https://doi.org/10.1097/HJR.0b013e328339cc99>

38. Bell K, McCullough L, Devries K, Jategaonkar N, Greaves L, Richardson L. Location restrictions on smoking: assessing their differential impacts and consequences in the workplace. *Can J Public Health* 2009; **100(1):** 46–50, <https://doi.org/10.1007/BF03405492>

39. Brown T, Platt S, Amos A. Equity impact of population-level interventions and policies to reduce smoking in adults: a systematic review*. Drug Alcohol Depend* 2014; **138:** 7–16, <https://doi.org/10.1016/j.drugalcdep.2014.03.001>

40. Brown T, Platt S, Amos A. Equity impact of interventions and policies to reduce smoking in youth: systematic review. *Tob Control* 2014; **23(e2):** e98–105, <https://doi.org/10.1136/tobaccocontrol-2013-051451>

41. Durkin S, Brennan E, Wakefield M. Mass media campaigns to promote smoking cessation among adults: an integrative review. *Tob Control* 2012; **21(2):** 127–38, <https://doi.org/10.1136/tobaccocontrol-2011-050345>

42. Mozaffarian D, Afshin A, Benowitz NL, Bittner V, Daniels SR, Franch HA, et al. Population approaches to improve diet, physical activity, and smoking habits: a scientific statement from the American Heart Association. *Circulation* 2012; **126(12):** 1514–63, <https://doi.org/10.1161/CIR.0b013e318260a20b>

43. Jepson RG, Harris FM, Platt S, Tannahill C. The effectiveness of interventions to change six health behaviours: a review of reviews. *BMC Public Health* 2010; **10:** 538, <https://doi.org/10.1186/1471-2458-10-538>

44. Lorenc T, Petticrew M, Welch V, Tugwell P. What types of interventions generate inequalities? Evidence from systematic reviews. *Journal of Epidemiology & Community Health* 2013; **67(2):** 190–3, <https://doi.org/10.1136/jech-2012-201257>

45. Stead M, Angus K, Langley T, Katikireddi SV, Hinds K, Hilton S, et al. Mass media to communicate public health messages in six health topic areas: a systematic review and other reviews of the evidence. *Public Health Res* 2019; **7(8),** <https://doi.org/10.3310/phr07080>

46. Thomson K, Hillier-Brown F, Todd A, McNamara C, Huijts T, Bambra C. The effects of public health policies on health inequalities in high-income countries: an umbrella review. *BMC Public Health* 2018; **18(1):** 869, <https://doi.org/10.1186/s12889-018-5677-1>

47. Baker PR, Francis DP, Soares J, Weightman AL, Foster C. Community wide interventions for increasing physical activity. *Cochrane Database of Systematic Reviews* 2015; **1:** CD008366, <https://doi.org/10.1002/14651858.CD008366.pub3>

48. Boelsen-Robinson T, Peeters A, Beauchamp A, Chung A, Gearon E, Backholer K. A systematic review of the effectiveness of whole-of-community interventions by socioeconomic position. *Obes Rev* 2015; **16(9):** 806–16, <http://doi.org/10.1111/obr.12297>

49. Olstad DL, Teychenne M, Minaker LM, Taber DR, Raine KD, Nykiforuk CI, et al. Can policy ameliorate socioeconomic inequities in obesity and obesity-related behaviours? A systematic review of the impact of universal policies on adults and children. *Obes Rev* 2016; **17(12):** 1198–217, <http://doi.org/10.1111/obr.12457>

50. Smith M, Hosking J, Woodward A, Witten K, MacMillan A, Field A, et al. Systematic literature review of built environment effects on physical activity and active transport - an update and new findings on health equity. *International Journal of Behavioral Nutrition and Physical Activity* 2017; **14(1):** 158, <https://doi.org/10.1186/s12966-017-0613-9>

51. Thomas MM, Phongsavan P, McGill B, O'Hara BJ, Bauman AE. A review of the impact of physical activity mass media campaigns on low compared to high socioeconomic groups. *Health Educ Re* 2018; **33(5):** 429–46, <https://doi.org/10.1093/her/cyy032>

52. Baker PRA, Dobbins M, Soares J, Francis DP, Weightman AL, Costello JT. Public health interventions for increasing physical activity in children, adolescents and adults: An overview of systematic reviews. *Cochrane Database of Systematic Reviews* 2015; **1:** CD011454, <http://doi.org/10.1002/14651858.CD011454>

53. Tully MA, Kee F, Foster C, Cardwell CR, Weightman AL, Cupples ME. Built environment interventions for increasing physical activity in adults and children. *Cochrane Database of Systematic Reviews* 2013; **1:** CD010330, <http://doi.org/10.1002/14651858.CD010330>

54. Backholer K, Sarink D, Beauchamp A, Keating C, Loh V, Ball K, et al. The impact of a tax on sugar-sweetened beverages according to socio-economic position: a systematic review of the evidence. *Public Health Nutr* 2016; **19(17):** 3070–84, <http://doi.org/10.1017/S136898001600104X>

55. Hillier-Brown FC, Summerbell CD, Moore HJ, Routen A, Lake AA, Adams J, et al. The impact of interventions to promote healthier ready-to-eat meals (to eat in, to take away or to be delivered) sold by specific food outlets open to the general public: a systematic review. *Obes Rev* 2017; **18(2):** 227–46, <https://doi.org/10.1111/obr.12479>

56. Lobstein T, Neveux M, Landon J. Costs, equity and acceptability of three policies to prevent obesity: A narrative review to support policy development. *Obesity Science and Practice* 2020; **6:** 562–83, <http://doi.org/10.1002/osp4.423>

57. McGill R, Anwar E, Orton L, Bromley H, Lloyd-Williams F, O'Flaherty M, et al. Are interventions to promote healthy eating equally effective for all? Systematic review of socioeconomic inequalities in impact. *BMC Public Health* 2015; **15:** 457, <https://doi.org/10.1186/s12889-015-1781-7>

58. McLaren L, Sumar N, Barberio AM, Trieu K, Lorenzetti DL, Tarasuk V, et al. Population-level interventions in government jurisdictions for dietary sodium reduction. *Cochrane Database of Systematic Reviews* 2016; **9:** CD010166, <https://doi.org/10.1002/14651858.CD010166.pub2>

59. Pfinder M, Heise TL, Hilton Boon M, Pega F, Fenton C, Griebler U, et al. Taxation of unprocessed sugar or sugar-added foods for reducing their consumption and preventing obesity or other adverse health outcomes. *Cochrane Database of Systematic Reviews* 2020; **4:** CD012333, <https://doi.org/10.1002/14651858.CD012333.pub2>

60. Sarink D, Peeters A, Freak-Poli R, Beauchamp A, Woods J, Ball K, et al. The impact of menu energy labelling across socioeconomic groups: A systematic review. *Appetite* 2016; **99:** 59–75, <https://doi.org/10.1016/j.appet.2015.12.022>

61. Thow AM, Downs S, Jan S. A systematic review of the effectiveness of food taxes and subsidies to improve diets: understanding the recent evidence. *Nutr Rev* 2014; **72(9):** 551–65, <https://doi.org/10.1111/nure.12123>

62. von Philipsborn P, Stratil JM, Burns J, Busert LK, Pfadenhauer LM, Polus S, et al. Environmental interventions to reduce the consumption of sugar-sweetened beverages and their effects on health. *Cochrane Database of Systematic Reviews* 2019; **6:** CD012292, <https://doi.org/10.1002/14651858.CD012292.pub2>

63. Vargas-Garcia EJ, El Evans C, Cade JE. Impact of interventions to reduce sugar-sweetened beverage intake in children and adults: a protocol for a systematic review and meta-analysis. *Systematic Reviews* 2015; **4:** 17, <https://doi.org/10.1186/s13643-015-0008-4>

64. Cleland CL, Tully MA, Kee F, Cupples ME. The effectiveness of physical activity interventions in socio-economically disadvantaged communities: a systematic review. *Prev Med* 2012; **54(6):** 371–80, <https://doi.org/10.1016/j.ypmed.2012.04.004>

65. Hollis-Hansen K, Vermont L, Zafron ML, Seidman J, Leone L. The introduction of new food retail opportunities in lower-income communities and the impact on fruit and vegetable intake: a systematic review. *Transl Behav Med* 2019; **9(5):** 837–46, <https://doi.org/10.1093/tbm/ibz094>

66. Hollederer A. Health promotion and prevention among the unemployed: a systematic review. *Health Promot Int* 2019; **34(6):** 1078–96, <https://doi.org/10.1093/heapro/day069>

67. Gentry S, Forouhi NG, Notley C. Are Electronic Cigarettes an Effective Aid to Smoking Cessation or Reduction Among Vulnerable Groups? A Systematic Review of Quantitative and Qualitative Evidence. *Nicotine Tob Res* 2019; **21(5):** 602–16, <https://doi.org/10.1093/ntr/nty054>

68. Ijaz S, Thorley H, Porter K, Fleming C, Jones T, Kesten J, et al. Interventions for preventing or treating malnutrition in homeless problem-drinkers: a systematic review. *International Journal for Equity in Health* 2018; **17(1):** 8, <https://doi.org/10.1186/s12939-018-0722-3>

69. de Andrade D, Kinner SA. Systematic review of health and behavioural outcomes of smoking cessation interventions in prisons. *Tob Control* 2016; **26(5):** 495–501, <https://doi.org/10.1136/tobaccocontrol-2016-053297>

70. Frazer K, McHugh J, Callinan JE, Kelleher C. Impact of institutional smoking bans on reducing harms and secondhand smoke exposure. *Cochrane Database of Systematic Reviews* 2016; **5:** CD011856, <https://doi.org/10.1002/14651858.CD011856.pub2>

71. Mohan ARM, Thomson P, Leslie SJ, Dimova E, Haw S, McKay JA. A Systematic Review of Interventions to Improve Health Factors or Behaviors of the Cardiovascular Health of Prisoners During Incarceration. *J Cardiovasc Nurs* 2018; **33(1):** 72–81, <https://doi.org/10.1097/JCN.0000000000000420>

72. Puljevic C, Segan CJ. Systematic Review of Factors Influencing Smoking Following Release From Smoke-Free Prisons. *Nicotine Tob Res* 2019; **21(8):** 1011–20, <https://doi.org/10.1093/ntr/nty088>

73. Wright N, Bleakley A, Butt C, Chadwick O, Mahmood K, Patel K, et al. Peer health promotion in prisons: a systematic review. *Int J Prison Health* 2011; **7(4):** 37–51, <https://doi.org/10.1108/17449201111256899>

74. Almondes N, Downie D, Cinar AB, Richards D, Freeman R. Is health coaching effective in changing the health status and behaviour of prisoners?-a systematic review protocol. *Systematic Reviews* 2017; **6(1):** 127, <https://doi.org/10.1186/s13643-017-0524-5>

75. Newbury-Birch D, McGovern R, Birch J, O'Neill G, Kaner H, Sondhi A, et al. A rapid systematic review of what we know about alcohol use disorders and brief interventions in the criminal justice system. *Int J Prison Health* 2016; **12(1):** 57–70, <https://doi.org/10.1108/IJPH-08-2015-0024>

76. Newbury-Birch D, Ferguson J, Landale S, Giles EL, McGeechan GJ, Gill C, et al. A Systematic Review of the Efficacy of Alcohol Interventions for Incarcerated People. *Alcohol Alcohol* 2018; **53(4):** 412–25, <https://doi.org/10.1093/alcalc/agy032>

77. Kerr S, Lawrence M, Darbyshire C, Middleton AR, Fitzsimmons L. Tobacco and alcohol-related interventions for people with mild/moderate intellectual disabilities: a systematic review of the literature. *J Intellect Disabil Res* 2013; **57(5):** 393–408, <https://doi.org/10.1111/j.1365-2788.2012.01543.x>

78. Bondar RZ, di Fronso S, Bortoli L, Robazza C, Metsios GS, Bertollo M. The effects of physical activity or sport-based interventions on psychological factors in adults with intellectual disabilities: a systematic review. *J Intellect Disabil Res* 2020; **64(2):** 69–92, <https://doi.org/10.1111/jir.12699>

79. Brooker K, van Dooren K, McPherson L, Lennox N, Ware R. A systematic review of interventions aiming to improve involvement in physical activity among adults with intellectual disability. *J Phys Act Health* 2015; **12(3):** 434–44, <https://doi.org/10.1123/jpah.2013-0014>

80. Hassan NM, Landorf KB, Shields N, Munteanu SE. Effectiveness of interventions to increase physical activity in individuals with intellectual disabilities: a systematic review of randomised controlled trials. *J Intellect Disabil Res* 2019; **63(2):**168–91, <https://doi.org/10.1111/jir.12562>

81. Temple VA, Frey GC, Stanish HI. Interventions to promote physical activity for adults with intellectual disabilities. *Salud Publica Mex* 2017; **59(4):** 446–53, <https://doi.org/10.21149/8218>

82. Willems M, Waninge A, Hilgenkamp TIM, van Empelen P, Krijnen WP, van der Schans CP, et al. Effects of lifestyle change interventions for people with intellectual disabilities: Systematic review and meta-analysis of randomized controlled trials. *J Appl Res Intellect Disabil* 2018; **31(6):** 949–61, <https://doi.org/110.1111/jar.12463>

83. Hefler M, Chapman S. Disadvantaged youth and smoking in mature tobacco control contexts: a systematic review and synthesis of qualitative research. *Tob Control* 2015; **24(5):** 429–35, <https://doi.org/10.1136/tobaccocontrol-2014-051756>

84. Lucherini M, Hill S, Smith K. Inequalities, harm reduction and non-combustible nicotine products: a meta-ethnography of qualitative evidence. *BMC Public Health* 2020; **20(1):** 943, <https://doi.org/10.1186/s12889-020-09083-9>

85. Twyman L, Bonevski B, Paul C, Bryant J. Perceived barriers to smoking cessation in selected vulnerable groups: a systematic review of the qualitative and quantitative literature. *BMJ Open* 2014; **4(12):** e006414, <https://doi.org/10.1136/bmjopen-2014-006414>

86. van Wijk EC, Landais LL, Harting J. Understanding the multitude of barriers that prevent smokers in lower socioeconomic groups from accessing smoking cessation support: A literature review. *Prev Med* 2019; **123:** 143-51, <https://doi.org/10.1016/j.ypmed.2019.03.029>

87. Rawal LB, Smith BJ, Quach H, Renzaho AMN. Physical Activity among Adults with Low Socioeconomic Status Living in Industrialized Countries: A Meta-Ethnographic Approach to Understanding Socioecological Complexities. *J Environ Public Health* 2020; **2020:** 4283027, <https://doi.org/10.1155/2020/4283027>

88. Zorbas C, Palermo C, Chung A, Iguacel I, Peeters A, Bennett R, et al. Factors perceived to influence healthy eating: a systematic review and meta-ethnographic synthesis of the literature*. Nutr Rev* 2018; **76(12):** 861–74, <https://doi.org/10.1093/nutrit/nuy043>

89. Kramer D, Lakerveld J, Stronks K, Kunst AE. Uncovering How Urban Regeneration Programs May Stimulate Leisure-time Walking Among Adults in Deprived Areas: A Realist Review. *Int J Health Serv* 2017; **47(4):** 703–24, <https://doi.org/10.1177/0020731417722087>

90. Bodde AE, Seo DC. A review of social and environmental barriers to physical activity for adults with intellectual disabilities. *Disabil Health J* 2009; **2(2):** 57–66, <https://doi.org/10.1016/j.dhjo.2008.11.004>

91. Bossink LWM, van der Putten AA, Vlaskamp C. Understanding low levels of physical activity in people with intellectual disabilities: A systematic review to identify barriers and facilitators. *Res Dev Disabil* 2017; **68:** 95–110, <https://doi.org/10.1016/j.ridd.2017.06.008>

92. Elshahat S, Moffat T. Dietary practices among Arabic-speaking immigrants and refugees in Western societies: A scoping review. *Appetite* 2020; **154:** 104753, <https://doi.org/10.1016/j.appet.2020.104753>

93. Lawlis T, Islam W, Upton P. Achieving the four dimensions of food security for resettled refugees in Australia: A systematic review. *Nutr Diet* 2018; **75(2):** 182–92, <https://doi.org/10.1111/1747-0080.12402>
